# A new approach to the asymmetric Mannich reaction catalyzed by chiral *N*,*N*′-dioxide–metal complexes†
†Electronic supplementary information (ESI) available. CCDC 1448521 and 1480808.For ESI and crystallographic data in CIF or other electronic format see DOI: 10.1039/c6sc03902b



**DOI:** 10.1039/c6sc03902b

**Published:** 2016-10-03

**Authors:** Xiangjin Lian, Lili Lin, Kai Fu, Baiwei Ma, Xiaohua Liu, Xiaoming Feng

**Affiliations:** a Key Laboratory of Green Chemistry & Technology , Ministry of Education , College of Chemistry , Sichuan University , Chengdu 610064 , P. R. China . Email: xmfeng@scu.edu.cn ; Fax: +86 28 85418249 ; Tel: +86 28 85418249

## Abstract

A highly efficient asymmetric Mannich-type reaction between α-tetralone-derived β-keto esters/amides and 1,3,5-triaryl-1,3,5-triazinanes was realized in the presence of chiral *N*,*N*′-dioxide–Ni(ii) or Mg(ii) complex. A variety of optically active β-amino compounds with all-carbon quaternary stereocenters were obtained in good yields with excellent enantioselectivities. A possible transition state was proposed based on these experiments and previous reports.

## Abstract 2

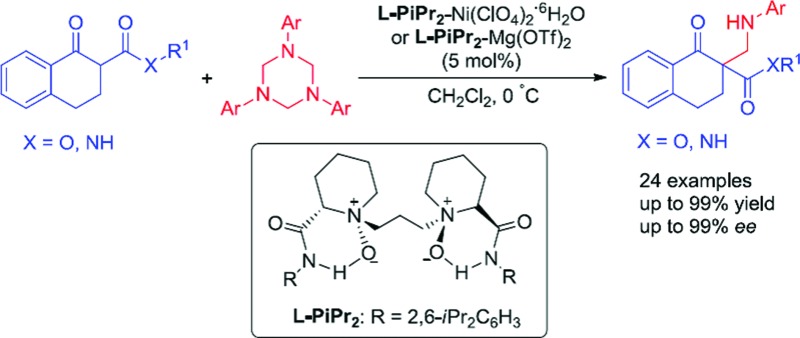
An efficient asymmetric Mannich-type reaction between α-tetralone-derived β-keto esters/amides and 1,3,5-triaryl-1,3,5-triazinanes was realized catalyzed by chiral *N*,*N*'–dioxide-Ni(ii)/Mg(ii) complexes.

Because the resulting nitrogen-containing compounds are widely distributed in nature and include many biologically important molecules,^1^ the Mannich reaction has received a lot of attention since its discovery in the early 20th century (Scheme 1a).^2^ It has become one of the most efficient methods to construct C–C bonds.^3^ Despite its important synthetic value, the development of the classical intermolecular Mannich reaction has been plagued by a number of serious disadvantages such as the undesired side products formed in many cases, and the ability to control the regio- and stereoselectivity is generally unsatisfactory.^4^ The first catalytic enantioselective approach was reported by Kobayashi using a novel chiral zirconium catalyst in 1997.^5^ To overcome the drawbacks of the classical Mannich reaction, preformed Mannich reagents such as imines and iminium salts have been developed (Scheme 1b).^6^ Subsequently, the catalytic asymmetric Mannich reaction has received a certain amount of development.^7^ However, such preformed Mannich reagents also have some defects such as low activity, sensitivity to moisture and instability, and therefore the development of new Mannich reagents is desirable.

**Scheme 1:**
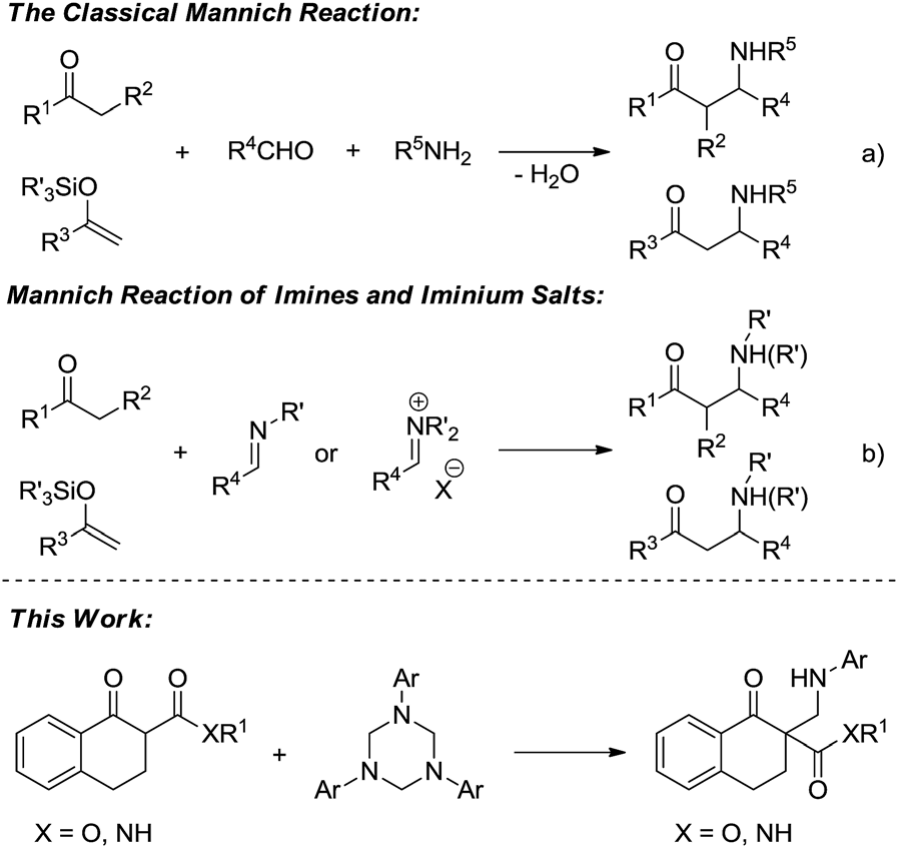
Classical Mannich-type reaction and the new approach.

1,3,5-Triaryl-1,3,5-triazinanes, which are conveniently prepared through the condensation of paraformaldehyde and aromatic amines,^8^ can generate the corresponding imines in solvent, which can be used as Mannich reagents. Very recently, Krische reported investigations on the hydroaminomethylation of allenes and 1,3-dienes with 1,3,5-triaryl-1,3,5-triazinanes catalyzed by ruthenium.^9^ Inspired by Krische's work, we think that the *in situ* generated imines from 1,3,5-triaryl-1,3,5-triazinanes might be used as Mannich reagents. On the other hand, all-carbon quaternary stereocenters are widely present in natural products and to build such structures is still a challenge, especially in a catalytic enantioselective manner.^10^ In recent years, our group has been committed to utilizing *N*,*N*′-dioxide–metal complexes as catalysts and has achieved a series of catalytic asymmetric reactions, including the construction of compounds with chiral all-carbon quaternary stereocenters.^11^ Herein, we report the first asymmetric Mannich reaction employing 1,3,5-triaryl-1,3,5-triazinanes as new Mannich reagents catalyzed by *N*,*N*′-dioxide–metal complexes, and a variety of optically active β-amino compounds, each with an all-carbon quaternary stereocenter, were obtained.

In our preliminary screening, the α-tetralone-derived β-keto ester **1a** and 1,3,5-triphenyl-1,3,5-triazinane **3a** were chosen as the model substrates to optimize the reaction conditions (Table 1). Initially, the performance of various metal salts was evaluated when combined with the chiral *N*,*N*′-dioxide ligand **L-PrPh**, which is derived from l-proline, and the reactions were performed in CH_2_Cl_2_ at 30 °C (Table 1, entries 1–5). Lanthanides, the *N*,*N*′-dioxide complexes of which have proved to be efficient catalysts for many reactions,^11^ can only provide the desired product **4a** with low ee values or as a racemate, although the yields were good (Table 1, entries 1–3). The complex of Mg(OTf)_2_ could give the desired product in 85% yield but with only 18% ee (Table 1, entry 4). To our delight, the complex of Ni(ClO_4_)_2_·6H_2_O provided **4a** with a better ee value (44% ee, Table 1, entry 5 *versus* entries 1–4). Increasing the steric hindrance of the amide substituents on the chiral *N*,*N*′-dioxide ligand further improved the enantioselectivity. Chiral *N*,*N*′-dioxide **L-PrPr_2_** with a more sterically hindered *i*-Pr at the *ortho*-positions of aniline improved the enantioselectivity to 53% ee (Table 1, entry 6 *versus* entry 5). Then we investigated the effect of the chiral backbone moiety, the (*S*)-pipecolic acid derived *N*,*N*′-dioxide **L-PiPr_2_** (Table 1, entry 8) was superior to l-proline derived **L-PrPr_2_** and l-ramipril-derived **L-RaPr_2_** (Table 1, entries 6 and 7), giving the product in 94% yield with 96% ee. In addition, lowering the temperature to 0 °C improved the enantioselectivity to 99% ee albeit with a lower yield (Table 1, entry 9). Remarkably, upon reducing the catalyst loading to 5 mol% the yield improved to 97% with the enantioselectivity maintained (Table 1, entry 10). When the α-tetralone-derived β-keto amide **2a** was employed in this reaction instead of **1a**, the desired product **5a** was obtained in good yield but with unsatisfactory enantioselectivity (Table 1, entry 11). Then we replaced the metal salt with Mg(OTf)_2_ and got comparable results (Table 1, entry 12).

**Table 1:** Optimization of the reaction conditions

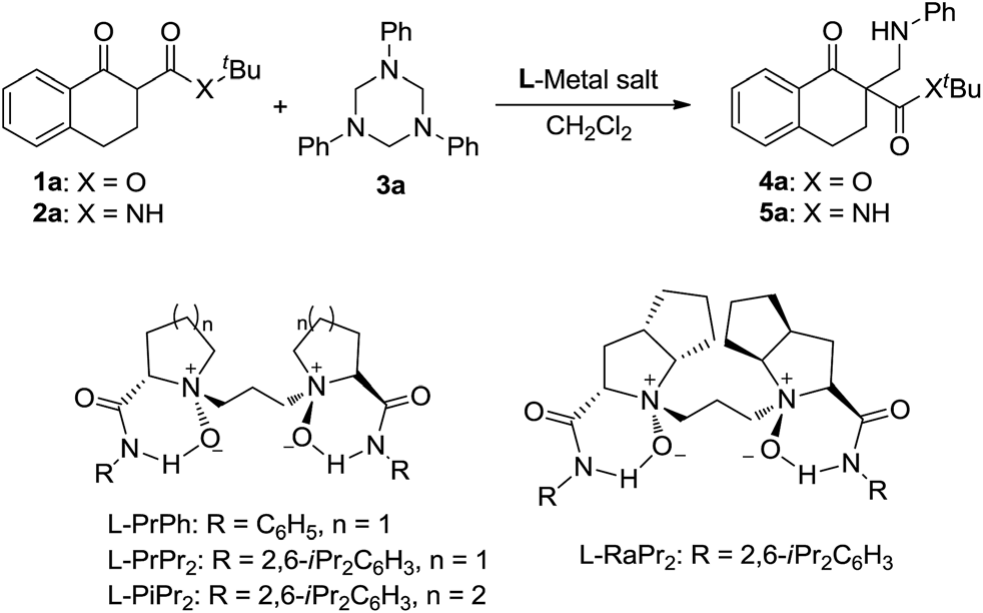					
Entry^*a*^	Substrate	Metal salt	Ligand	Yield^*b*^ (%)	ee^*c*^ (%)
1	**1a**	Sc(OTf)_3_	**L-PrPh**	83	0
2	**1a**	Yb(OTf)_3_	**L-PrPh**	84	0
3	**1a**	La(OTf)_3_	**L-PrPh**	90	13
4	**1a**	Mg(OTf)_2_	**L-PrPh**	85	18
5	**1a**	Ni(ClO_4_)_2_·6H_2_O	**L-PrPh**	97	44
6	**1a**	Ni(ClO_4_)_2_·6H_2_O	**L-PrPr_2_**	67	53
7	**1a**	Ni(ClO_4_)_2_·6H_2_O	**L-RaPr_2_**	87	87
8	**1a**	Ni(ClO_4_)_2_·6H_2_O	**L-PiPr_2_**	94	96
9^*d*^	**1a**	Ni(ClO_4_)_2_·6H_2_O	**L-PiPr_2_**	87	99
10^*d*^ ^,^ ^*e*^	**1a**	Ni(ClO_4_)_2_·6H_2_O	**L-PiPr_2_**	97	99
11^*d*^ ^,^ ^*e*^	**2a**	Ni(ClO_4_)_2_·6H_2_O	**L-PiPr_2_**	95	61
12^*d*^ ^,^ ^*f*^	**2a**	Mg(OTf)_2_	**L-PiPr_2_**	98	97

^*a*^Unless otherwise noted, the reactions were performed with **1a** or **2a** (0.10 mmol), **3a** (0.034 mmol), ligand (0.01 mmol), and metal salt (0.01 mmol) in 1.0 mL CH_2_Cl_2_ at 30 °C for 8 h.

^*b*^Isolated yield of the product.

^*c*^Determined by HPLC analysis on a chiral stationary phase.

^*d*^The reaction was performed at 0 °C for 12 h.

^*e*^5 mol% **L-PiPr_2_** (0.005 mmol) and 5 mol% Ni(ClO_4_)_2_·6H_2_O (0.005 mmol) were used.

^*f*^The reaction was performed with **L-PiPr_2_** (0.005 mmol) and Mg(OTf)_2_ (0.005 mmol).

With the optimized reaction conditions in hand, we firstly investigated the scope of the reactions between α-tetralone-derived β-keto esters and 1,3,5-triaryl-1,3,5-triazinanes (Table 2). Delightfully, the electronic nature and the positions of the substituents on the β-keto esters had little influence on both the yields and enantioselectivities (83–98% yield, 81–99% ee; **4a–4f**). Next, the 1,3,5-triaryl-1,3,5-triazinanes were varied. As it shown in Table 2 (**4g–4k**), the positions of the substituents have a certain influence on the yields, but the enantioselectivities were good in all cases. Generally, the 2-substituted 1,3,5-triaryl-1,3,5-triazinanes showed a slight decrease in yield compared with the 4-substituted ones. What's more, 1-adamantanol substituted β-keto ester **1l** was also a suitable substrate for this reaction and the corresponding product **4l** was obtained in 99% yield with 93% ee (Table 2, **4l**). Additionally, the absolute configuration of **4a** was determined to be *R* by X-ray crystallography^12^ and the configurations of the others were determined to be *R* by circular dichroism (for details see the ESI†).

**Table 2:** Substrate scope for β-keto esters^*a*^

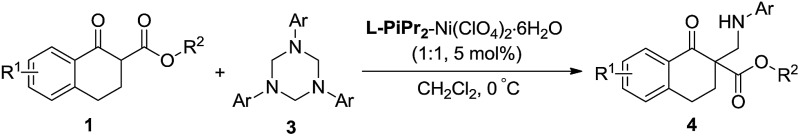
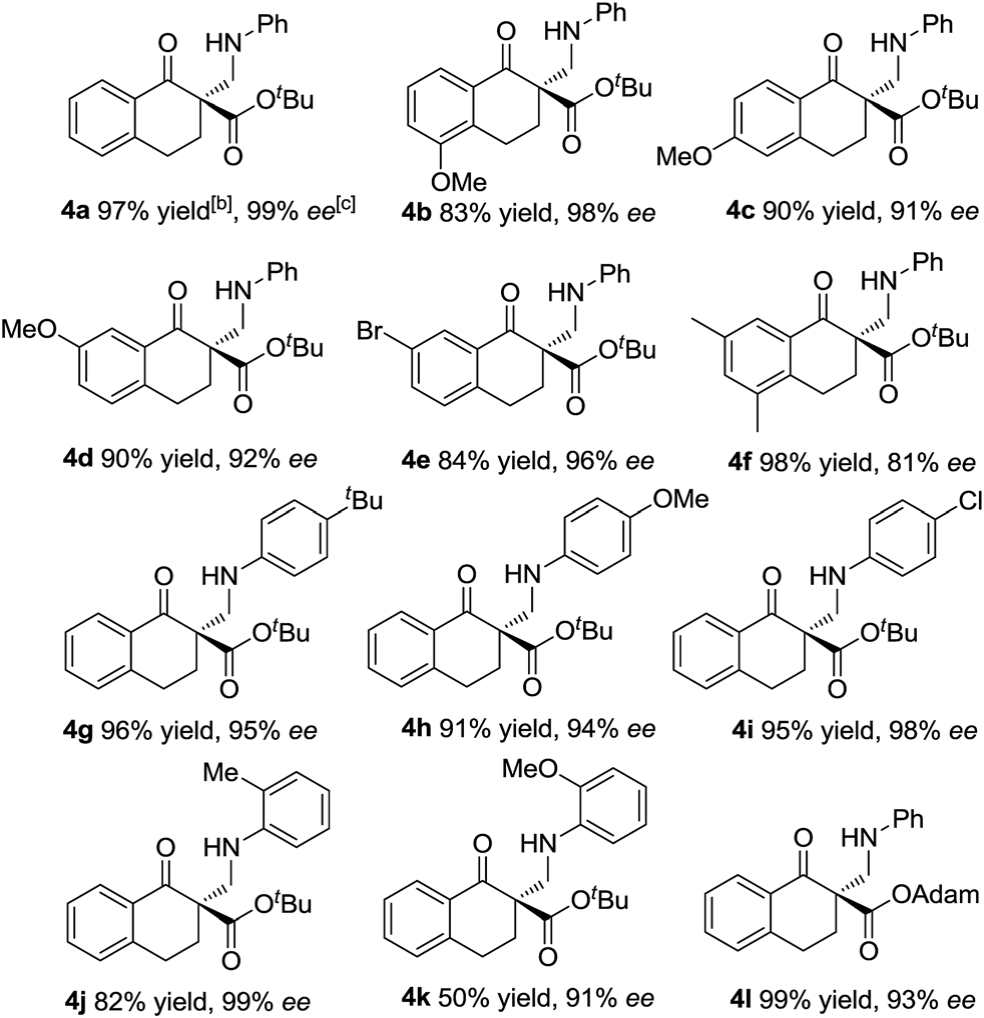

^*a*^The reactions were performed with **1** (0.10 mmol), **3** (0.034 mmol), **L-PiPr_2_** (0.005 mmol), and Ni(ClO_4_)_2_·6H_2_O (0.005 mmol) in 1.0 mL CH_2_Cl_2_ at 0 °C for 12 h.

^*b*^Isolated yield of the product.

^*c*^Determined by HPLC analysis on a chiral stationary phase.

Subsequently, we turned our attention to investigate the substrate scope of the reactions between α-tetralone-derived β-keto amides and 1,3,5-triaryl-1,3,5-triazinanes (Table 3). To our delight, a variety of β-keto amides with different substituents were tolerated and gave the corresponding products with excellent enantioselectivities (Table 3, 93–98% ee; **5a–5f**). Then the scope of 1,3,5-triaryl-1,3,5-triazinanes was examined. The results are different from the results for the reactions of the β-keto esters, and both 2- and 4-substituted 1,3,5-triaryl-1,3,5-triazinanes afforded the corresponding products in excellent yields and enantioselectivities (95–99% yields, 95–99% ee, **5g**, **5i** and **5j**) except the 4-MeO substituted 1,3,5-tris(4-methoxyphenyl)-1,3,5-triazinane, which gave the corresponding product in 84% ee. Besides this, five- and seven-membered β-keto amide substrates were also examined. Unfortunately, the five-membered β-keto amide gave the corresponding product **5k** with only 55% ee, while the seven-membered β-keto amide gave a racemic product **5l** though the yields were excellent under the standard conditions. A cyclohexanone-derived β-keto amide was also tested under the standard reaction conditions, but the reaction didn't occur. Meanwhile, the absolute configuration of **5a** was determined to be *R* by X-ray crystallography analysis^12^ and configurations of the others were also determined to be *R* by circular dichroism (for details see the ESI†).

**Table 3:** Substrate scope for β-keto amides^*a*^

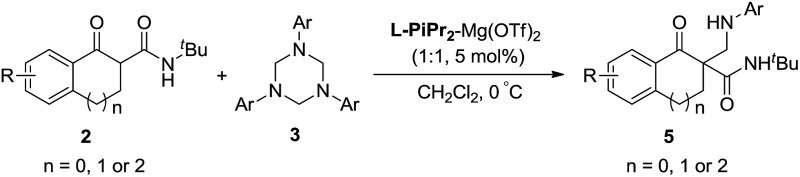
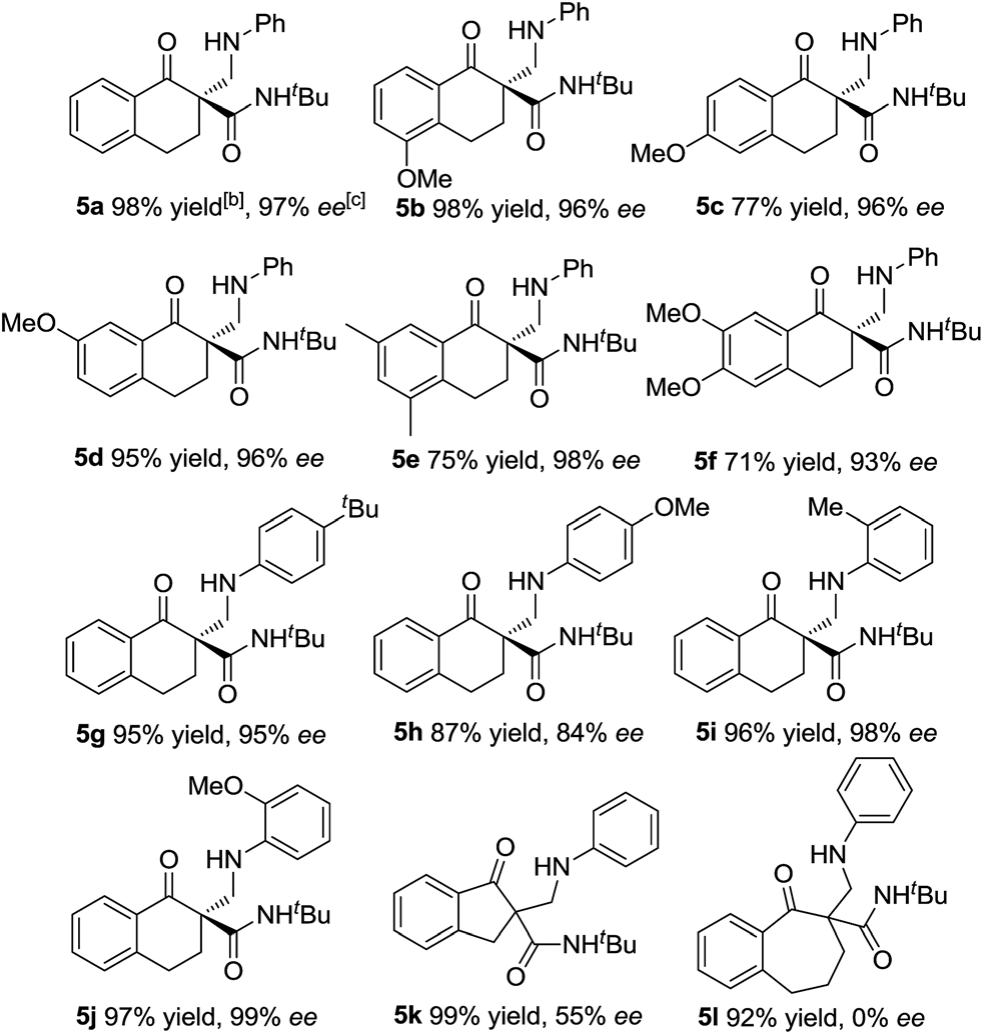

^*a*^The reactions were performed with **2** (0.10 mmol), **3** (0.034 mmol), **L-PiPr_2_** (0.005 mmol), and Mg(OTf)_2_ (0.005 mmol) in 1.0 mL CH_2_Cl_2_ at 0 °C for 12 h.

^*b*^Isolated yield of the product.

^*c*^Determined by HPLC analysis on a chiral stationary phase.

To evaluate the synthetic value of this catalytic system, gram-scale reactions were performed (Scheme 2). In the presence of the **L-PiPr_2_**–Ni(ClO_4_)_2_·6H_2_O complex (5 mol%), the starting material **1a** (4.0 mmol) reacted with **3a** (1.3 mmol, 1.0 equivalent) smoothly, and the corresponding product **4a** was obtained in 92% yield with 99% ee (Scheme 2a). In the system of α-tetralone-derived β-keto amides and 1,3,5-triaryl-1,3,5-triazinanes, the reaction between 0.98 g **2a** and 0.42 g **3a** was performed under the optimized reaction conditions, affording 1.34 g (95% yield) of the corresponding product **5a** with 97% ee (Scheme 2b).

**Scheme 2:**
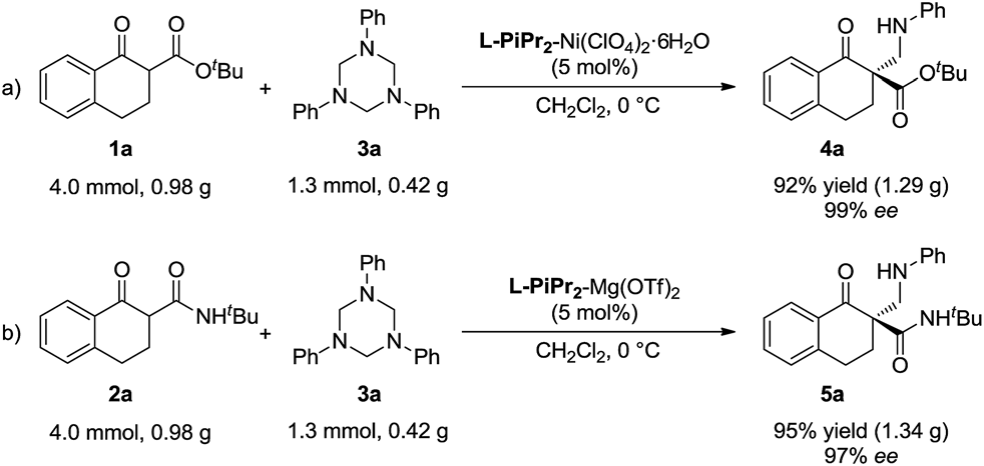
Gram-scale version of the reaction.

On the other hand, the product **4a** could be efficiently converted into useful β-hydroxyl ester **6** through reduction using NaBH_4_ as a reducing agent (Scheme 3). The diastereomer of the product **6** was determined to be *trans*- using NOESY spectra (see the ESI† for details). The product **4h** could be converted into *N*-Boc-β-amino ester **7** by deprotection with cerium ammonium nitrate (CAN) followed by Boc protection of the amino group with Boc_2_O (see the ESI† for details).

**Scheme 3:**
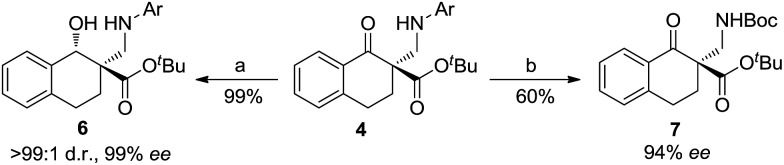
Transformations of the product **4** into other derivatives; reaction conditions: (a) NaBH_4_ and MeOH/CH_2_Cl_2_ (1 : 1), 0 °C (**4a**: Ar = Ph, 99% ee); (b) CAN, CH_3_CN/H_2_O; then Et_3_N and Boc_2_O (**4h**: Ar = 4-MeOC_6_H_4_, 94% ee). Boc = *tert*-butyloxycarbonyl.

To gain insight into the mechanism, the relationship between the ee value of the ligand **L-PiPr_2_** and that of **4a** was investigated under the optimal reaction conditions.^13^ A linear effect was observed (see the ESI† for details), which suggested that a monomeric catalyst may be the main catalytically active species in the reaction system. Based on the experiments and our previous work^11^ as well as the absolute configuration of the products, a possible transition state model is proposed in Fig. 1 to elucidate the origin of the asymmetric induction. In the transition state, the oxygens of the *N*,*N*′-dioxides and the amide oxygens coordinate to Ni(ii) in a tetradentate manner. The β-keto ester **1a** could be activated after coordinating to the nickel atom in a bidentate fashion. The *Si*-face of β-keto ester **1a** is effectively shielded by the amide moiety and the piperidine ring on the underside of the ligand **L-PiPr_2_**. In contrast, the *Re*-face is located in a relatively open space. The highly selective approach of the *in situ* generated *N*-methyleneaniline toward the *Re*-face of the bidentate-coordinated β-keto ester leads to the desired product with an *R* configuration, which is consistent with the observed absolute configuration of the product.

**Fig. 1:**
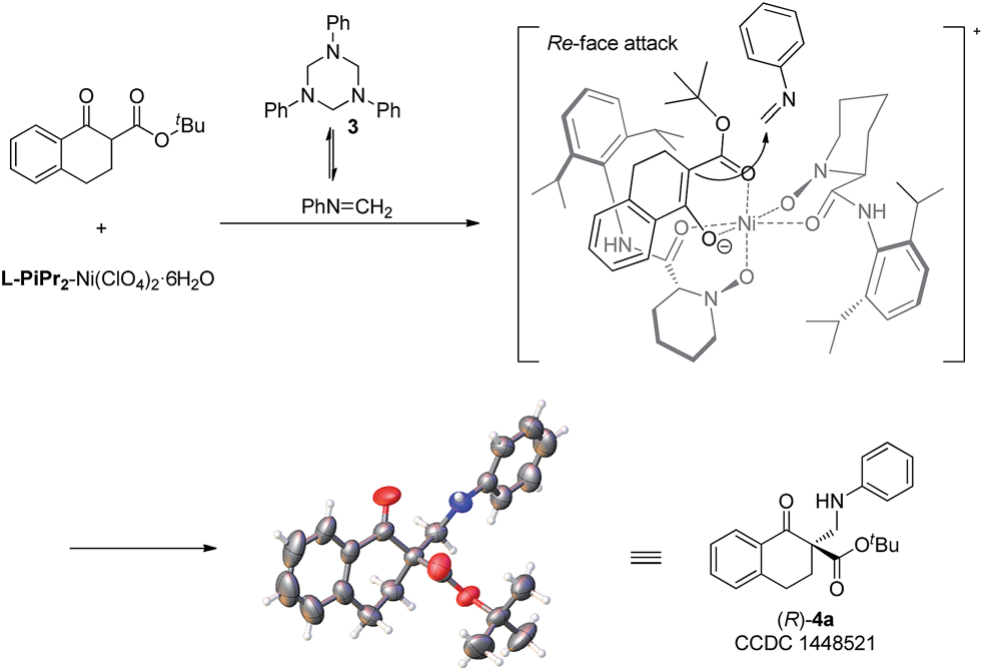
Proposed transition state and the absolute configuration of **4a**.

## Conclusions

In summary, a highly enantioselective Mannich-type reaction between α-tetralone-derived β-keto esters/amides and 1,3,5-triaryl-1,3,5-triazinanes was realized. In the presence of chiral *N*,*N*′-dioxide–Ni(ii) or *N*,*N*′-dioxide–Mg(ii) complex, a variety of corresponding β-amino compounds each with an all-carbon quaternary stereocenter were obtained in good to excellent enantioselectivities (up to 99% ee) and good to excellent yields (up to 99%). In particular, this is the first time that 1,3,5-triaryl-1,3,5-triazinanes were used as electrophilic reagents in the catalytic asymmetric Mannich reaction. Further studies focused on the reactions of 1,3,5-triaryl-1,3,5-triazinanes are under way.
