# Supplementary material for: A new approach to the asymmetric Mannich reaction catalyzed by chiral N,N′-dioxide–metal complexes
Source: Chem Sci. 2016 Oct 3;8(2):1238–42. doi: 10.1039/c6sc03902b (PMC5369522; doi:10.1039/c6sc03902b)

Supporting Information

Contents:

1. General information.. 2

2. The X-ray data for **4a** and **5a**... 2

3. The nonlinear effect between the ee value of the ligand **L-PiPr$_2$** and the product **4a**.. 5

4. Typical experimental procedure for the reduction of **4a**...................................... 5

5. Typical experimental procedure for the deprotection of **4h**.................................. 6

6. Reference...6

7. Characterization of the products.. 7

8. Copies of NMR spectra for the reaction products...31

9. The NOESY spectra of **6**..58

10. CD information of the products.. 59

1. General information

^1H NMR spectra were recorded on commercial instruments (400 MHz). Chemical shifts were recorded in ppm relative to tetramethylsilane and with the solvent resonance as the internal standard. Data were reported as follows: chemical shift, multiplicity (s = singlet, d = doublet, t = triplet, q = quartet, m = multiplet), coupling constants (Hz), integration. ^{13}C NMR data were collected on commercial instruments (100 MHz) with complete proton decoupling. Chemical shifts are reported in ppm from the tetramethylsilane with the solvent resonance as internal standard. Enantiomeric excesses (*ee*) were determined by chiral HPLC analysis on Daicel Chiralpak IC or IA in comparison with the authentic racemates. Optical rotations were reported as follows: $[\alpha]_D^T$ (c: g/100 mL, in solvent). HRMS was recorded on a commercial apparatus (ESI Source). The $Ni(ClO_4)_2{\cdot}6H_2O$, $Mg(OTf)_2$ is commercially available, and used without further purification. The CH_2Cl_2 was purified by usual methods before use. The α-tetralone-derived β-keto esters **1** and amides **2** were prepared by previously reported methods.[1] 1,3,5-triaryl-1,3,5-triazinanes **3** were prepared according to reported methods.[2] The *N,N'*-dioxide ligands **L** were synthesized according to the method reported by our group.[3]

2. The X-ray data for 4a and 5a.

(1) The X-ray data for 4a

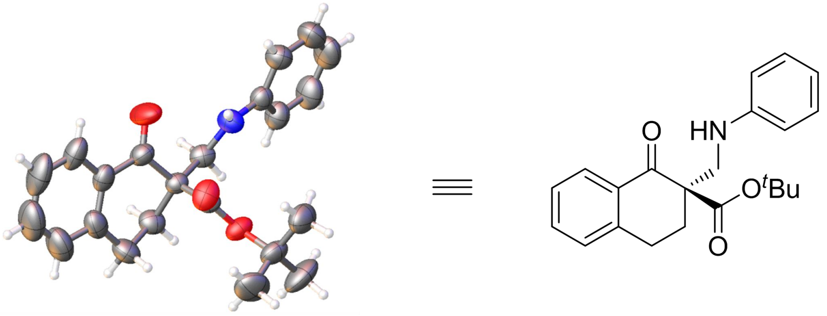

Single crystal of **4a** [$C_{22}H_{25}NO_3$] was obtained from the mixed solvents of ethyl acetate and petroleum ether. The absolute configuration is *R*. Mp 99–100 °C; $[\alpha]_D^{19}$ = 109.1 (c = 0.54, CH_2Cl_2). CCDC 1448521 contains the supplementary crystallographic data for this paper. These data can be obtained free of charge from the Cambrige Crystallographic Data Centere via www.ccdc.cam.ac.uk/data_request/cif.

Table 1 Crystal data and structure refinement for fxm-lxj.

Identification code	fxm-lxj
Empirical formula	$C_{22}H_{25}NO_3$

Formula weight	351.43
Temperature/K	217.05(10)
Crystal system	orthorhombic
Space group	$P2_12_12_1$
a/Å	6.05221(8)
b/Å	17.0223(3)
c/Å	19.5981(3)
α/°	90
β/°	90
γ/°	90
Volume/Å^3	2019.05(6)
Z	4
ρcalcg/cm^3	1.156
μ/mm^{-1}	0.610
F(000)	752.0
Crystal size/mm^3	0.7 × 0.2 × 0.2
Radiation	CuKα (λ = 1.54184)
2Θ range for data collection/°	9.024 to 134.104
Index ranges	$-7 \leq h \leq 4$, $-20 \leq k \leq 18$, $-19 \leq l \leq 23$
Reflections collected	10704
Independent reflections	3602 [R_{int} = 0.0285, R_{sigma} = 0.0203]
Data/restraints/parameters	3602/0/238
Goodness-of-fit on F^2	1.045
Final R indexes [I>=2σ (I)]	R_1 = 0.0524, wR_2 = 0.1425
Final R indexes [all data]	R_1 = 0.0546, wR_2 = 0.1470
Largest diff. peak/hole / e Å^{-3}	0.24/-0.26
Flack parameter	0.05(9)

(2) The X-ray data for 5a

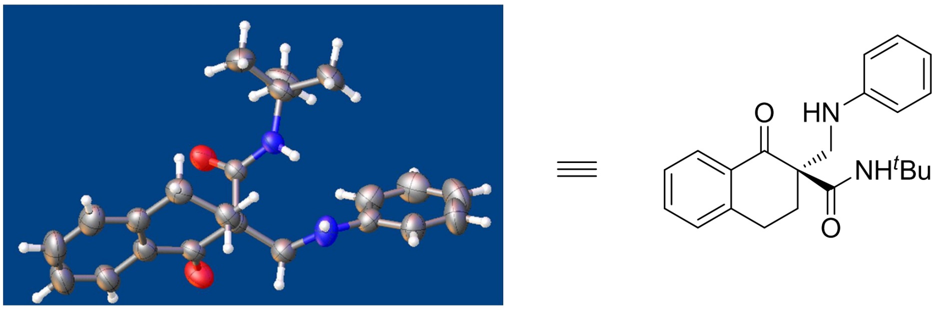

Single crystal of **5a** [$C_{22}H_{26}N_2O_2$] was obtained from the mixed solvents of ethyl acetate and petroleum ether. The absolute configuration is *R*. Mp 78–80 °C; $[\alpha]_D^{23}$ = 77.7 (c = 0.69, CH_2Cl_2). CCDC 1480808 contains the supplementary crystallographic data for this paper. These data can be obtained free of charge from

the Cambrige Crystallographic Data Centere via www.ccdc.cam.ac.uk/data_request/cif.

Table 2 Crystal data and structure refinement for fxm-lxj-nh-ph-5.

Identification code	fxm-lxj-nh-ph-5
Empirical formula	$C_{44}H_{52}N_4O_4$
Formula weight	700.89
Temperature/K	295.1(5)
Crystal system	monoclinic
Space group	$P2_1$
a/Å	9.20362(17)
b/Å	10.9688(3)
c/Å	19.3712(4)
α/°	90
β/°	100.345(2)
γ/°	90
Volume/Å^3	1923.78(7)
Z	2
ρcalcg/cm^3	1.210
μ/mm^{-1}	0.613
F(000)	752.0
Crystal size/mm^3	0.6 × 0.3 × 0.15
Radiation	CuKα (λ = 1.54184)
2Θ range for data collection/°	9.282 to 145.172
Index ranges	$-11 \leq h \leq 11$, $-13 \leq k \leq 12$, $-23 \leq l \leq 23$
Reflections collected	18988
Independent reflections	6766 [R_{int} = 0.0289, R_{sigma} = 0.0263]
Data/restraints/parameters	6766/1/475
Goodness-of-fit on F^2	1.043
Final R indexes [I>=2σ (I)]	R_1 = 0.0532, wR_2 = 0.1487
Final R indexes [all data]	R_1 = 0.0558, wR_2 = 0.1522
Largest diff. peak/hole / e Å^{-3}	0.24/-0.23
Flack parameter	0.04(12)

3. The nonlinear effect[4] between the *ee* value of the ligand L-PiPr₂ and the product 4a

To a dry reaction tube, the ligand (*S*)-**L-PiPr$_2$** (*x* mol% loading), (*R*)-**L-PiPr$_2$** (*y* mol% loading), $Ni(ClO_4)_2·6H_2O$ (0.005 mmol, 1.8 mg), **1a** (0.1 mmol) and CH_2Cl_2 (1.0 mL) were added and stirred at 30 °C for 0.5 h. Then **3a** (0.034 mmol) were added, and the reaction was stirred at 0 °C for 8 h. The product **4a** was purified by flash chromatography on silica gel (petroleum ether/ethyl acetate = 10/1).

x/*y* (mol%)	*ee* of **L-PiPr$_2$** [%]	*ee* of **4a** [%]
0.25/0.25	0	0
0.30/0.20	20	21
0.35/0.15	40	40
0.40/0.10	60	54
0.45/0.05	80	80
0.50/0	100	99

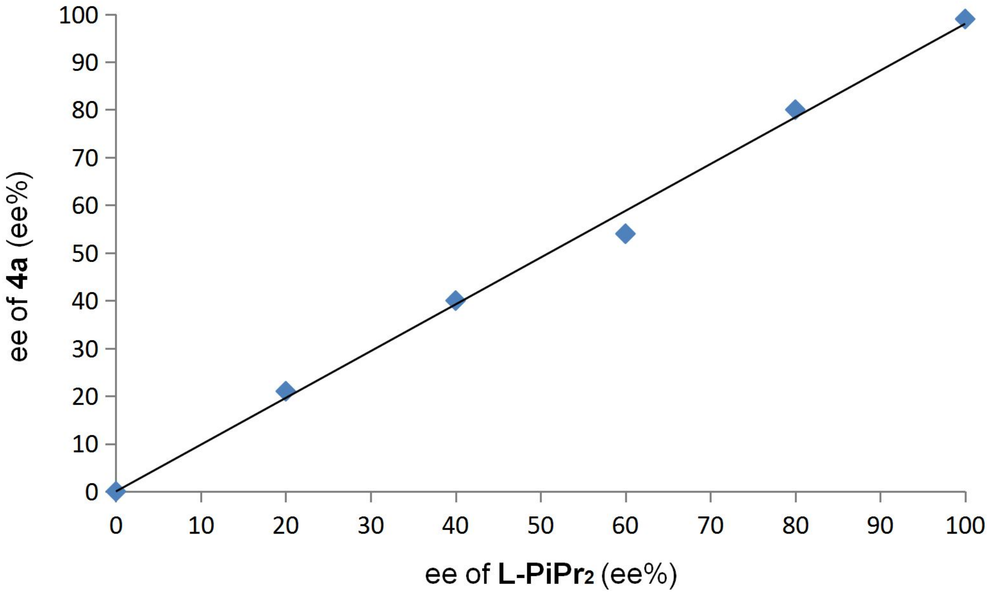

4. Typical experimental procedure for the reduction of 4a

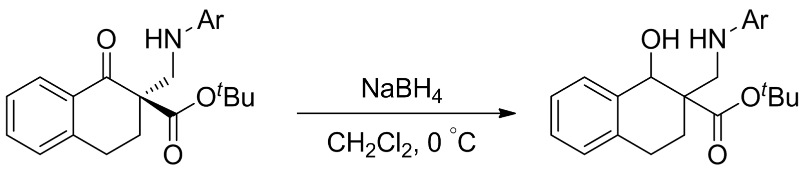

4a **6**

$NaBH_4$ (8.4 mg, 0.22 mmol) was added to a solution of **4a** (70.3 mg, 0.2 mmol)

in 2 mL MeOH/CH_2Cl_2 (1:1) at 0 °C, and the mixture was stirred at 0 °C, and monitored by TLC. After 1 h, the mixture was quenched by saturated NH_4Cl aq. and extraction with ethyl acetate three times (10 mL), and the solvent was removed in vacuo. The residue was purified by column chromatography on silica gel (petroleum ether: ethyl acetate = 10:1) to afford **6** (70.0 mg, 99% yield) as a white solid.

5. Typical experimental procedure for the deprotection of 4h

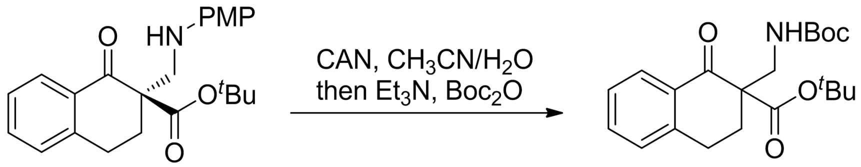

4h **7**

[$Ce(NO_3)_6(NH_4)_2$] (CAN; 274.1 mg, 0.5 mmol) in H_2O (2.0 mL) was added to a solution of **4h** (38.1 mg, 0.1 mmol) in CH_3CN (2.5 mL) at 0 °C. The solution was stirred at 0 °C. After 8 h, Et_3N (83.0 μL, 0.6 mmol) and Boc_2O (252.0 mg, 1.2 mmol) were added and the solution was stirred for another 18 h at 30 °C. Then saturated $NaHCO_3$ solution (3 mL) was added to the mixture and extracted three times with ethyl acetate (10 mL), and the combined organic phases were dried over $MgSO_4$, filtered, and the solvent was removed in vacuo. The residue was purified by column chromatography on silica gel (petroleum ether ethyl acetate = 10:1) to afford **7** (22.4 mg, 60% yield) as a yellow oil.

6. Reference

[1] C. Pan, X. Zeng, Y. Guan, X. Jiang, L. Li, H. Zhang, *Synlett.* **2011**, *3*, 425-429.

[2] *a*) C. A. Bischoff, F. Reinfeld, *Chem. Ber.* **1903**, *36*, 41-53; *b*) A. G. Giumanini, G. Verardo, E. Zangrando, L. Lassiani, *J. Prakt. Chem.* **1987**, *329*, 1087-1103; *c*) A. G. Giumanini, N. Toniutti, G. Verardo, M. Merli, *Eur. J. Org. Chem.* **1999**, 141-143; *d*) G. O. Jones, J. M. García, H. W. Horn, J. L. Hedrick, *Org. Lett.* **2014**, *16*, 5502-5505.

[3] *a*) Y. H. Wen, X. Huang, J. L. Huang, Y. Xiong, B. Qin, X. M. Feng, *Synlett* **2005**, 2445-2448; *b*) Z. P. Yu, X. H. Liu, Z. H. Dong, M. S. Xie, X. M. Feng, *Angew. Chem. Int. Ed.* **2008**, *47*, 1308-1311; *c*) X. Zhou, D. J. Shang, Q. Zhang, L. L. Lin, X. H. Liu, X. M. Feng, *Org. Lett.* **2009**, *11*, 1401-1404.

[4] *a*) C. Girard, H. B. Kagan, *Angew. Chem. Int. Ed.* **1998**, *37*, 2922-2959; *b*) H. B. Kagan, *Adv. Synth. Catal.* **2001**, *343*, 227-233; *c*) T. Satyanarayana, S. Abraham, H. B. Kagan, *Angew. Chem. Int. Ed.* **2009**, *48*, 456-494.

7. Characterization of the products

tert-butyl 1-oxo-2-((phenylamino)methyl)-1,2,3,4-tetrahydronaphthalene-2-carboxylate (**4a**): Purified by flash chromatography (petroleum ether: EtOAc = 10:1) to afford a white solid in 97% yield, 99% ee; mp 99–100 °C; $[\alpha]_D^{19}$ = 109.1 (c = 0.54, CH_2Cl_2). HPLC (Chiralpak IC, hexane/*i*-PrOH = 80:20, flow rate 1.0 mL/min, λ = 254 nm) retention time: 8.26 min (minor), 10.19 min (major). ^{1}H NMR (400 MHz, $CDCl_3$): δ 7.99–7.97 (dd, *J* = 7.6, 0.8 Hz, 1H), 7.42–7.38 (m, 1H), 7.27–7.23 (t, *J* = 7.6 Hz, 1H), 7.15–7.13 (d, *J* = 8.0 Hz, 1H), 7.10–7.06 (t, *J* = 8.0 Hz, 2H), 6.60–6.58 (m, 3H), 4.56 (s, 1H), 3.63–3.60 (d, *J* = 13.2 Hz, 1H), 3.47–3.44 (d, *J* = 13.2 Hz, 1H), 3.05–3.00 (m, 1H), 2.98–2.83 (m, 1H), 2.46–2.41 (m, 1H), 2.21–2.13 (m, 1H), 1.20 (s, 9H) ppm. ^{13}C NMR (100 MHz, $CDCl_3$): δ 196.8, 170.4, 148.4, 142.8, 133.5, 132.6, 129.2, 128.7, 127.6, 126.8, 117.4, 113.2, 82.7, 59.4, 48.3, 30.8, 27.7, 26.2 ppm. HRMS (ESI-TOF) calcd for $C_{22}H_{25}NNaO_3$ ([M+Na$^+$]) = 374.1732, Found 374.1731.

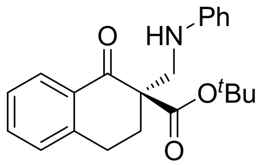

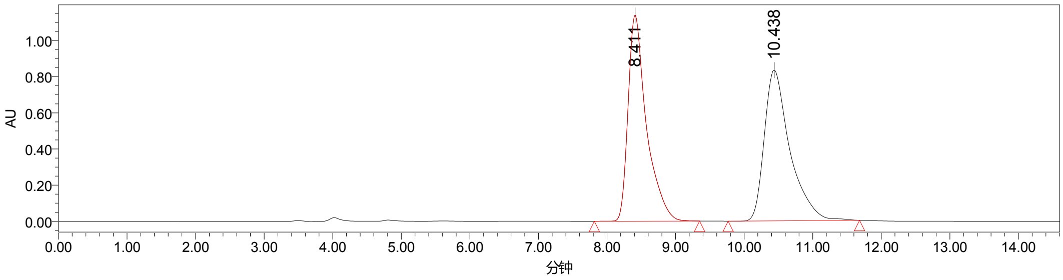

	Retention Time	% Area
1	8.411	49.54
2	10.438	50.46

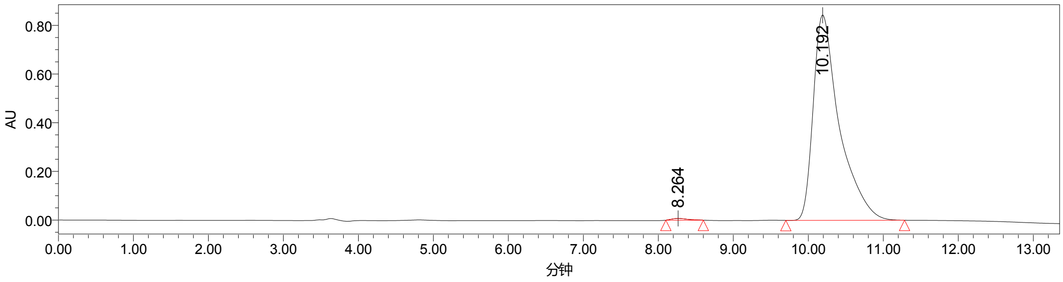

	Retention Time	% Area
1	8.264	0.48
2	10.192	99.52

tert-butyl 5-methoxy-1-oxo-2-((phenylamino)methyl)-1,2,3,4-tetrahydronaphthalene-2-carboxylate (**4b**): Purified by flash chromatography (petroleum ether: EtOAc = 10:1) to afford a white solid in 83% yield, 98% ee; mp 117–119 °C; $[\alpha]_D^{19}$ = 121.7 (c = 0.63, CH_2Cl_2). HPLC (Chiralpak IC, hexane/*i*-PrOH = 90:10, flow rate 1.0 mL/min, λ = 254 nm) retention time: 10.81 min (minor), 16.93 min (major). ^{1}H NMR (400 MHz, $CDCl_3$): δ 7.60–7.58 (d, J = 8.0 Hz, 1H), 7.24–7.20 (t, J = 8.0 Hz, 1H), 7.09–7.05 (t, J = 7.8 Hz, 2H), 6.95–6.93 (d, J = 8.0 Hz, 1H), 6.61–6.58 (m, 3H), 4.55 (s, 1H), 3.77 (s, 3H), 3.61–3.58 (d, J = 12.8 Hz, 1H), 3.46–3.43 (d, J = 12.8 Hz, 1H), 3.02–2.95 (m, 1H), 2.73–2.64 (m, 1H), 2.48–2.42 (m, 1H), 2.12–2.04 (m, 1H), 1.19 (s, 9H) ppm. ^{13}C NMR (100 MHz, $CDCl_3$): δ 197.2, 170.4, 156.6, 148.5, 133.7, 131.8, 129.2, 127.1, 119.1, 117.4, 114.2, 113.2, 82.6, 58.9, 55.7, 48.2, 30.1, 27.7, 20.1 ppm. HRMS (ESI-TOF) calcd for $C_{23}H_{27}NNaO_4$ ($[M+Na^+]$) = 404.1838, Found 404.1837.

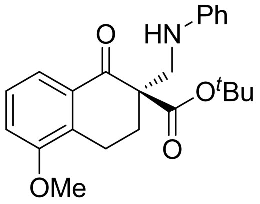

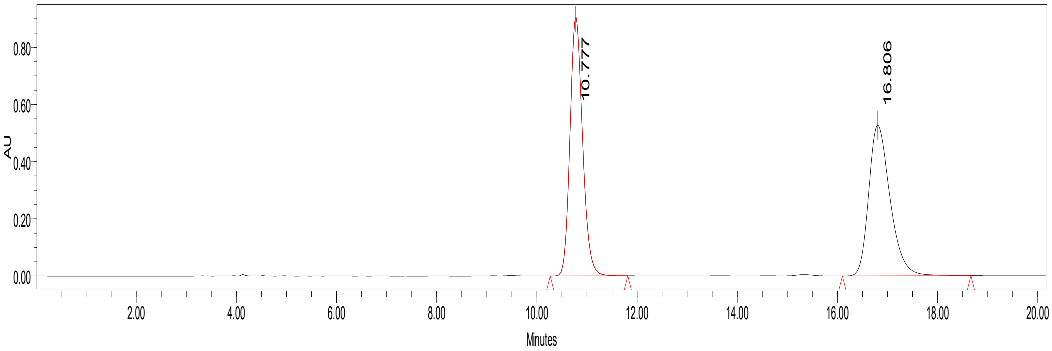

	Retention Time	% Area
1	10.777	50.30
2	16.806	49.70

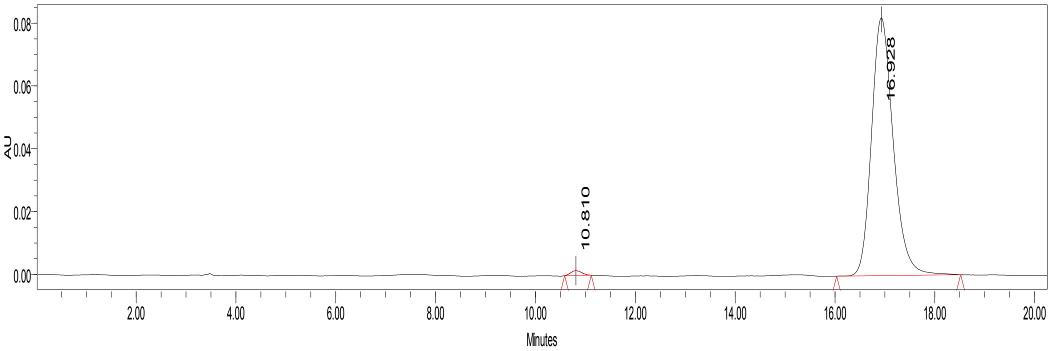

	Retention Time	% Area
1	10.810	0.94
2	16.928	99.06

tert-butyl 6-methoxy-1-oxo-2-((phenylamino)methyl)-1,2,3,4-tetrahydronaphthalene -2-carboxylate (**4c**): Purified by flash chromatography (petroleum ether: EtOAc = 10:1) to afford a white solid in 90% yield, 91% ee; mp 79–80 °C; $[\alpha]_D^{19}$ = 108.0 (c = 0.68, CH_2Cl_2). HPLC (Chiralpak IC, hexane/*i*-PrOH = 95:5, flow rate 1.0 mL/min, λ = 254 nm) retention time: 12.56 min (minor), 13.25 min (major). ^1H NMR (400 MHz, $CDCl_3$): δ 7.97–7.95 (d, *J* = 8.8 Hz, 1H), 7.09–7.05 (t, *J* = 8.0 Hz, 2H), 6.78–6.75 (dd, *J* = 8.8, 2.8 Hz, 1H), 6.59–6.57 (m, 4H), 4.59 (s, 1H), 3.77 (s, 3H), 3.62–3.58 (d, *J* = 8.8 Hz, 1H), 3.45–3.42 (d, *J* = 8.8 Hz, 1H), 3.01–2.93 (m, 1H), 2.84–2.78 (m, 1H), 2.43–2.37 (m, 1H), 2.19–2.11 (m, 1H), 1.23 (s, 9H) ppm. ^{13}C NMR (100 MHz, $CDCl_3$): δ 195.4, 170.6, 163.7, 148.5, 145.4, 130.1, 129.2, 126.1, 117.3, 113.5, 113.2, 112.4, 82.5, 59.1, 55.5, 48.3, 30.9, 27.8, 26.6 ppm. HRMS (ESI-TOF) calcd for $C_{23}H_{27}NNaO_4$ ($[M+Na^+]$) = 404.1838, Found 404.1837.

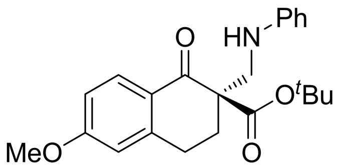

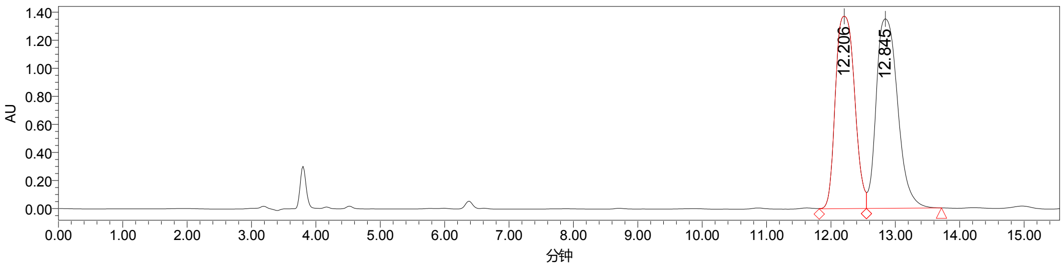

	Retention Time	% Area
1	12.206	47.51
2	12.845	52.49

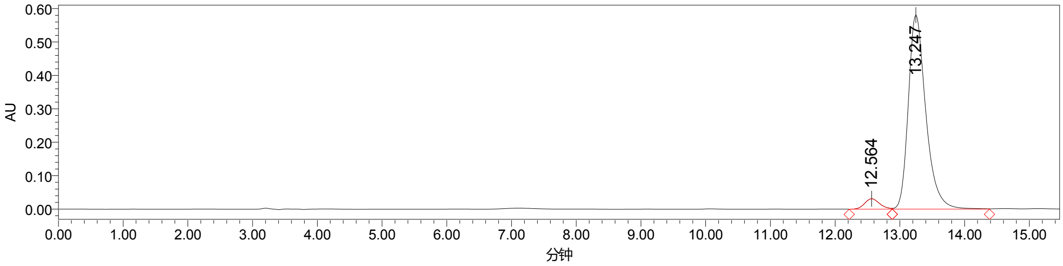

	Retention Time	% Area
1	12.564	4.39
2	13.247	95.61

tert-butyl 7-methoxy-1-oxo-2-((phenylamino)methyl)-1,2,3,4-tetrahydronaphthalene -2-carboxylate (**4d**): Purified by flash chromatography (petroleum ether: EtOAc = 10:1) to afford a white solid in 90% yield, 92% ee; mp 78–80 °C; $[\alpha]_D^{19}$ = 127.6 (c = 0.68, CH_2Cl_2). HPLC (Chiralpak IC, hexane/*i*-PrOH = 90:10, flow rate 1.0 mL/min, λ = 254 nm) retention time:

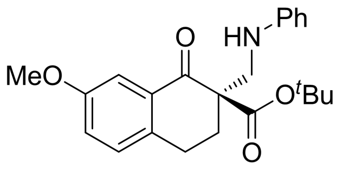

10.42 min (minor), 23.64 min (major). ^{1}H NMR (400 MHz, $CDCl_3$): δ 7.46–7.45 (d, J = 2.8 Hz, 1H), 7.10–7.04 (m, 3H), 7.00–6.97 (m, 1H), 6.62–6.58 (m, 3H), 4.55 (s, 1H), 3.77 (s, 3H), 3.63–3.60 (d, J = 12.8 Hz, 1H), 3.46–3.43 (d, J = 12.8 Hz, 1H), 2.97–2.89 (m, 1H), 2.83–2.77 (m, 1H), 2.44–2.39 (m, 1H), 2.19–2.11 (m, 1H), 1.22 (s, 9H) ppm. ^{13}C NMR (100 MHz, $CDCl_3$): δ 196.8, 170.5, 158.5, 148.4, 135.4, 133.3, 129.9, 129.2, 122.0, 117.4, 113.2, 109.4, 82.7, 59.3, 55.5, 48.3, 31.1, 27.7, 25.4 ppm. HRMS (ESI-TOF) calcd for $C_{23}H_{27}NNaO_4$ ([M+Na^{+}]) = 404.1838, Found 404.1834.

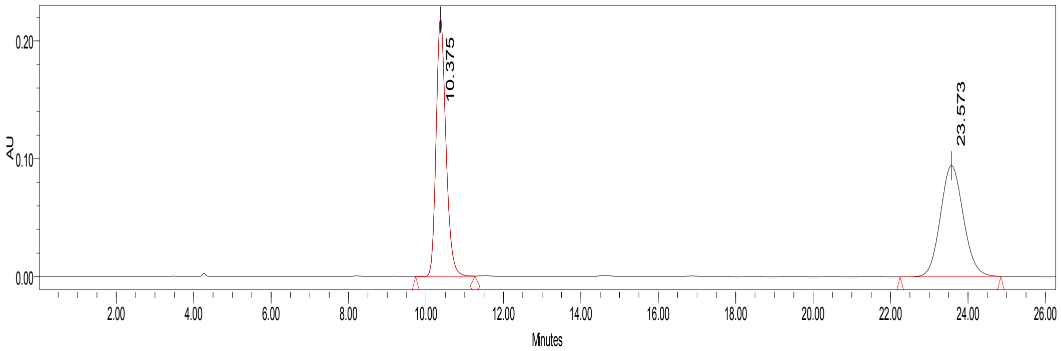

	Retention Time	% Area
1	10.375	49.91
2	23.573	50.09

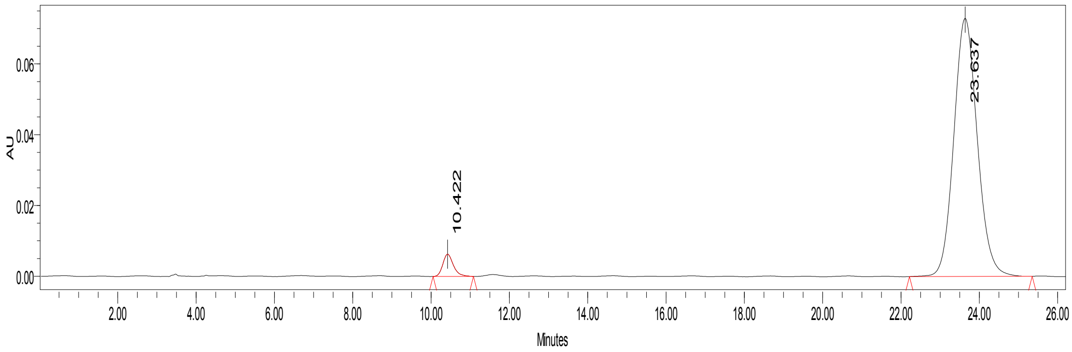

	Retention Time	% Area
1	10.422	3.76
2	23.637	96.24

tert-butyl 7-bromo-1-oxo-2-((phenylamino)methyl)-1,2,3,4-tetrahydronaphthalene -2-carboxylate (**4e**): Purified by flash chromatography (petroleum ether: EtOAc = 10:1) to afford a white solid in 84% yield, 96% ee; mp 109–110 °C; $[\alpha]_D^{19}$ = 98.5 (c = 0.71, CH_2Cl_2). HPLC (Chiralpak IC, hexane/*i*-PrOH = 90:10, flow rate 1.0 mL/min, λ = 254 nm) retention time: 6.96 min (minor), 10.19 min (major). ^{1}H NMR (400 MHz, $CDCl_3$): δ 8.10–8.09 (d, J = 2.0 Hz, 1H), 7.51–7.49 (m, 1H), 7.10–7.03 (m, 3H), 6.61–6.58 (m, 3H), 4.46 (s, 1H), 3.64–3.61 (d, J = 12.8 Hz, 1H), 3.47–3.43 (d, J = 12.8 Hz, 1H), 2.97–2.78 (m, 2H),

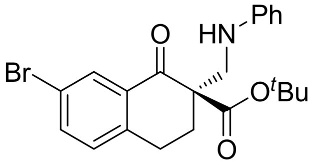

2.45–2.40 (m, 1H), 2.19–2.11 (m, 1H), 1.22 (s, 9H) ppm. ^{13}C NMR (100 MHz, $CDCl_3$): δ 195.5, 170.1, 148.3, 141.5, 136.3, 134.1, 130.5, 130.3, 129.2, 120.8, 117.6, 113.2, 83.1, 59.3, 48.1, 30.6, 27.8, 25.8 ppm. HRMS (ESI-TOF) calcd for $C_{22}H_{24}{}^{78.9183}BrNNaO_3$ ([M+Na$^+$]) = 452.0837, Found 452.0814. HRMS (ESI-TOF) calcd for $C_{22}H_{24}{}^{80.9163}BrNNaO_3$ ([M+Na$^+$]) = 454.0817, Found 454.0805.

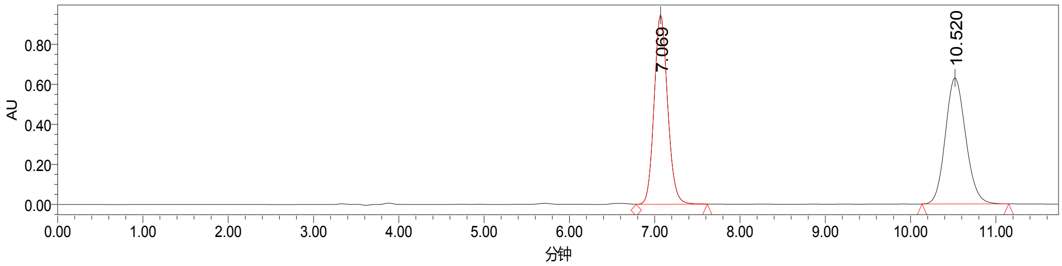

	Retention Time	% Area
1	7.069	49.85
2	10.520	50.15

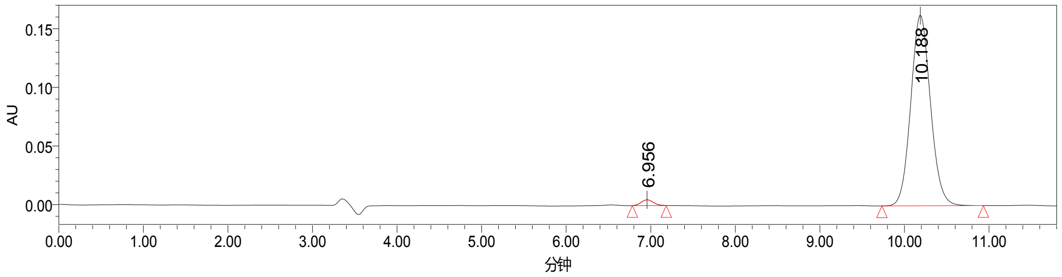

	Retention Time	% Area
1	6.956	1.82
2	10.188	98.18

tert-butyl 5,7-dimethyl-1-oxo-2-((phenylamino)methyl)-1,2,3,4-tetrahydronaphthal

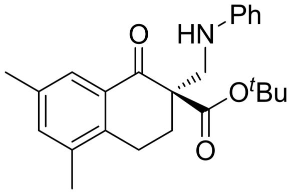

-ene-2-carboxylate (**4f**): Purified by flash chromatography (petroleum ether: EtOAc = 10:1) to afford a white solid in 98% yield, 81% ee; mp 138–140 °C; $[\alpha]_D^{19}$ = 86.8 (c = 0.74, CH_2Cl_2). HPLC (Chiralpak IC, hexane/*i*-PrOH = 90:10, flow rate 1.0 mL/min, λ = 254 nm) retention time: 10.16 min (minor), 29.41 min (major). ^{1}H NMR (400 MHz, $CDCl_3$): δ 7.66 (s, 1H), 7.11–7.05 (m, 3H), 6.60–6.58 (m, 1H), 4.57 (s, 1H), 3.61–3.57 (d, *J* = 12.8 Hz, 1H), 3.46–3.43 (d, *J* = 12.8 Hz, 1H), 2.82–2.67 (m, 1H), 2.48–2.43 (m, 1H), 2.26 (s, 3H), 2.16 (s, 3H) 2.13–2.07 (m, 1H), 1.19 (s, 9H) ppm. ^{13}C NMR (100 MHz, $CDCl_3$): δ 196.3, 169.4, 147.4, 137.1, 135.1, 135.0, 134.9, 131.6, 128.1, 124.5, 116.3, 112.1, 81.5, 57.7, 47.1, 29.1, 26.7, 22.2, 19.8, 18.2 ppm. HRMS (ESI-TOF) calcd for $C_{24}H_{29}NNaO_3$ ([M+Na$^+$]) = 402.2045, Found 402.2041.

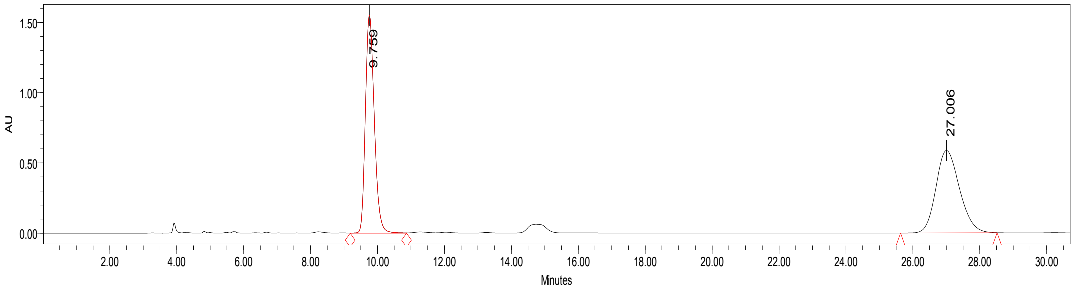

	Retention Time	% Area
1	9.759	49.94
2	27.006	50.06

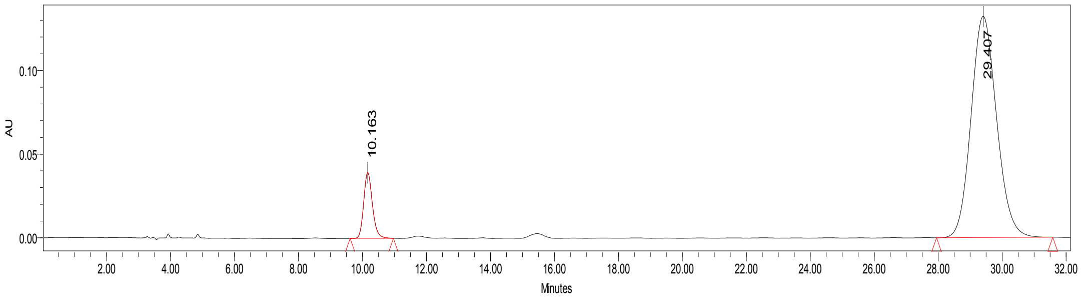

	Retention Time	% Area
1	10.163	9.22
2	29.407	90.78

tert-butyl 2-(((4-(tert-butyl)phenyl)amino)methyl)-1-oxo-1,2,3,4-tetrahydronaphthal

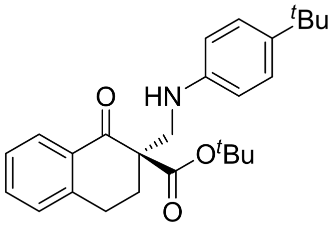

-ene-2-carboxylate (**4g**): Purified by flash chromatography (petroleum ether: EtOAc = 10:1) to afford a yellow oil in 96% yield, 95% ee; $[\alpha]_D^{21}$ = 85.3 (c = 0.78, CH_2Cl_2). HPLC (Chiralpak IC, hexane/*i*-PrOH = 90:10, flow rate 1.0 mL/min, λ = 254 nm) retention time: 7.90 min (minor), 14.62 min (major). ^{1}H NMR (400 MHz, $CDCl_3$): δ 7.99–7.97 (m, 1H), 7.41–7.37 (m, 1H), 7.26–7.22 (m, 1H), 7.17–7.10 (m, 3H), 6.56–6.55 (m, 2H), 4.38 (s, 1H), 3.62–3.58 (d, *J* = 12.4 Hz, 1H), 3.45–3.42 (d, *J* = 12.4 Hz, 1H), 3.05–2.98 (m, 1H), 2.89–2.84 (m, 1H), 2.46–2.42 (m, 1H), 2.23–2.16 (m, 1H), 1.21–1.19 (m, 18H) ppm. ^{13}C NMR (100 MHz, $CDCl_3$): δ 194.2, 168.0, 143.6, 140.3, 137.7, 130.9, 130.1, 126.1, 125.1, 124.2, 123.4, 110.5, 80.1, 56.8, 46.0, 31.3, 29.0, 28.3, 25.2, 23.6 ppm. HRMS (ESI-TOF) calcd for $C_{26}H_{33}NNaO_3$ ($[M+Na^+]$) = 430.2358, Found 430.2356.

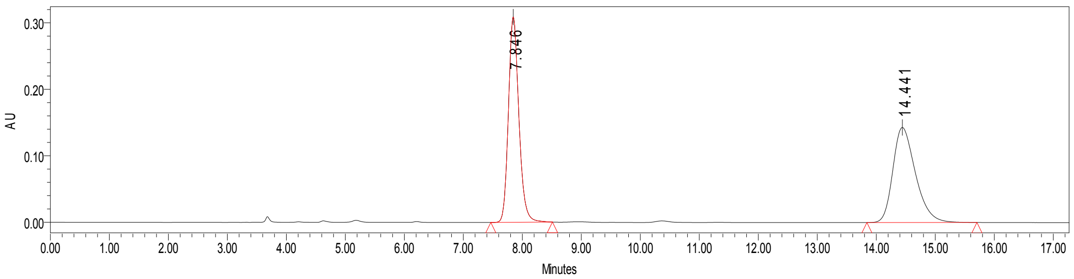

	Retention Time	% Area
1	7.846	50.00
2	14.441	50.00

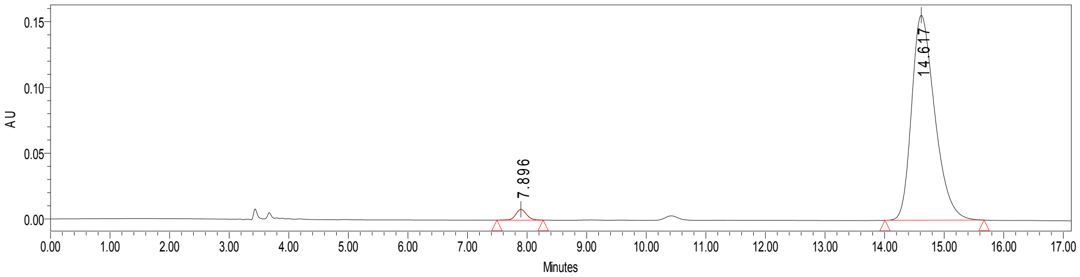

	Retention Time	% Area
1	7.896	2.39
2	14.617	97.61

tert-butyl 2-(((4-methoxyphenyl)amino)methyl)-1-oxo-1,2,3,4-tetrahydronaphthal-ene-2-carboxylate (**4h**): Purified by flash chromatography (petroleum ether: EtOAc = 10:1) to afford a white solid in 91% yield, 94% ee; mp 106–108 °C; $[\alpha]_D^{22}$ = 91.6 (c = 0.69, CH_2Cl_2). HPLC (Chiralpak IC, hexane/*i*-PrOH = 90:10, flow rate 1.0 mL/min, λ = 254 nm) retention time: 7.90 min (minor), 14.62 min (major). ^{1}H NMR (400 MHz, $CDCl_3$): δ 7.99–7.97 (m, 1H), 7.42–7.38 (m, 1H), 7.27–7.23 (m, 1H), 7.15–7.13 (m, 1H), 6.70–6.68 (m, 2H), 6.58–6.56 (m, 2H), 4.30 (s, 1H), 3.66 (s, 3H), 3.54–3.51 (d, *J* = 12.8 Hz, 1H), 3.42–3.39 (d, *J* = 12.8 Hz, 1H), 3.05–2.96 (m, 1H), 2.89–2.83 (m, 1H), 2.45–2.40 (m, 1H), 2.22–2.14 (m, 1H), 1.22 (s, 9H) ppm. ^{13}C NMR (100 MHz, $CDCl_3$): δ 196.9, 170.5, 152.1, 142.8, 133.5, 132.6, 128.7, 127.6, 126.8, 114.8, 114.7, 82.6, 59.3, 55.8, 49.6, 30.9, 27.8, 26.2 ppm. HRMS (ESI-TOF) calcd for $C_{23}H_{27}NNaO_4$ ([M+Na$^+$]) = 404.1838, Found 404.1836.

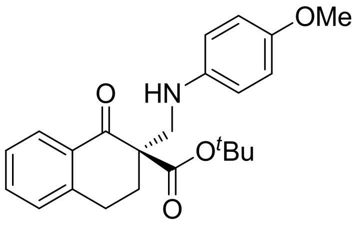

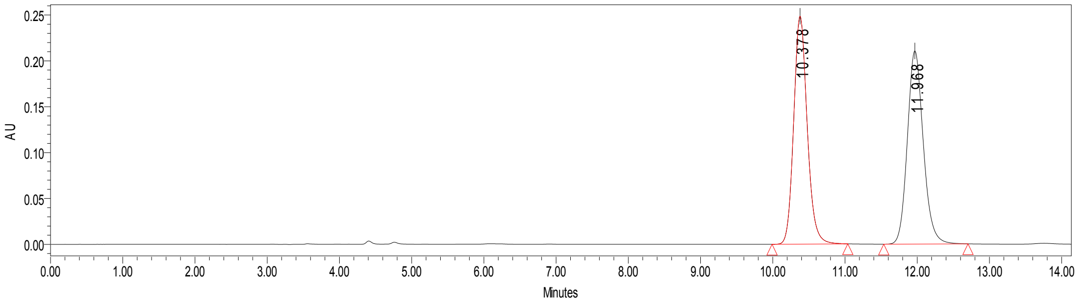

	Retention Time	% Area
1	10.378	49.99
2	11.968	50.01

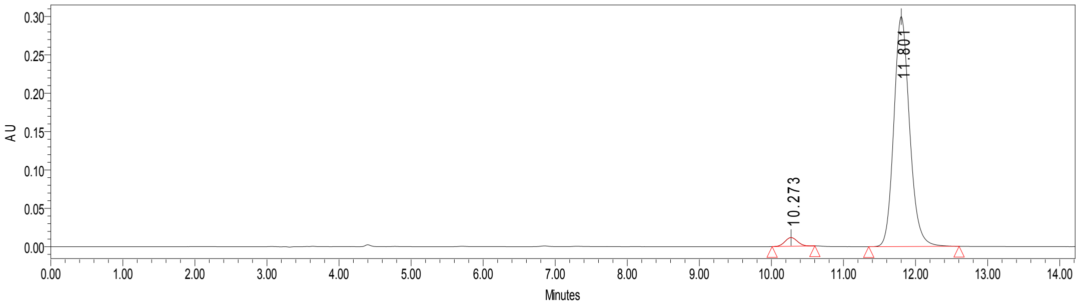

	Retention Time	% Area
1	10.273	2.92
2	11.801	97.08

tert-butyl 2-(((4-chlorophenyl)amino)methyl)-1-oxo-1,2,3,4-tetrahydronaphthalene-2-carboxylate (**4i**): Purified by flash chromatography (petroleum ether: EtOAc = 10:1) to afford a white solid in 95% yield, 98% ee; mp 138–140 °C; $[\alpha]_D^{22}$ = 108.9 (c = 0.73, CH_2Cl_2). HPLC (Chiralpak IA, hexane/*i*-PrOH = 90:10, flow rate 1.0 mL/min, λ = 254 nm) retention time: 6.56 min (minor), 7.41 min (major). ^{1}H NMR (400 MHz, $CDCl_3$): δ 7.98–7.96 (m, 1H), 7.43–7.39 (m, 1H), 7.27–7.24 (m, 1H), 7.16–7.14 (m, 1H), 7.02–7.00 (m, 2H), 6.52–6.50 (m, 2H), 4.63 (s, 1H), 3.57–3.54 (d, *J* = 12.8 Hz, 1H), 3.43–3.40 (d, *J* = 12.8 Hz, 1H), 3.05–2.96 (m, 1H), 2.90–2.83 (m, 1H), 2.43–2.38 (m, 1H), 2.16–2.09 (m, 1H), 1.19 (s, 9H) ppm. ^{13}C NMR (100 MHz, $CDCl_3$): δ 196.9, 170.3, 147.0, 142.7, 133.6, 132.6, 128.0, 129.70, 127.6, 126.9, 121.8, 114.2, 82.9, 59.2, 48.5, 30.9, 27.7, 26.2 ppm. HRMS (ESI-TOF) calcd for $C_{22}H_{24}{}^{34.9689}ClNNaO_3$ ([M+Na$^+$]) = 408.1342, Found 408.1341. HRMS (ESI-TOF) calcd for $C_{22}H_{24}{}^{36.9659}ClNNaO_3$ ([M+Na$^+$]) = 410.1313, Found 410.1289.

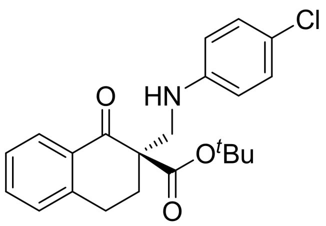

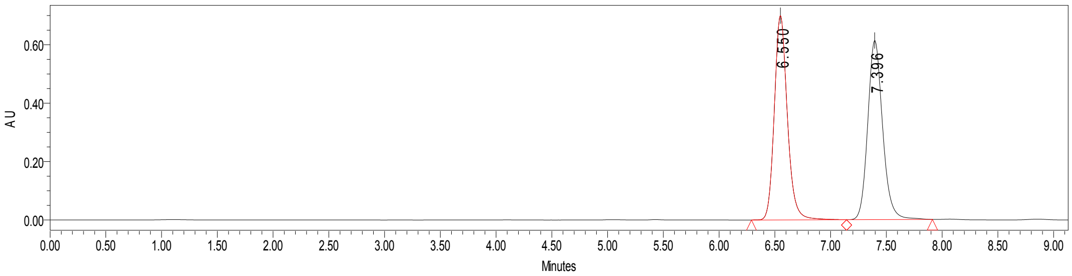

	Retention Time	% Area
1	6.550	50.00
2	7.396	50.00

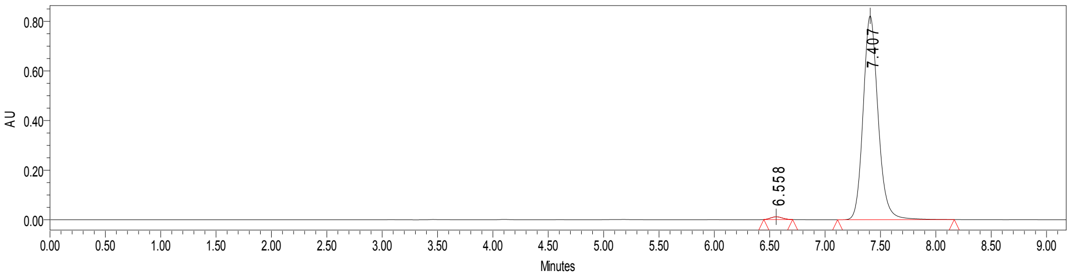

	Retention Time	% Area
1	6.558	1.04
2	7.407	98.96

tert-butyl 1-oxo-2-((o-tolylamino)methyl)-1,2,3,4-tetrahydronaphthalene-2-

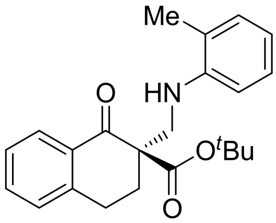

carboxylate (**4j**): Purified by flash chromatography (petroleum ether: EtOAc = 10:1) to afford a white solid in 82% yield, 99% ee; mp 84–86 °C; $[\alpha]_D^{19}$ = 107.3 (c = 0.60, CH_2Cl_2). HPLC (Chiralpak ID, hexane/*i*-PrOH = 90:10, flow rate 1.0 mL/min, λ = 254 nm) retention time: 6.11 min (minor), 7.54 min (major). ^{1}H NMR (400 MHz, $CDCl_3$): δ 8.00–7.98 (m, 1H), 7.42–7.38 (m, 1H), 7.27–7.23 (m, 1H), 7.16–7.14 (m, 1H), 7.05–7.01 (m, 1H), 6.97–6.95 (m, 1H), 6.63–6.61 (m, 1H), 6.58–6.54 (m, 1H), 4.58 (s, 1H), 3.62–3.59 (d, *J* = 12.4 Hz, 1H), 3.49–3.45 (d, *J* = 12.4 Hz, 1H), 3.06–2.97 (m, 1H), 2.90–2.83 (m, 1H), 2.50–2.43 (m, 1H), 2.18–2.17 (m, 1H), 2.09(s, 3H), 1.19 (s, 9H) ppm. ^{13}C NMR (100 MHz, $CDCl_3$): δ 197.0, 170.4, 146.5, 142.7, 133.5, 132.6, 130.1, 128.7, 127.6, 127.0, 126.8, 122.7, 117.0, 109.9, 82.7, 59.0, 48.3, 31.1, 27.7, 26.2, 17.6 ppm. HRMS (ESI-TOF) calcd for $C_{23}H_{27}NNaO_3$ ($[M+Na^+]$) = 388.1889, Found 388.1887.

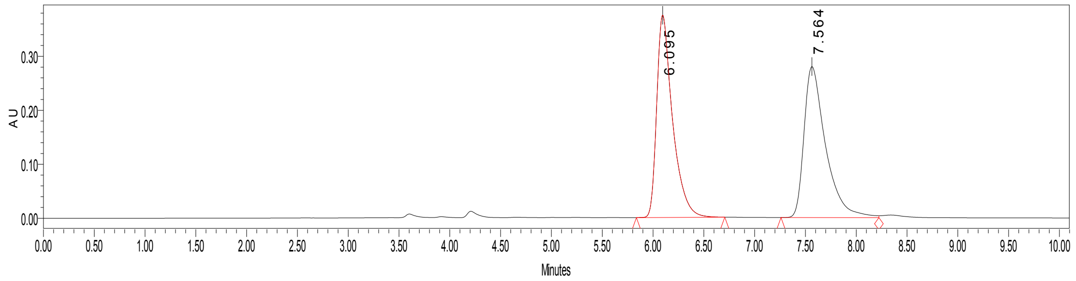

	Retention Time	% Area
1	6.095	49.56
2	7.564	50.44

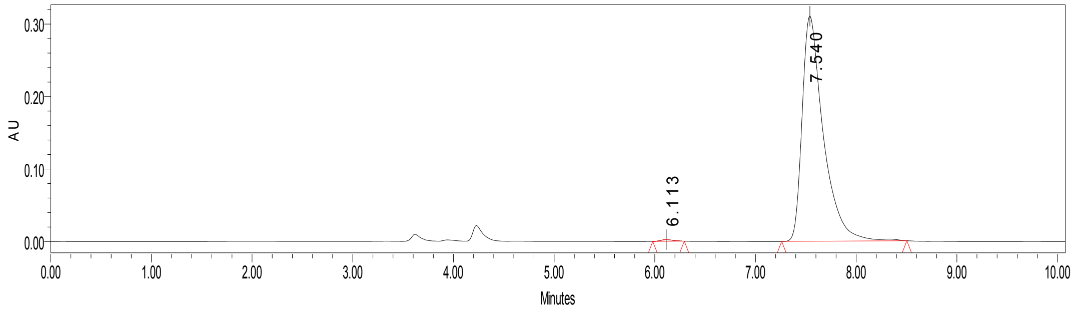

	Retention Time	% Area
1	6.113	0.43
2	7.540	99.57

tert-butyl 2-(((2-methoxyphenyl)amino)methyl)-1-oxo-1,2,3,4-tetrahydronaphthalene

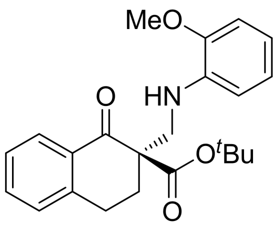

-2-carboxylate (**4k**): Purified by flash chromatography (petroleum ether: EtOAc = 10:1) to afford a yellow oil in 50% yield, 91% ee; $[\alpha]_D^{20}$ = 86.9 (c = 0.38, CH_2Cl_2). HPLC (Chiralpak IC, hexane/*i*-PrOH = 90:10, flow rate 1.0 mL/min, λ = 254 nm) retention time: 9.76 min (minor), 15.14 min (major). ^{1}H NMR (400 MHz, $CDCl_3$): δ 7.99–7.97 (m, 1H), 7.42–7.38 (m, 1H), 7.27–7.23 (m, 1H), 7.15–7.13 (m, 1H), 6.70–6.68 (m, 2H), 6.58–6.56 (m, 2H), 4.30 (s, 1H), 3.66 (s, 3H), 3.54–3.51 (d, *J* = 12.8 Hz, 1H), 3.42–3.39 (d, *J* = 12.8 Hz, 1H), 3.05–2.96 (m, 1H), 2.89–2.83 (m, 1H), 2.45–2.40 (m, 1H), 2.22–2.14 (m, 1H), 1.22 (s, 9H) ppm. ^{13}C NMR (100 MHz, $CDCl_3$): δ 196.9, 170.5, 152.1, 142.8, 133.5, 132.6, 128.7, 127.6, 126.8, 114.8, 114.7, 82.6, 59.3, 55.8, 49.6, 30.9, 27.8, 26.2 ppm. HRMS (ESI-TOF) calcd for $C_{23}H_{27}NNaO_4$ ([M+Na$^+$]) = 404.1838, Found 404.1837.

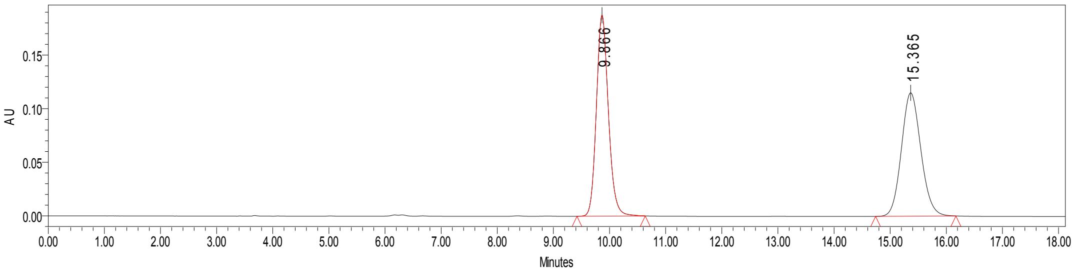

	Retention Time	% Area
1	9.866	50.12
2	15.365	49.88

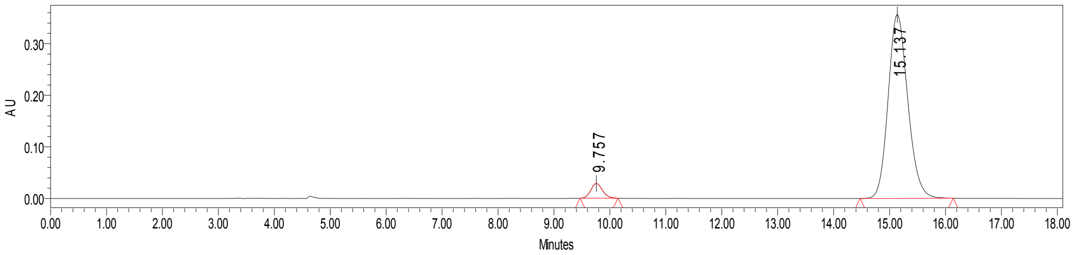

	Retention Time	% Area
1	9.757	4.54
2	15.137	95.46

Adamantan-1-yl 1-oxo-2-((phenylamino)methyl)-1,2,3,4-tetrahydronaphthalene-2-carboxylate (**4l**): Purified by flash chromatography (petroleum ether: EtOAc = 10:1) to afford a white solid in 99% yield, 93% ee; mp 101–102 °C; $[\alpha]_D^{20}$ = 83.8 (c = 0.86, CH_2Cl_2). HPLC (Chiralpak IC, hexane/*i*-PrOH = 90:10, flow rate 1.0 mL/min, λ = 254 nm) retention time: 10.71 min (major), 11.64 min (minor). ^1H NMR (400 MHz, $CDCl_3$): δ 7.99–7.97 (d, *J* = 8.0 Hz, 1H), 7.42–7.38 (m, 1H), 7.26–7.23 (m, 1H), 7.15–7.13 (m, 1H), 7.10–7.06 (m, 2H), 6.61–6.58 (m, 3H), 4.49 (s, 1H), 3.64–3.61 (d, *J* = 12.0 Hz, 1H), 3.47–3.44 (d, *J* = 12.0 Hz, 1H), 3.07–2.98 (m, 1H), 2.88–2.82 (m, 1H), 2.45–2.40 (m, 1H), 2.20–2.14 (m, 1H), 1.98 (s, 3H), 1.84 (s, 6H), 1.48 (s, 6H) ppm. ^{13}C NMR (100 MHz, $CDCl_3$): δ 196.8, 170.1, 148.5, 142.8, 133.5, 132.7, 129.2, 128.7, 127.6, 126.8, 117.4, 113.2, 82.8, 59.5, 48.2, 41.0, 36.0, 30.9, 30.8, 26.2 ppm. HRMS (ESI-TOF) calcd for $C_{28}H_{31}NNaO_3$ ($[M+Na^+]$) = 452.2202, Found 452.2201.

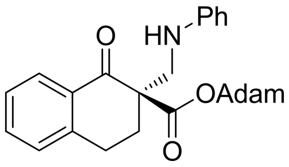

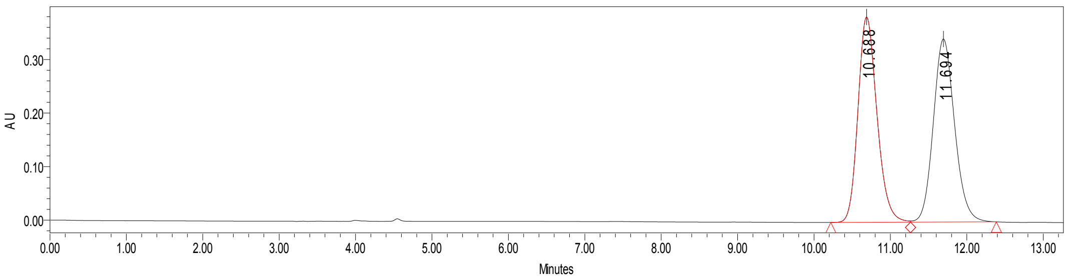

	Retention Time	% Area
1	10.688	50.03
2	11.694	49.97

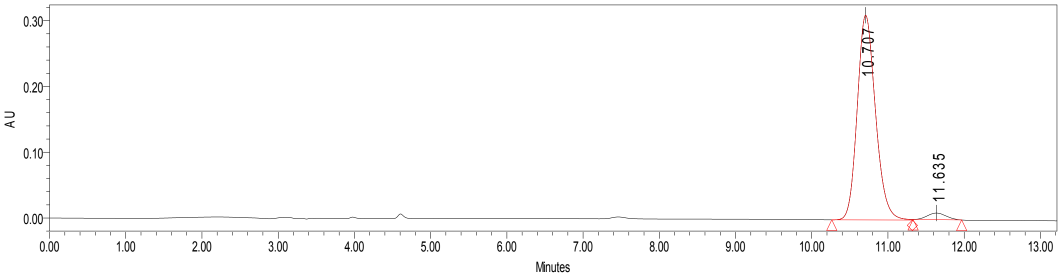

	Retention Time	% Area
1	10.707	96.76
2	11.635	3.24

N-(*tert*-butyl)-1-oxo-2-((phenylamino)methyl)-1,2,3,4-tetrahydronaphthalene-2-carboxamide (**5a**): Purified by flash chromatography (petroleum ether: EtOAc = 10:1) to afford a white solid in 98% yield, 97% ee; mp 78–80 °C; $[\alpha]_D^{23}$ = 77.7 (c = 0.69, CH_2Cl_2). HPLC (Chiralpak IC, hexane/*i*-PrOH = 80:20, flow rate 1.0 mL/min, λ = 254 nm) retention time: 6.39 min (minor), 8.35 min (major). ^{1}H NMR (400 MHz, $CDCl_3$): δ 8.00–7.98 (d, *J* = 8.0 Hz, 1H), 7.45–7.42 (m, 1H), 7.27–7.24 (m, 1H), 7.18–7.15 (m, 1H), 7.09–7.05 (m, 2H), 6.63–6.59 (m, 1H), 6.56–6.54 (m, 2H), 6.41(s, 1H), 4.07 (s, 1H), 3.58–3.47 (dd, *J* = 30.0, 12.8 Hz, 2H), 3.08–3.00 (m, 1H), 2.83–2.76 (m, 1H), 2.68–2.63 (m, 1H), 2.18–2.11 (m, 1H), 1.16 (s, 9H) ppm. ^{13}C NMR (100 MHz, $CDCl_3$): δ 200.3, 167.3, 148.1, 144.8, 134.5, 131.7, 129.3, 128.9, 128.0, 126.8, 117.7, 113.0, 59.2, 51.6, 50.2, 29.4, 28.5, 26.0 ppm. HRMS (ESI-TOF) calcd for $C_{22}H_{26}N_2NaO_2$ ($[M+Na^+]$) = 373.1892, Found 373.1885.

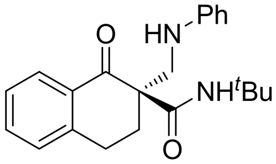

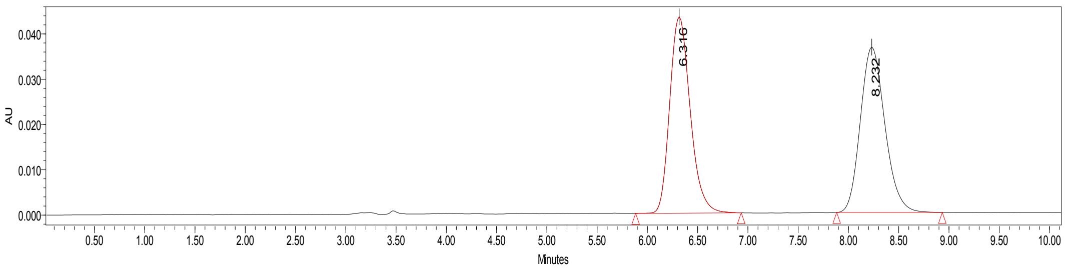

	Retention Time	% Area
1	6.316	49.78
2	8.232	50.22

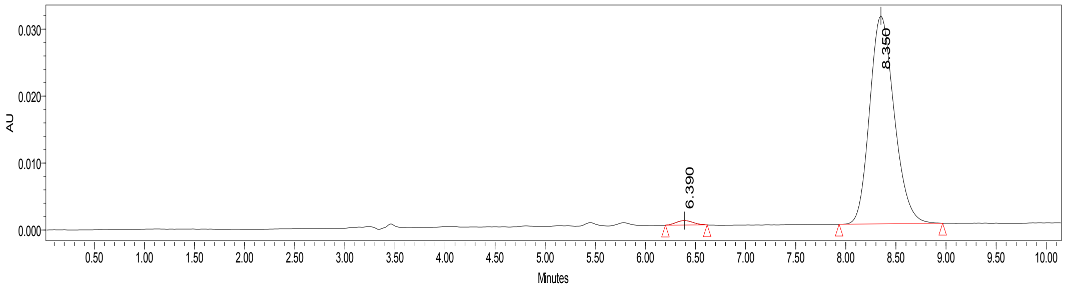

	Retention Time	% Area
1	6.390	1.47
2	8.350	98.53

N-(*tert*-butyl)-5-methoxy-1-oxo-2-((phenylamino)methyl)-1,2,3,4-tetrahydronaphthalene-2-carboxamide (**5b**):

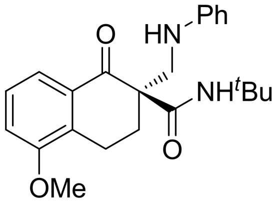

Purified by flash chromatography (petroleum ether: EtOAc = 10:1) to afford a white solid in 98% yield, 96% ee; mp 92–94 °C; $[\alpha]_D^{27}$ = 76.3 (c = 0.74, CH_2Cl_2). HPLC (Chiralpak IC, hexane/*i*-PrOH = 80:20, flow rate 1.0 mL/min, λ = 254 nm) retention time: 9.13 min (minor), 15.97 min (major). ^{1}H NMR (400 MHz, $CDCl_3$): δ 7.68–7.66 (d, *J* = 7.6 Hz, 1H), 7.31–7.27 (m, 1H), 7.16–7.12 (m, 2H), 7.05–7.03 (m, 1H), 6.70–6.66 (m, 1H), 6.63–6.61 (m, 2H), 6.44 (s, 1H), 4.18(s, 1H), 3.85(s, 3H), 3.62–3.54 (dd, *J* = 22.8, 12.8 Hz, 2H), 3.02–2.83 (m, 2H), 2.71–2.65 (m, 1H), 2.22–2.15 (m, 1H), 1.22 (s, 9H) ppm. ^{13}C NMR (100 MHz, $CDCl_3$): δ 200.3, 167.6, 156.7, 148.1, 133.8, 132.6, 129.2, 127.0, 119.4, 117.6, 115.1, 113.0, 58.5, 55.7, 51.6, 49.8, 28.8, 28.5, 19.4 ppm. HRMS (ESI-TOF) calcd for $C_{23}H_{28}N_2NaO_3$ ($[M+Na^+]$) = 403.1998, Found 403.1991.

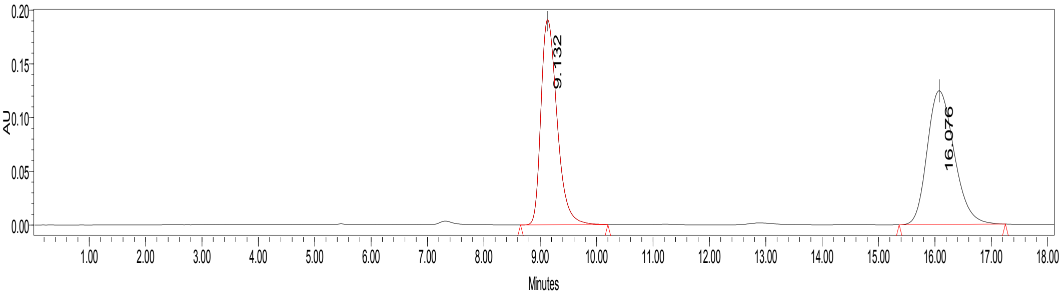

	Retention Time	% Area
1	9.132	47.79
2	16.076	52.21

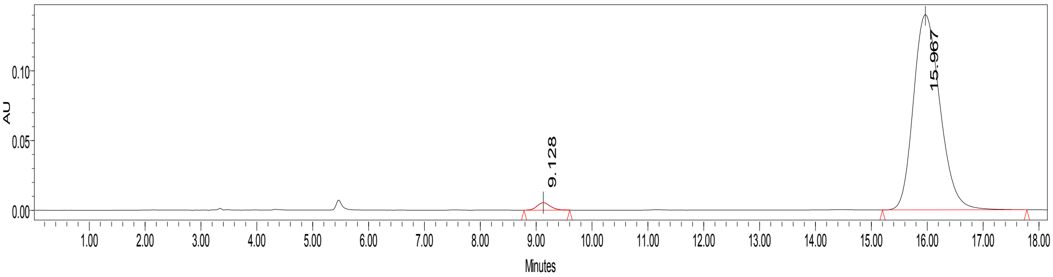

	Retention Time	% Area
1	9.128	1.87
2	15.967	98.13

N-(*tert*-butyl)-6-methoxy-1-oxo-2-((phenylamino)methyl)-1,2,3,4-tetrahydronaphthalene-2-carboxamide (**5c**): Purified by flash chromatography (petroleum ether: EtOAc = 10:1) to afford a white solid in 77% yield, 96% ee; mp 128–130 °C; $[\alpha]_D^{27}$ = 83.0 (c = 0.58, CH_2Cl_2). HPLC (Chiralpak IC, hexane/*i*-PrOH = 80:20, flow rate 1.0 mL/min, λ = 254 nm) retention time: 8.27 min (minor), 12.73 min (major). ^{1}H NMR (400 MHz, $CDCl_3$): δ 8.05–8.02 (d, *J* = 8.8 Hz, 1H), 7.16–7.12 (m, 2H), 6.86–6.83 (m, 1H), 6.68–6.61 (m, 4H), 6.55 (s, 1H), 4.15 (s, 1H), 3.86 (s, 3H), 3.65–3.52 (m, 2H), 3.12–3.04 (m, 1H), 2.85–2.79 (m, 1H), 2.74–2.69 (m, 1H), 2.22–2.15 (m, 1H), 1.24 (s, 9H) ppm. ^{13}C NMR (100 MHz, $CDCl_3$): δ 198.7, 167.5, 164.5, 148.2, 147.6, 130.7, 129.2, 125.3, 117.5, 113.8, 112.9, 112.3, 58.9, 55.6, 51.5, 50.4, 29.4, 28.5, 26.5 ppm. HRMS (ESI-TOF) calcd for $C_{23}H_{28}N_2NaO_3$ ($[M+Na^+]$) = 403.1998, Found 403.1995.

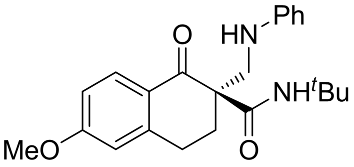

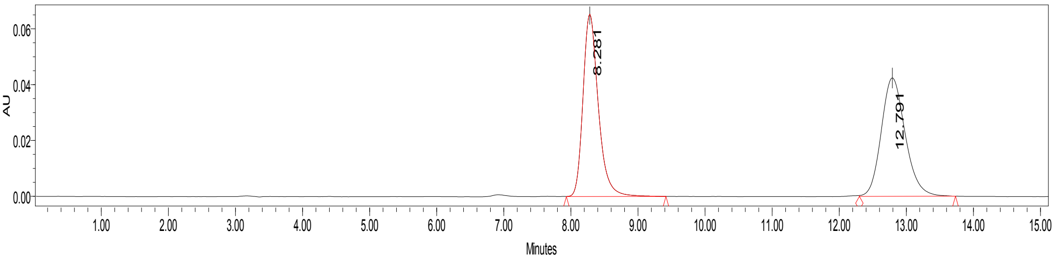

	Retention Time	% Area
1	8.281	49.57
2	12.791	50.43

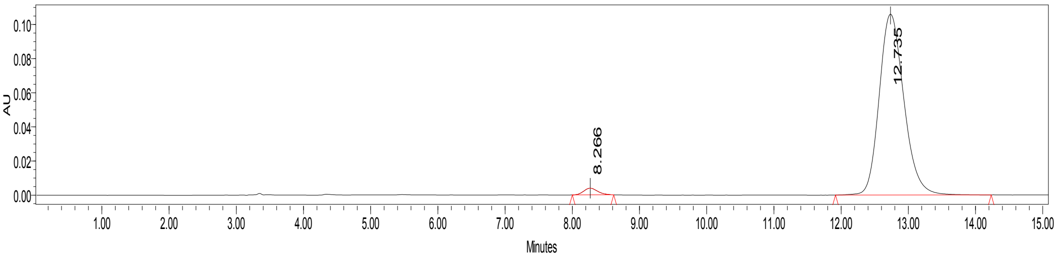

	Retention Time	% Area
1	8.266	2.08
2	12.735	97.92

N-(*tert*-butyl)-7-methoxy-1-oxo-2-((phenylamino)methyl)-1,2,3,4-tetrahydronaphthalene-2-carboxamide (**5d**): Purified by flash chromatography (petroleum ether: EtOAc = 10:1) to afford a white solid in 95% yield, 96% ee; mp 150–152 °C; $[\alpha]_D^{27}$ = 79.7 (c = 0.72, CH_2Cl_2). HPLC (Chiralpak IC, hexane/*i*-PrOH = 80:20, flow rate 1.0 mL/min, λ = 254 nm) retention time: 8.78 min (minor), 14.82 min (major). ^1H NMR (400 MHz, $CDCl_3$): δ 7.53–7.52 (d, *J* = 2.8 Hz, 1H), 7.16–7.08 (m, 4H), 6.70–6.67 (m, 1H), 6.64–6.62 (m, 2H), 6.47 (s, 1H), 4.16 (s, 1H), 3.85 (s, 3H), 3.65–3.54 (dd, *J* = 29.2, 12.4 Hz, 2H), 3.08–3.00 (m, 1H), 2.85–2.78 (m, 1H), 2.72–2.67 (m, 1H), 2.25–2.17 (m, 1H), 1.25 (s, 9H) ppm. ^{13}C NMR (100 MHz, $CDCl_3$): δ 200.1, 167.4, 158.4, 148.1, 137.4, 132.4, 130.1, 129.3, 122.8, 117.7, 113.0, 109.9, 59.0, 55.5, 51.6, 50.2, 29.7, 28.5, 25.2 ppm. HRMS (ESI-TOF) calcd for $C_{23}H_{28}N_2NaO_3$ ($[M+Na^+]$) = 403.1998, Found 403.1991.

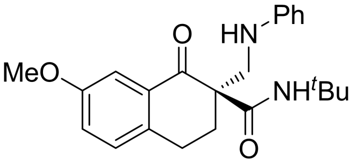

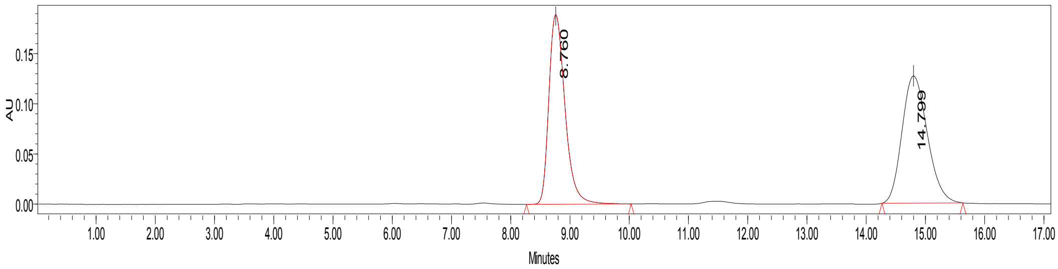

	Retention Time	% Area
1	8.760	48.64
2	14.799	51.36

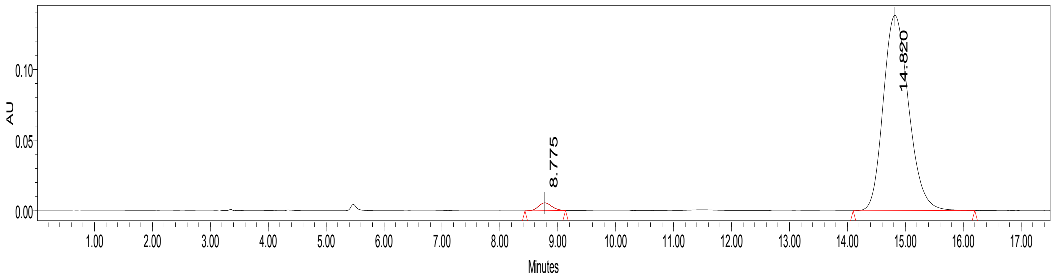

	Retention Time	% Area
1	8.775	2.05
2	14.820	97.95

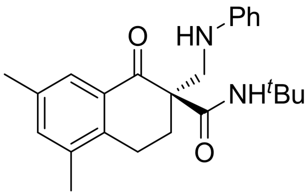

N-(*tert*-butyl)-5,7-dimethyl-1-oxo-2-((phenylamino)methyl)-1,2,3,4-tetrahydronaphthalene-2-carboxamide (**5e**): Purified by flash chromatography (petroleum ether: EtOAc = 10:1) to afford a white solid in 75% yield, 98% ee; mp 152–154 °C; $[\alpha]_D^{27}$ = 67.7 (c = 0.57, CH_2Cl_2). HPLC (Chiralpak IC, hexane/*i*-PrOH = 80:20, flow rate 1.0 mL/min, λ = 254 nm) retention time: 7.24 min (minor), 14.10 min (major). ^{1}H NMR (400 MHz, $CDCl_3$): δ 7.75 (s, 1H), 7.22 (s, 1H), 7.16–7.12 (m, 2H), 6.70–6.66 (m, 1H), 6.63–6.61 (m, 2H), 6.46 (s, 1H), 4.17 (s, 1H), 3.63–3.52 (m, 2H), 2.92–2.77 (m, 2H), 2.73–2.67 (m, 1H), 2.34 (s, 3H), 2.25 (s, 3H), 2.23–2.17 (m, 1H), 1.23 (s, 9H) ppm. ^{13}C NMR (100 MHz, $CDCl_3$): δ 200.7, 167.6, 148.1, 140.3, 136.9, 136.4, 135.9, 131.6, 129.2, 125.9, 117.6, 113.0, 58.5, 51.6, 50.0, 28.8, 28.5, 22.7, 20.9, 19.2 ppm. HRMS (ESI-TOF) calcd for $C_{24}H_{30}N_2NaO_2$ ([M+Na$^+$]) = 401.2205, Found 401.2199.

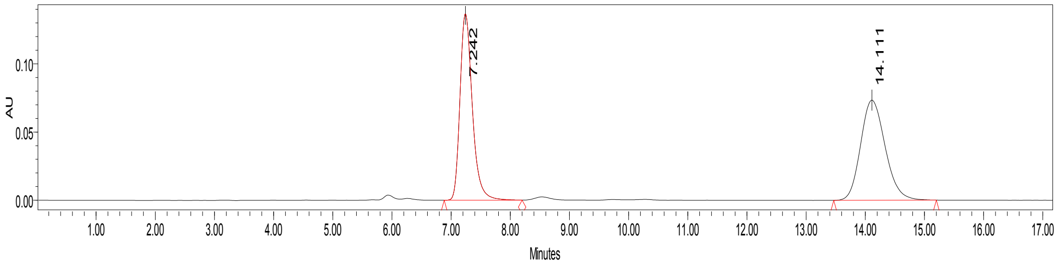

	Retention Time	% Area
1	7.242	48.38
2	14.111	51.62

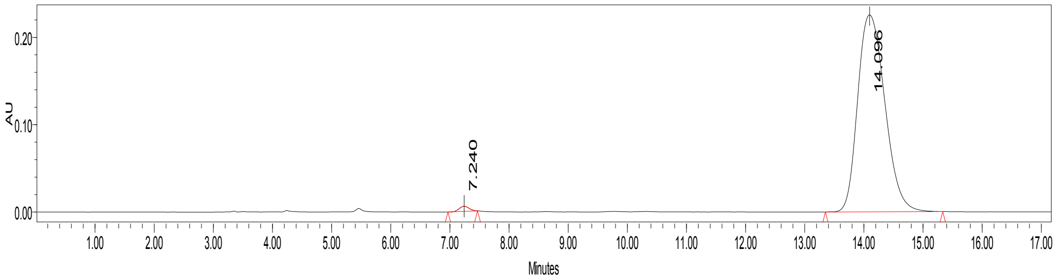

	Retention Time	% Area
1	7.240	0.93
2	14.096	99.07

N-(*tert*-butyl)-6,7-dimethoxy-1-oxo-2-((phenylamino)methyl)-1,2,3,4-tetrahydronaphthalene-2-carboxamide (**5f**): Purified by flash chromatography (petroleum ether: EtOAc = 10:1) to afford a yellow oil in 71% yield, 93% ee; $[\alpha]_D^{27}$ = 85.2 (c = 0.58, CH_2Cl_2). HPLC (Chiralpak IC, hexane/*i*-PrOH = 80:20, flow rate 1.0 mL/min, λ = 254 nm) retention time: 12.98 min (minor), 23.12 min (major). ^{1}H NMR (400 MHz, $CDCl_3$): δ 7.52 (s, 1H), 7.16–7.13 (m, 2H), 6.70–6.66 (m, 1H), 6.64–6.62 (m, 3H), 6.56 (s, 1H), 4.16 (s, 1H), 3.94–3.93 (d, *J* = 3.6 Hz, 6H), 3.65–3.53 (dd, *J* = 34.4, 12.4 Hz, 2H), 3.10–3.02 (m, 1H), 2.82–2.68 (m, 2H), 2.25–2.18 (m, 1H), 1.25 (s, 9H) ppm. ^{13}C NMR (100 MHz, $CDCl_3$): δ 198.6, 167.6, 154.6, 148.1, 140.2, 129.2, 124.8, 117.6, 113.0, 110.1, 109.1, 58.5, 56.2, 56.0, 51.5, 50.3, 29.6, 28.5, 25.9 ppm. HRMS (ESI-TOF) calcd for $C_{24}H_{30}N_2NaO_4$ ($[M+Na^+]$) = 433.2103, Found 433.2107.

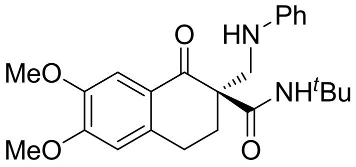

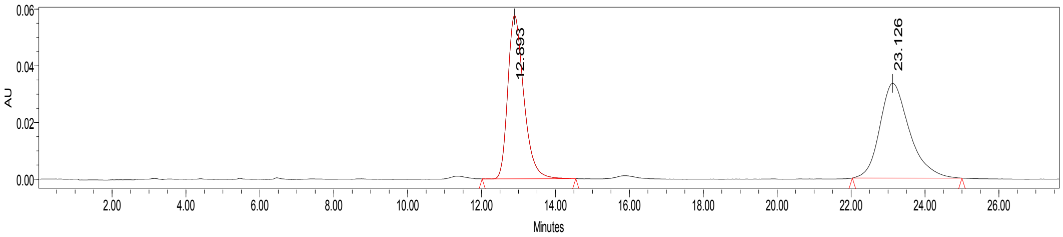

	Retention Time	% Area
1	12.893	48.21
2	23.126	51.79

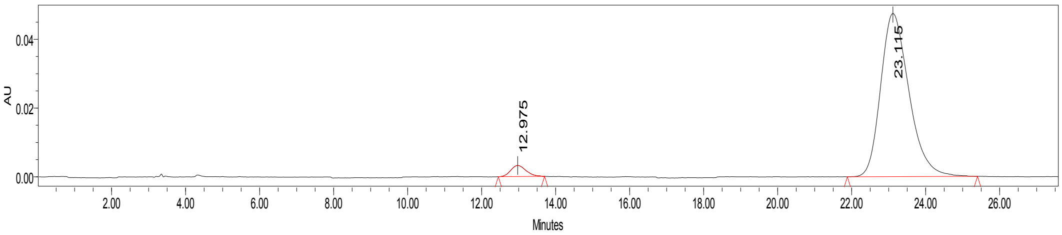

	Retention Time	% Area
1	12.975	3.53
2	23.115	96.47

N-(*tert*-butyl)-2-(((4-(tert-butyl)phenyl)amino)methyl)-1-oxo-1,2,3,4-tetrahydronaphthalene-2-carboxamide (**5g**): Purified by flash chromatography (petroleum ether: EtOAc = 10:1) to afford a white solid in 95% yield, 95% ee; mp 106–108 °C; $[\alpha]_D^{27}$ = 76.7 (c = 0.78, CH_2Cl_2). HPLC (Chiralpak IC, hexane/*i*-PrOH = 80:20, flow rate 1.0 mL/min, λ = 254 nm) retention time: 5.86 min (minor), 10.15 min (major). ^{1}H NMR (400 MHz, $CDCl_3$): δ 8.07–8.05 (m, 1H), 7.52–7.48 (m, 1H), 7.34–7.30 (m, 1H), 7.24–7.22 (m, 1H), 7.19–7.17 (m, 2H), 6.60–6.58 (m, 2H), 6.53 (s, 1H), 4.04 (s, 1H), 3.64–3.52 (dd, *J* = 34.8, 12.4 Hz, 2H), 3.16–3.08 (m, 1H), 2.91–2.84 (m, 1H), 2.76–2.70 (m, 1H), 2.27–2.20 (m, 1H), 1.26–1.24 (m, 18H) ppm. ^{13}C NMR (100 MHz, $CDCl_3$): δ 200.3, 167.4, 145.7, 144.8, 140.5, 134.4, 131.7, 128.8, 128.0, 126.8, 126.0, 112.8, 59.2, 51.6, 50.6, 33.9, 31.6, 29.3, 28.5, 26.0 ppm. HRMS (ESI-TOF) calcd for $C_{26}H_{34}N_2NaO_2$ ([M+Na$^+$]) = 429.2518, Found 429.2512.

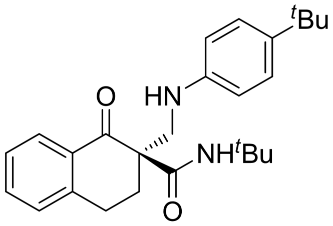

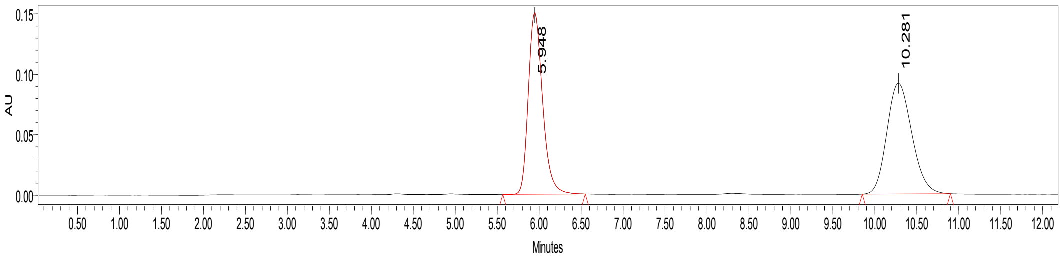

	Retention Time	% Area
1	5.948	48.40
2	10.281	51.60

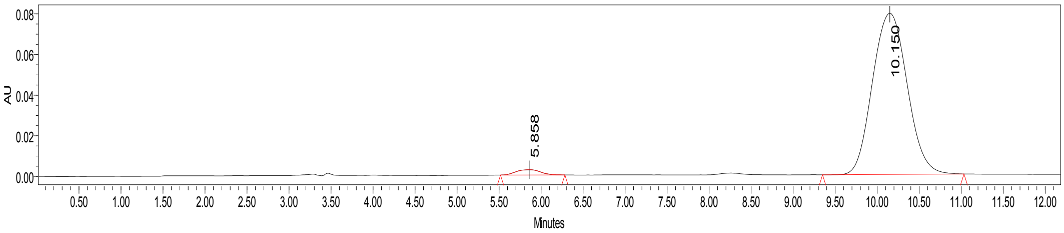

	Retention Time	% Area

1	5.858	2.24
2	10.150	97.76

N-(*tert*-butyl)-2-(((4-methoxyphenyl)amino)methyl)-1-oxo-1,2,3,4-tetrahydronaphthalene-2-carboxamide (**5h**): Purified by flash chromatography (petroleum ether: EtOAc = 10:1) to afford a white solid in 87% yield, 84% ee; mp 137–138 °C; $[\alpha]_D^{28}$ = 68.1 (c = 0.66, CH_2Cl_2). HPLC (Chiralpak IC, hexane/*i*-PrOH = 80:20, flow rate 1.0 mL/min, λ = 254 nm) retention time: 10.12 min (minor), 14.62 min (major). ^{1}H NMR (400 MHz, $CDCl_3$): δ 8.07–8.05 (m, 1H), 7.53–7.48 (m, 1H), 7.35–7.31 (m, 1H), 7.25–7.23 (m, 1H), 6.76–6.74 (m, 2H), 6.61–6.58 (m, 3H), 3.73 (s, 3H), 3.60–3.49 (dd, *J* = 30.8, 12.8 Hz, 2H), 3.16–3.08 (m, 1H), 2.91–2.84 (m, 1H), 2.74–2.69 (m, 1H), 2.27–2.20 (m, 1H), 1.25 (s, 9H) ppm. ^{13}C NMR (100 MHz, $CDCl_3$): δ 200.2, 167.6, 152.3, 144.7, 142.3, 134.4, 131.8, 128.8, 128.0, 126.8, 114.8, 114.5, 59.2, 55.8, 51.6, 51.5, 29.4, 28.5, 26.0 ppm. HRMS (ESI-TOF) calcd for $C_{23}H_{28}N_2NaO_3$ ([M+Na$^+$]) = 403.1998, Found 403.2000.

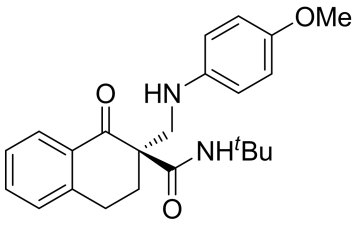

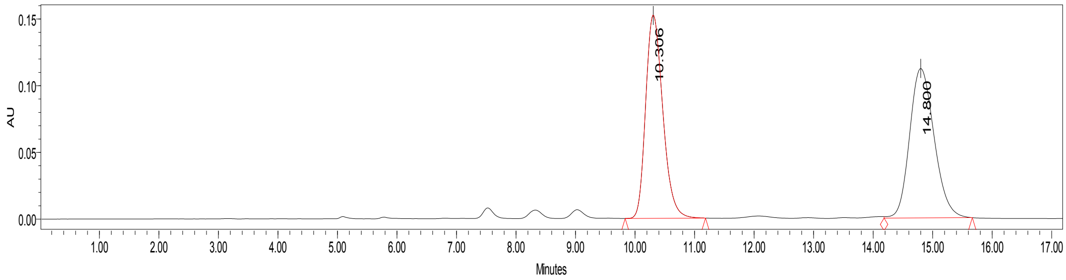

	Retention Time	% Area
1	10.306	48.74
2	14.800	51.26

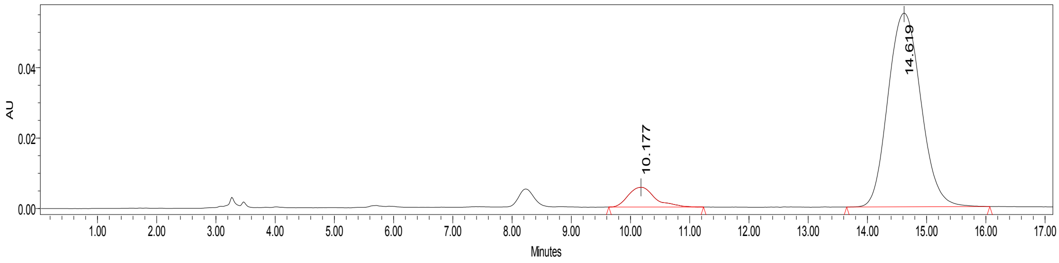

	Retention Time	% Area
1	10.177	7.71
2	14.619	92.29

N-(*tert*-butyl)-1-oxo-2-((o-tolylamino)methyl)-1,2,3,4-tetrahydronaphthalene-2-carboxamide (**5i**): Purified by flash chromatography (petroleum ether: EtOAc = 10:1) to afford a yellow oil in 96% yield, 98% ee; $[\alpha]_D^{28}$ = 75.9 (c = 0.70, CH_2Cl_2). HPLC (Chiralpak IC,

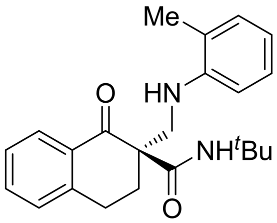

hexane/*i*-PrOH = 80:20, flow rate 1.0 mL/min, λ = 254 nm) retention time: 14.18 min (minor), 15.60 min (major). ^{1}H NMR (400 MHz, $CDCl_3$): δ 8.08–8.06 (m, 1H), 7.54–7.50 (m, 1H), 7.36–7.32 (m, 1H), 7.26–7.24 (m, 1H), 7.11–7.07 (m, 1H), 7.05–7.03 (m, 1H), 6.66–6.59 (m, 3H), 4.19 (s, 1H), 3.63–3.56 (m, 2H), 3.15–3.07 (m, 1H), 2.93–2.86 (m, 1H), 2.81–2.75 (m, 1H), 2.26–2.19 (m, 1H), 2.15 (s, 3H), 1.25 (s, 9H) ppm. ^{13}C NMR (100 MHz, $CDCl_3$): δ 200.2, 167.5, 146.0, 144.8, 134.5, 131.7, 130.1, 128.9, 128.1, 127.1, 126.9, 122.4, 117.1, 109.7, 58.5, 51.6, 49.9, 29.6, 28.5, 26.0, 17.5 ppm. HRMS (ESI-TOF) calcd for $C_{23}H_{28}N_2NaO_2$ ([M+Na$^+$]) = 387.2048, Found 387.2041.

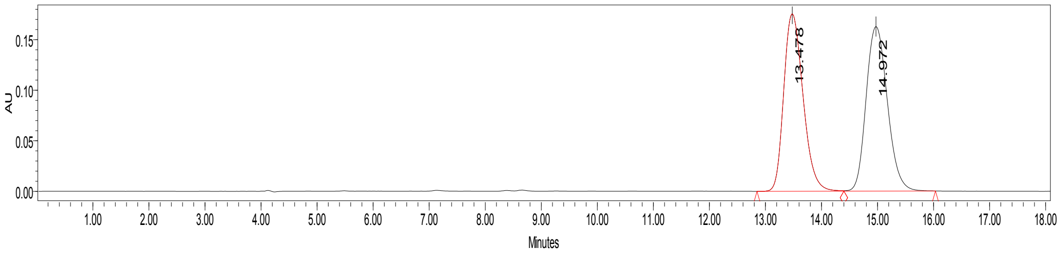

	Retention Time	% Area
1	13.478	49.49
2	14.972	50.51

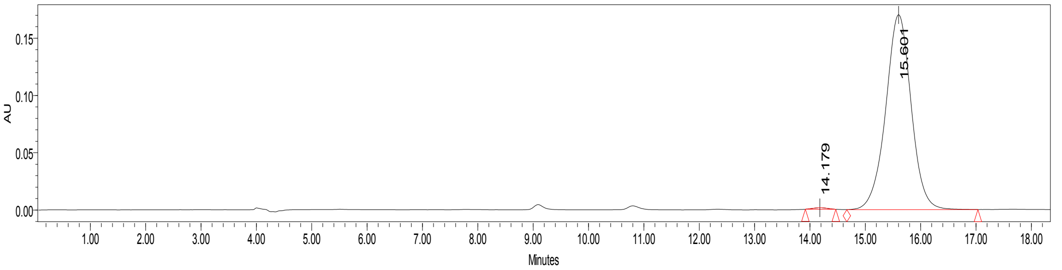

	Retention Time	% Area
1	14.179	0.44
2	15.601	99.56

N-(*tert*-butyl)-2-(((2-methoxyphenyl)amino)methyl)-1-oxo-1,2,3,4-tetrahydronaphthalene-2-carboxamide (**5j**): Purified by flash chromatography (petroleum ether: EtOAc = 10:1) to afford a yellow oil in 97% yield, 99% ee; $[\alpha]_D^{28}$ = 86.8 (c = 0.74, CH_2Cl_2). HPLC (Chiralpak IC, hexane/*i*-PrOH = 80:20, flow rate 1.0 mL/min, λ = 254 nm) retention time: 7.42 min (minor), 12.09 min (major). ^{1}H NMR (400 MHz, $CDCl_3$): δ 8.07–8.05 (m, 1H), 7.52–7.47 (m, 1H), 7.34–7.30 (m, 1H), 7.25–7.22 (m, 1H), 6.85–6.81 (m, 1H), 6.75–6.73 (m, 1H), 6.66–6.62 (m, 2H), 6.55 (s, 1H), 4.68 (s, 1H), 3.81 (s, 3H), 3.70–3.66 (m, 1H), 3.61–3.57 (m, 1H), 3.19–3.11 (m, 1H), 2.91–2.85 (m, 1H), 2.80–2.74 (m, 1H), 2.27–2.20 (m, 1H), 1.24 (s, 9H) ppm. ^{13}C NMR (100 MHz, $CDCl_3$): δ 200.1, 167.2, 146.9, 144.8, 138.0, 134.3, 131.8, 128.8, 128.0, 126.7, 121.2,

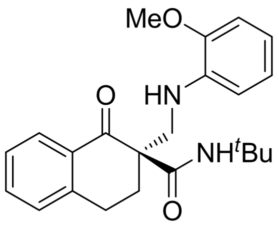

116.7, 110.1, 109.6, 59.4, 55.5, 51.5, 50.1, 29.3, 28.5, 26.1 ppm. HRMS (ESI-TOF) calcd for $C_{23}H_{28}N_2NaO_3$ ([M+Na$^+$]) = 403.1998, Found 403.1994.

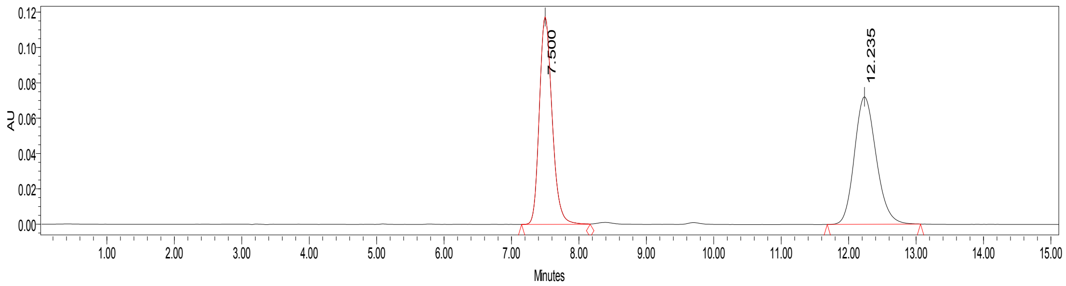

	Retention Time	% Area
1	7.500	48.83
2	12.235	51.17

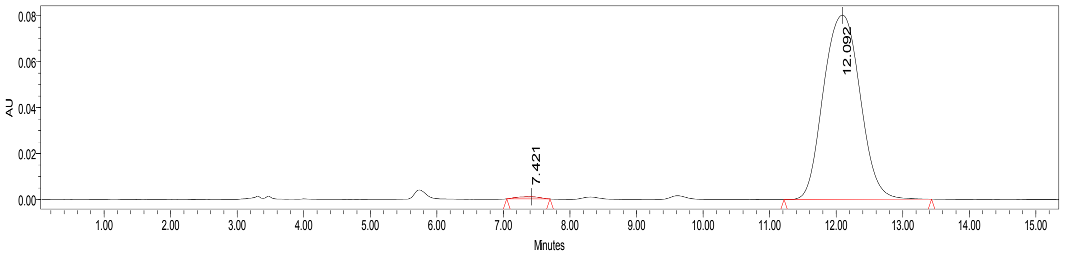

	Retention Time	% Area
1	7.421	0.74
2	12.092	99.26

N-(*tert*-butyl)-1-oxo-2-((phenylamino)methyl)-2,3-dihydro-1H-indene-2-carboxamide

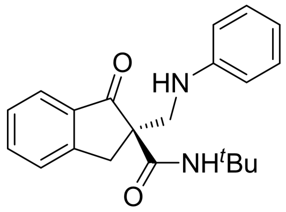

(**5k**): Purified by flash chromatography (petroleum ether: EtOAc = 10:1) to afford a yellow solid in 99% yield, 55% ee; mp 122–124 °C; $[\alpha]_D^{23}$ = -4.5 (c = 0.67, CH_2Cl_2). HPLC (Chiralpak IC, hexane/*i*-PrOH = 80:20, flow rate 1.0 mL/min, λ = 254 nm) retention time: 7.15 min (major), 7.67 min (minor). ^{1}H NMR (400 MHz, $CDCl_3$): δ 7.69–7.67 (d, *J* = 7.6 Hz, 1H), 7.58–7.54 (t, *J* = 7.4 Hz, 1H), 7.40–7.38 (d, *J* = 7.6 Hz, 1H), 7.34–7.30 (t, *J* = 7.4 Hz, 1H), 7.08–7.04 (t, *J* = 7.4 Hz, 2H), 6.64–6.60 (t, *J* = 7.2 Hz, 1H), 6.52–6.50 (d, *J* = 8.0 Hz, 1H), 4.05 (s, 1H), 3.86–3.82 (d, *J* = 18.0 Hz, 1H), 3.51–3.46 (m, 1H), 3.37–3.34 (m, 1H), 3.16–3.12 (d, *J* = 18.0 Hz, 1H), 1.22 (s, 9H) ppm. ^{13}C NMR (100 MHz, $CDCl_3$): δ 206.4, 167.5, 153.7, 147.6, 136.2, 134.9, 129.3, 127.7, 126.7, 124.6, 118.0, 113.0, 61.4, 52.0, 51.4, 34.9, 28.5 ppm. HRMS (ESI-TOF) calcd for $C_{21}H_{24}N_2NaO_2$ ([M+Na$^+$]) = 359.1735, Found 359.1736.

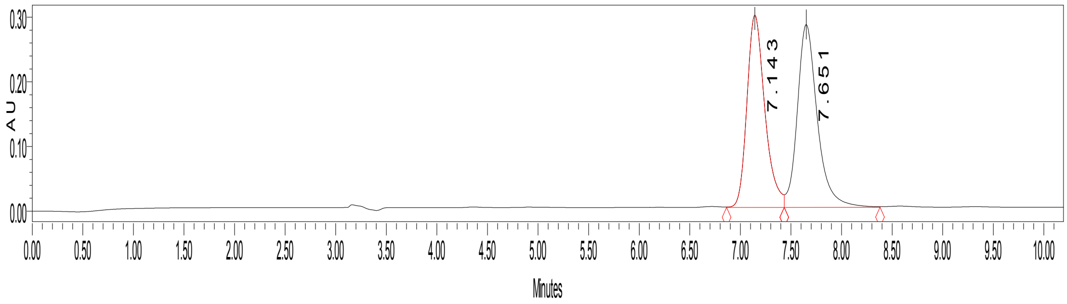

	Retention Time	% Area
1	7.143	48.46
2	7.651	51.54

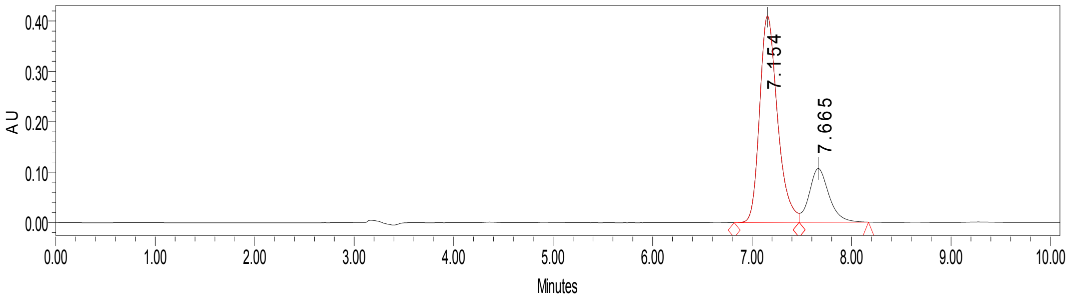

	Retention Time	% Area
1	7.154	77.65
2	7.665	22.35

N-(*tert*-butyl)-5-oxo-6-((phenylamino)methyl)-6,7,8,9-tetrahydro-5H-benzo[7]annulene-6-carboxamide (**5l**): Purified by flash chromatography (petroleum ether: EtOAc = 10:1) to afford a white solid in 92% yield, 0% ee; mp 94–95 °C. HPLC (Chiralpak IC, hexane/*i*-PrOH = 90:10, flow rate 1.0 mL/min, λ = 254 nm) retention time: 7.09 min, 10.20 min. ^{1}H NMR (400 MHz, $CDCl_3$): δ 7.44–7.36 (m, 2H), 7.29–7.25 (m, 1H), 7.16–7.12 (m, 3H), 6.75 (s, 1H), 6.71–6.68 (m, 1H), 6.62–6.60 (m, 2H), 4.11 (s, 1H), 3.67–3.62 (m, 1H), 3.55–3.51 (m, 1H), 2.81–2.75 (m, 2H), 2.50–2.44 (m, 1H), 2.34–2.26 (m, 1H), 1.79–1.71 (m, 2H), 1.23 (s, 9H) ppm. ^{13}C NMR (100 MHz, $CDCl_3$): δ 212.0, 167.1, 147.8, 139.8, 139.0, 132.4, 129.2, 128.7, 127.9, 126.8, 117.9, 113.3, 62.8, 53.3, 51.4, 31.8, 28.6, 28.4, 23.2 ppm. HRMS (ESI-TOF) calcd for $C_{23}H_{28}N_2NaO_2$ ([M+Na$^+$]) = 387.2048, Found 387.2048.

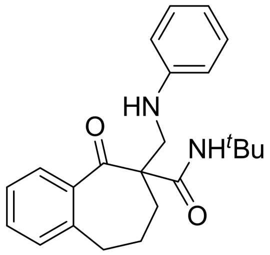

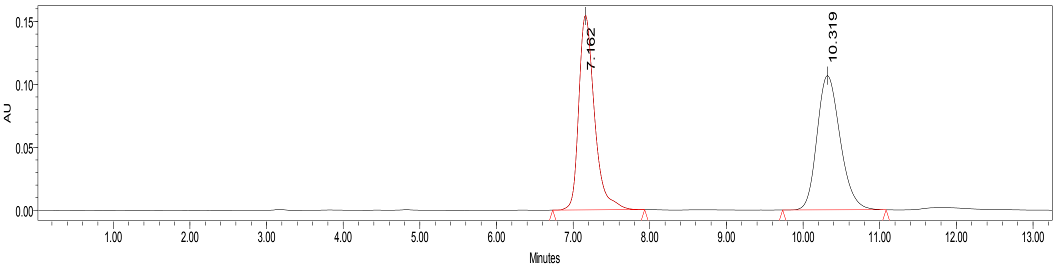

	Retention Time	% Area
1	7.162	49.53
2	10.319	50.47

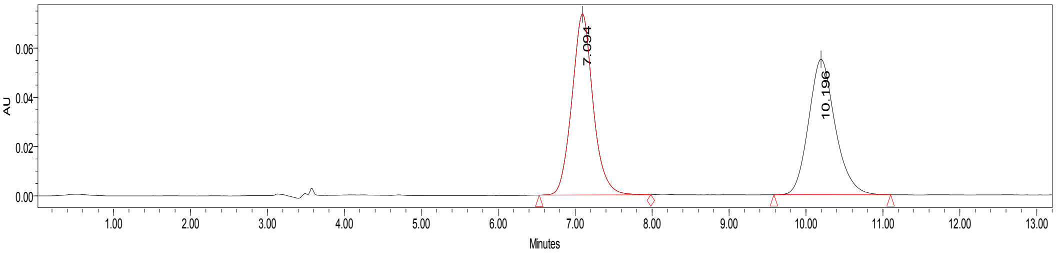

	Retention Time	% Area
1	7.094	51.61
2	10.196	48.39

tert-butyl-1-hydroxy-2-((phenylamino)methyl)-1,2,3,4-tetrahydronaphthalene-2-

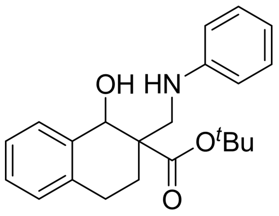

carboxylate (**6**): Purified by flash chromatography (petroleum ether: EtOAc = 10:1) to afford a white solid in 99% yield, 99% ee; mp 116–118 °C; $[\alpha]_D^{23}$ = 94.4 (c = 0.34, CH_2Cl_2). HPLC (Chiralpak IC, hexane/*i*-PrOH = 95:5, flow rate 1.0 mL/min, λ = 254 nm) retention time: 8.20 min (minor), 8.89 min (major). ^{1}H NMR (400 MHz, $CDCl_3$): δ7.62–7.60 (d, *J* = 7.6 Hz, 1H), 7.25–7.14 (m, 4H), 7.07–7.05 (m, 1H), 6.70–6.63 (m, 3H), 4.77–4.75 (d, *J* = 9.2 Hz, 1H), 4.63 (s, 1H), 3.70–3.68 (d, *J* = 10.0 Hz, 1H), 3.59–3.56 (d, *J* = 12.0 Hz, 1H), 3.38–3.35 (d, *J* = 12.0 Hz, 1H), 2.88–2.77 (m, 2H), 2.29–2.25 (m, 1H), 1.91–1.84 (m, 1H), 1.32 (s, 9H) ppm. ^{13}C NMR (100 MHz, $CDCl_3$): δ 174.0, 148.4, 138.7, 134.8, 129.2, 128.3, 127.3, 127.1, 126.5, 117.5, 113.1, 82.2, 74.3, 51.3, 51.2, 27.9, 27.9, 26.0 ppm. HRMS (ESI-TOF) calcd for $C_{22}H_{27}NNaO_3$ ([M+Na$^+$]) = 376.1889, Found 376.1883.

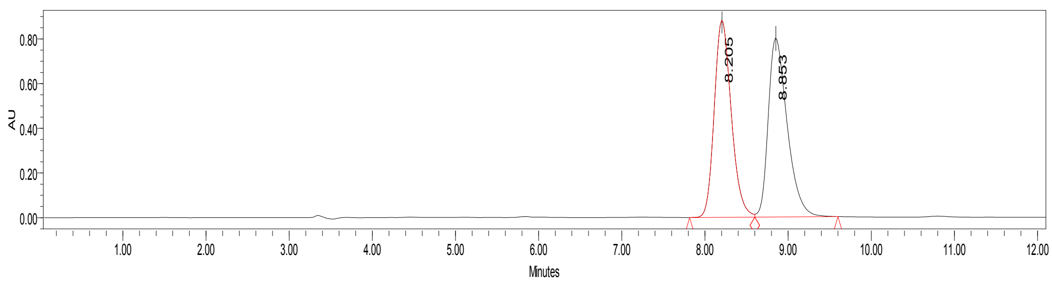

	Retention Time	% Area
1	8.205	49.70
2	8.853	50.30

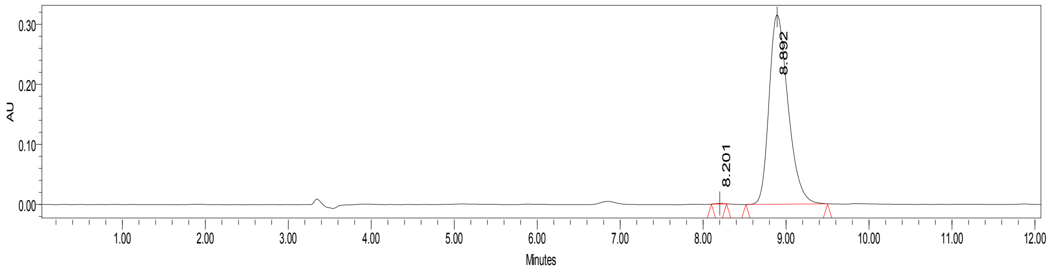

	Retention Time	% Area
1	8.201	0.11
2	8.892	99.89

tert-butyl 2-(((tert-butoxycarbonyl)amino)methyl)-1-oxo-1,2,3,4-tetrahydronaphthal-ene-2-carboxylate (**7**): Purified by flash chromatography (petroleum ether: EtOAc = 10:1) to afford a yellow oil in 60% yield, 94% ee; $[\alpha]_D^{23}$ = 45.5 (c = 0.45, CH_2Cl_2). HPLC (Chiralpak IA, hexane/*i*-PrOH = 90:10, flow rate 1.0 mL/min, λ = 254 nm) retention time: 5.81 min (minor), 7.06 min (major).

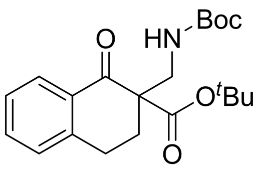

^{1}H NMR (400 MHz, $CDCl_3$): δ 8.04–8.02 (d, *J* = 8.0 Hz, 1H), 7.50–7.46 (m, 1H), 7.34–7.30 (m, 1H), 7.24–7.22 (m, 1H), 6.79 (s, 1H), 3.80–3.75 (m, 1H), 3.49–3.45 (m, 1H), 3.03 (m, 2H), 2.49–2.46 (m, 1H), 2.18–2.13 (m, 1H), 1.42 (s, 9H), 1.37 (s, 9H) ppm. ^{13}C NMR (100 MHz, $CDCl_3$): δ 174.6, 148.3, 137.5, 134.9, 129.2, 128.4, 128.0, 127.5, 126.4, 118.1, 113.9, 81.8, 71.6, 50.6, 47.4, 27.9, 26.6, 25.5 ppm. HRMS (ESI-TOF) calcd for $C_{21}H_{29}NNaO_5$ ($[M+Na^+]$) = 398.1943, Found 398.1940.

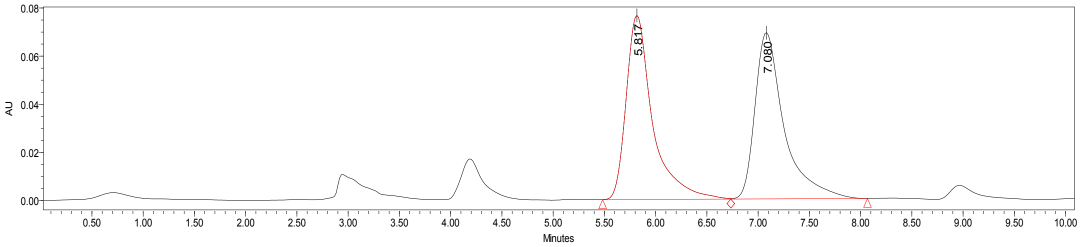

	Retention Time	% Area
1	5.817	50.14
2	7.080	49.86

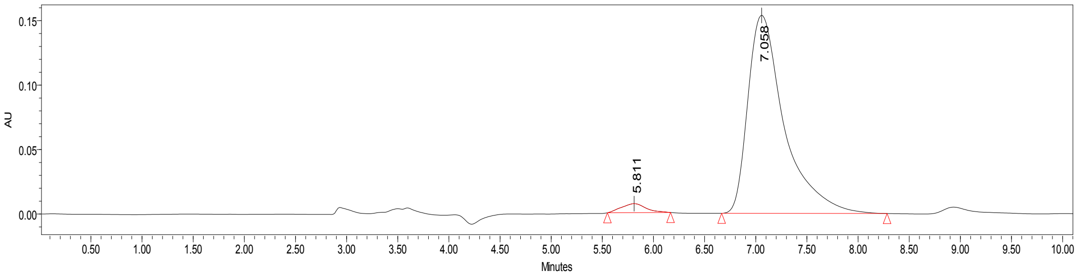

	Retention Time	% Area
1	5.811	2.95
2	7.058	97.05

8. Copies of NMR spectra for the reaction products

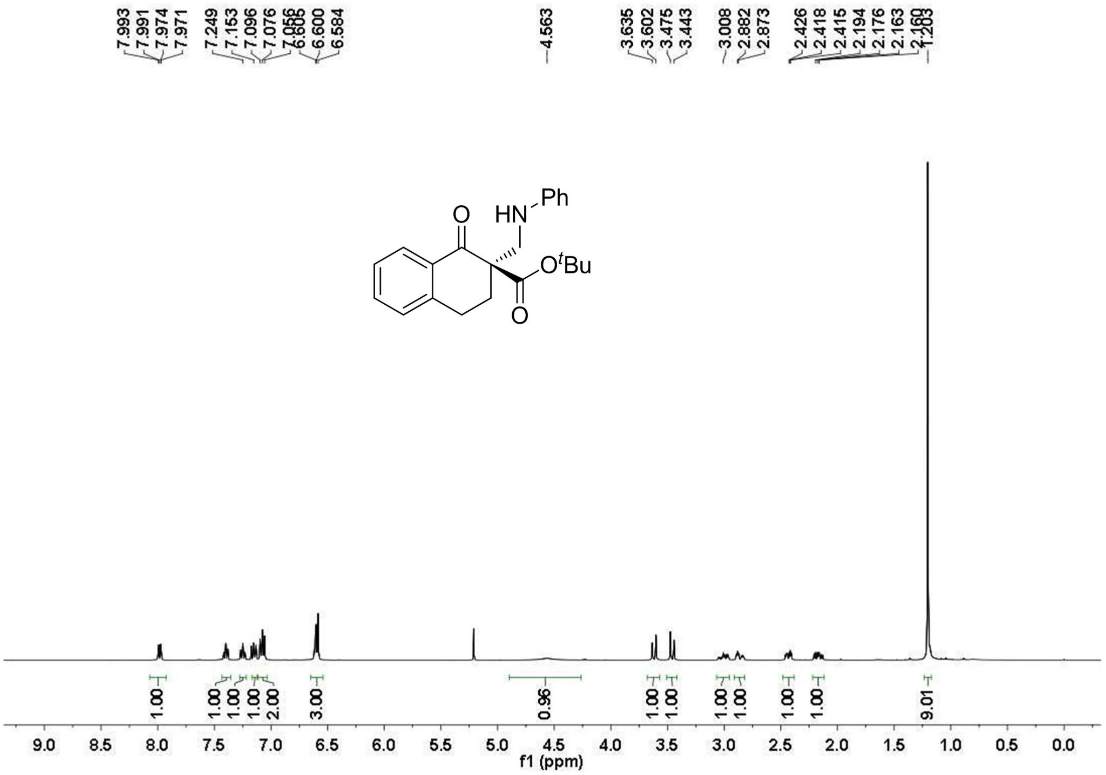

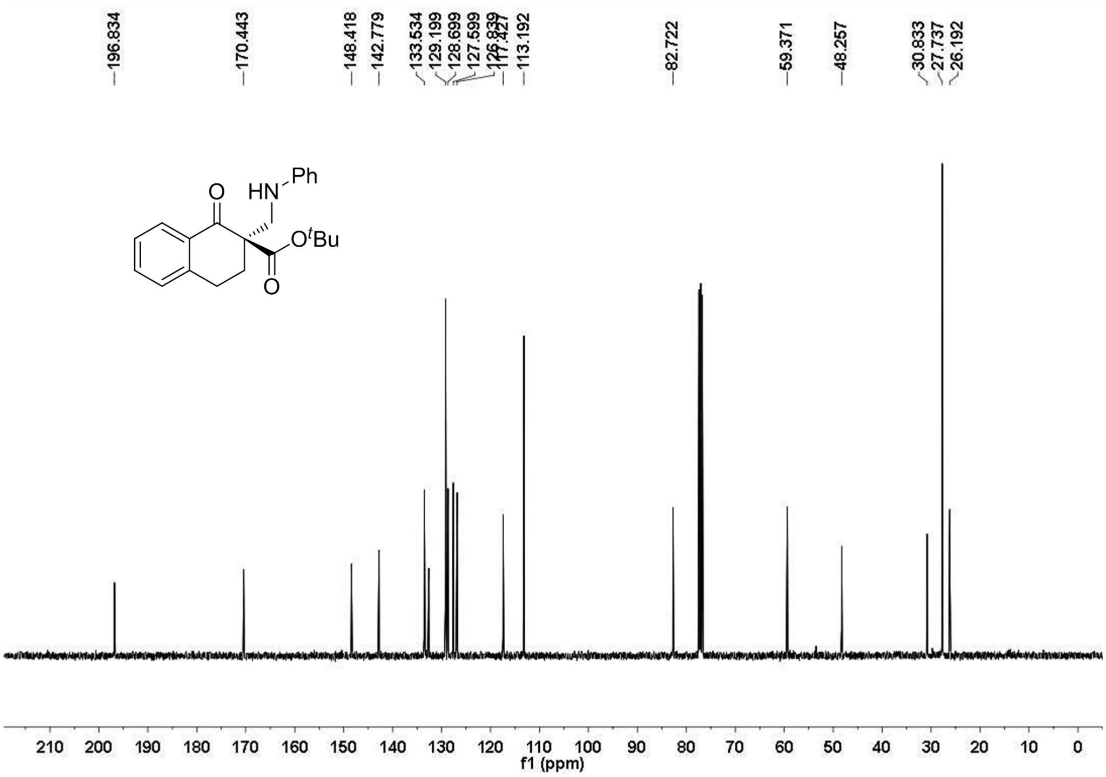

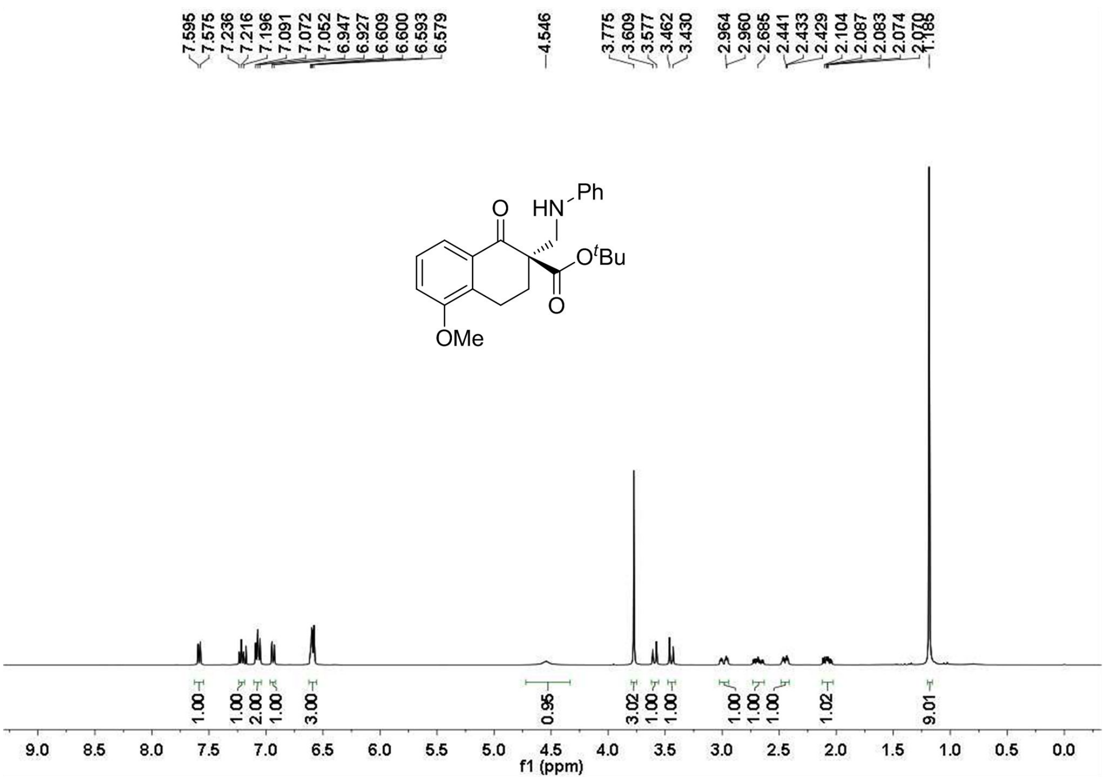
7.595
7.575
7.236
7.216
7.196
7.091
7.072
7.052
6.947
6.927
6.609
6.600
6.593
6.579
4.546
3.775
3.609
3.577
3.462
3.430
2.964
2.960
2.685
2.441
2.433
2.429
2.104
2.087
2.083
2.074
2.070
1.185
O
HN
Ph
O^{t}Bu
O
OMe
1.00
1.00
2.00
1.00
3.00
0.95
3.02
1.00
1.00
1.00
1.00
1.00
1.02
9.01
9.0 8.5 8.0 7.5 7.0 6.5 6.0 5.5 5.0 4.5 4.0 3.5 3.0 2.5 2.0 1.5 1.0 0.5 0.0
f1 (ppm)

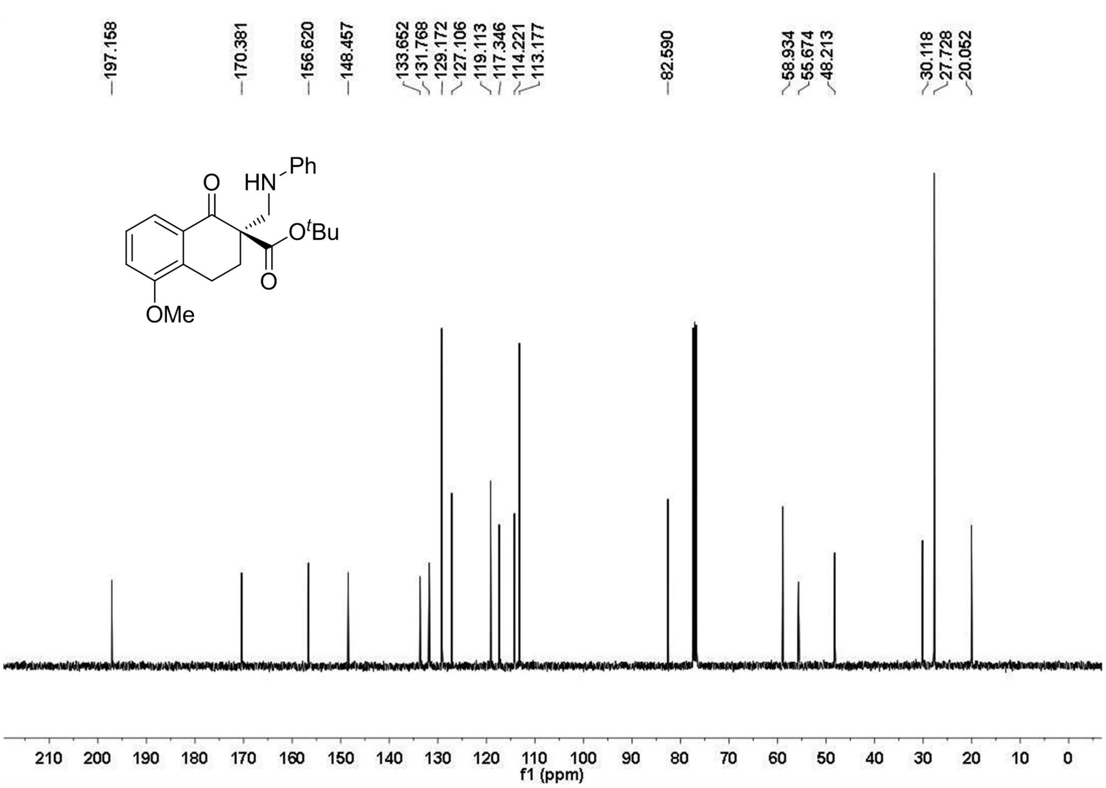
197.158
170.381
156.620
148.457
133.652
131.768
129.172
127.106
119.113
117.346
114.221
113.177
82.590
58.934
55.674
48.213
30.118
27.728
20.052
O
HN
Ph
O^{t}Bu
O
OMe
210 200 190 180 170 160 150 140 130 120 110 100 90 80 70 60 50 40 30 20 10 0
f1 (ppm)

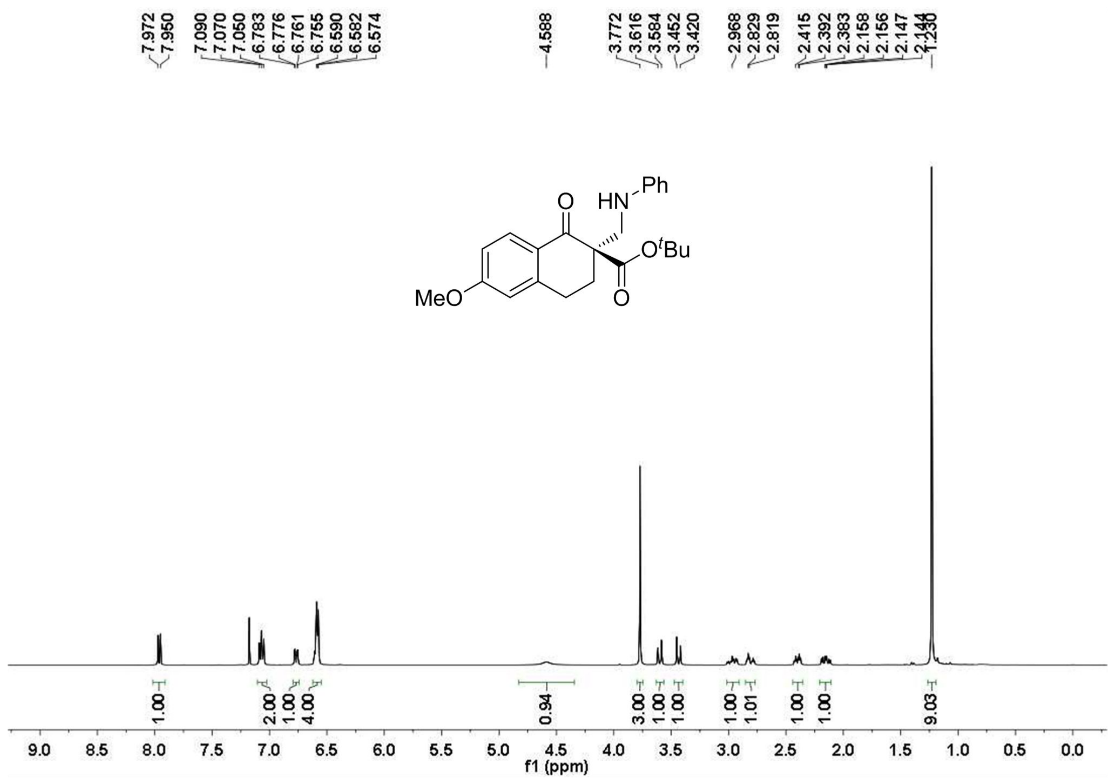
7.972
7.950
7.090
7.070
7.050
6.783
6.776
6.761
6.755
6.590
6.582
6.574
4.588
3.772
3.616
3.584
3.452
3.420
2.968
2.829
2.819
2.415
2.392
2.383
2.158
2.156
2.147
2.144
1.230
O
HN
Ph
O^{t}Bu
MeO
O
1.00
2.00
1.00
4.00
0.94
3.00
1.00
1.00
1.00
1.01
1.00
1.00
9.03
9.0 8.5 8.0 7.5 7.0 6.5 6.0 5.5 5.0 4.5 4.0 3.5 3.0 2.5 2.0 1.5 1.0 0.5 0.0
f1 (ppm)

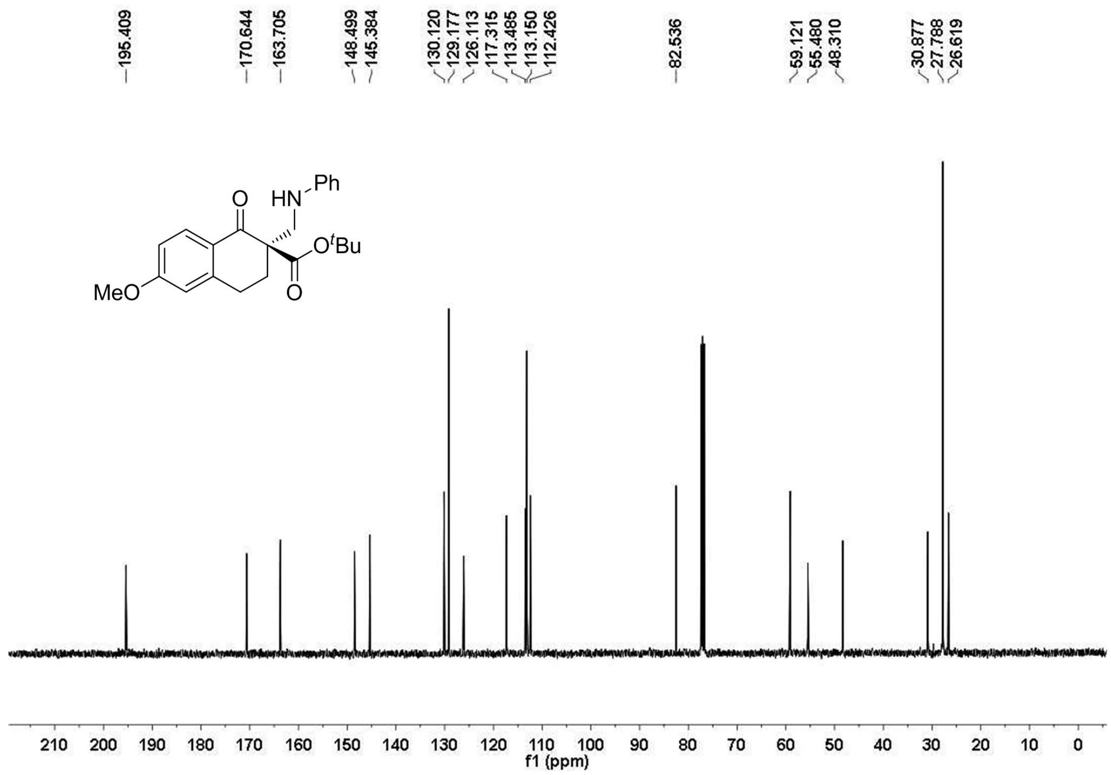
195.409
170.644
163.705
148.499
145.384
130.120
129.177
126.113
117.315
113.485
113.150
112.426
82.536
59.121
55.480
48.310
30.877
27.788
26.619
O
HN
Ph
O^{t}Bu
MeO
O
210 200 190 180 170 160 150 140 130 120 110 100 90 80 70 60 50 40 30 20 10 0
f1 (ppm)

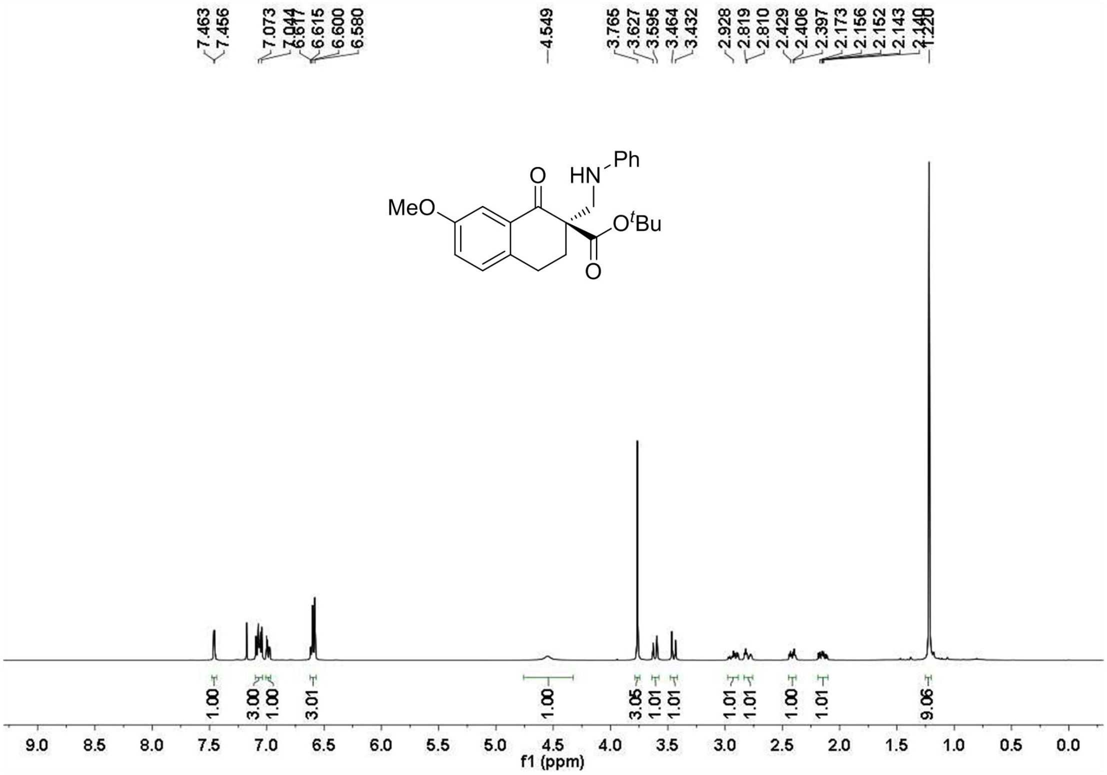
7.463
7.456
7.073
7.044
6.617
6.615
6.600
6.580
4.549
3.765
3.627
3.595
3.464
3.432
2.928
2.819
2.810
2.429
2.406
2.397
2.173
2.156
2.152
2.143
2.140
1.220
O
HN
Ph
MeO
O^{t}Bu
O
1.00
3.00
1.00
3.01
1.00
3.05
1.01
1.01
1.01
1.01
1.00
1.01
9.06
f1 (ppm)

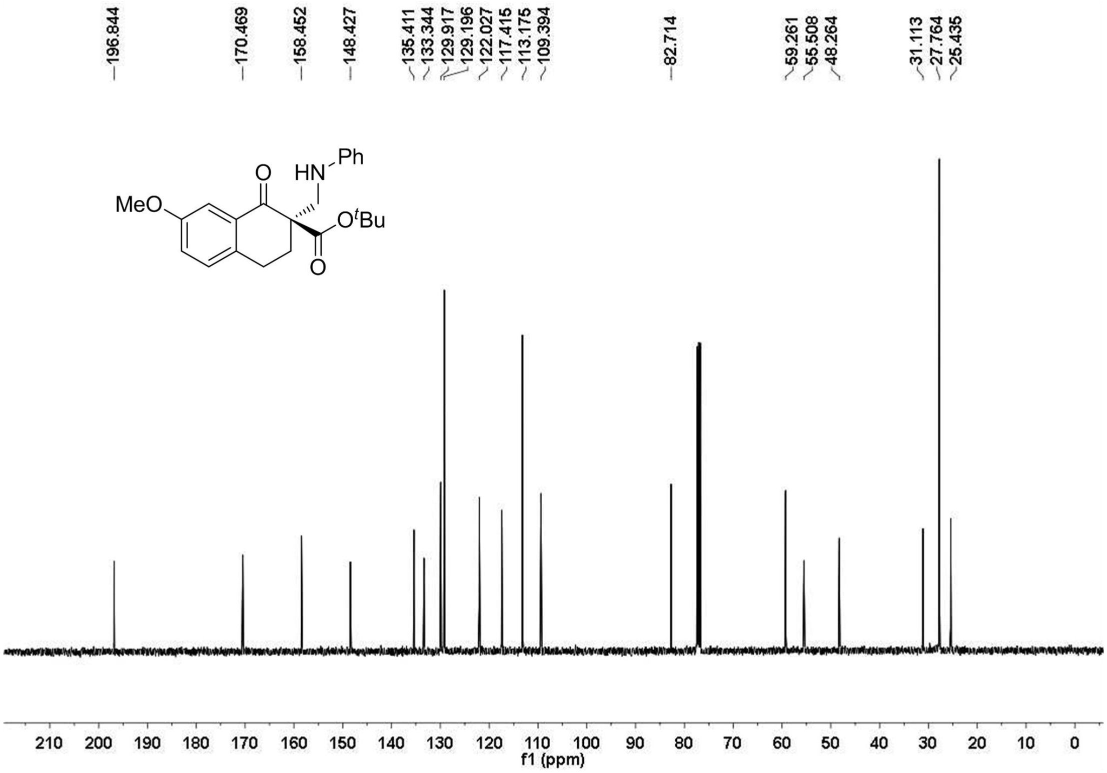
196.844
170.469
158.452
148.427
135.411
133.344
129.917
129.196
122.027
117.415
113.175
109.394
82.714
59.261
55.508
48.264
31.113
27.764
25.435
O
HN
Ph
MeO
O^{t}Bu
O
f1 (ppm)

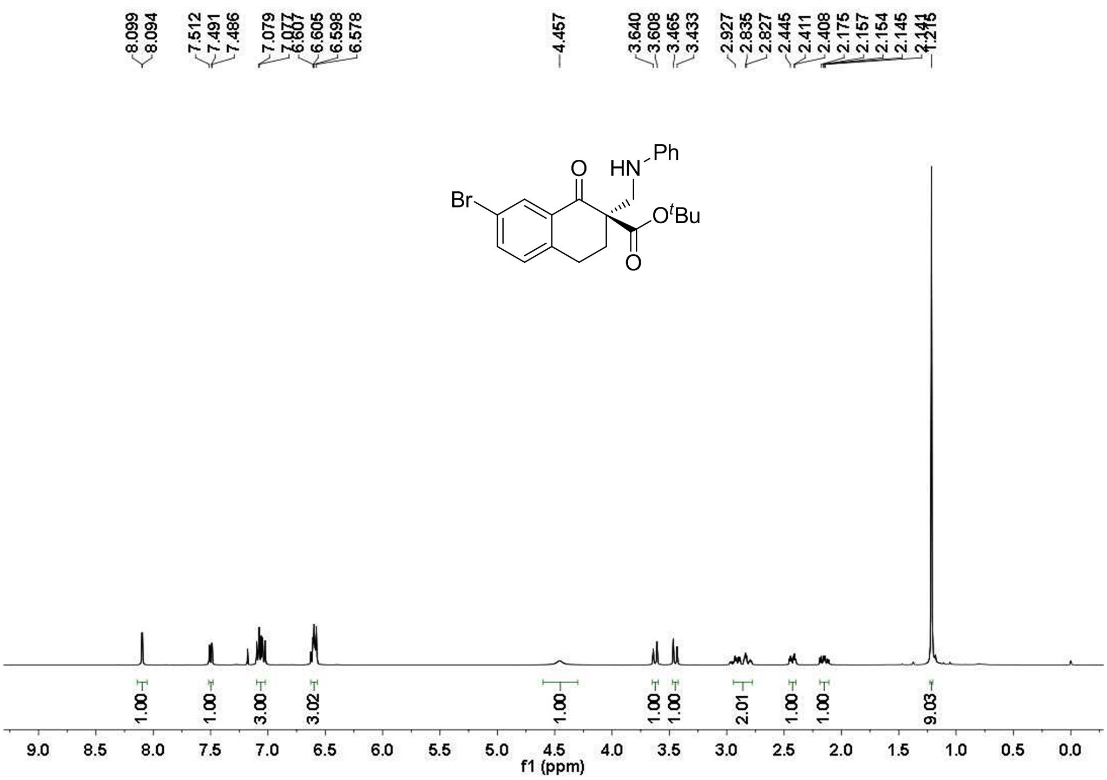
8.099
8.094
7.512
7.491
7.486
7.079
7.077
6.607
6.605
6.598
6.578
4.457
3.640
3.608
3.465
3.433
2.927
2.835
2.827
2.445
2.411
2.408
2.175
2.157
2.154
2.145
2.141
1.215
1.00
1.00
3.00
3.02
1.00
1.00
1.00
2.01
1.00
1.00
9.03
f1 (ppm)

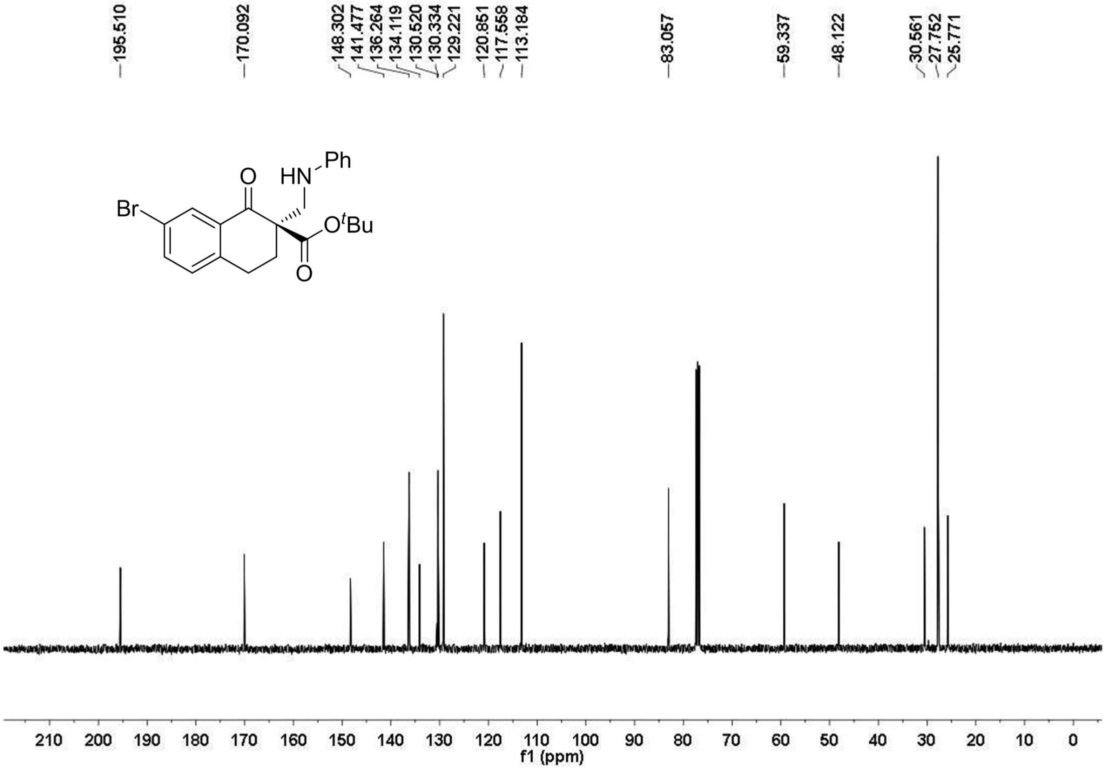
195.510
170.092
148.302
141.477
136.264
134.119
130.520
130.334
129.221
120.851
117.558
113.184
83.057
59.337
48.122
30.561
27.752
25.771
f1 (ppm)

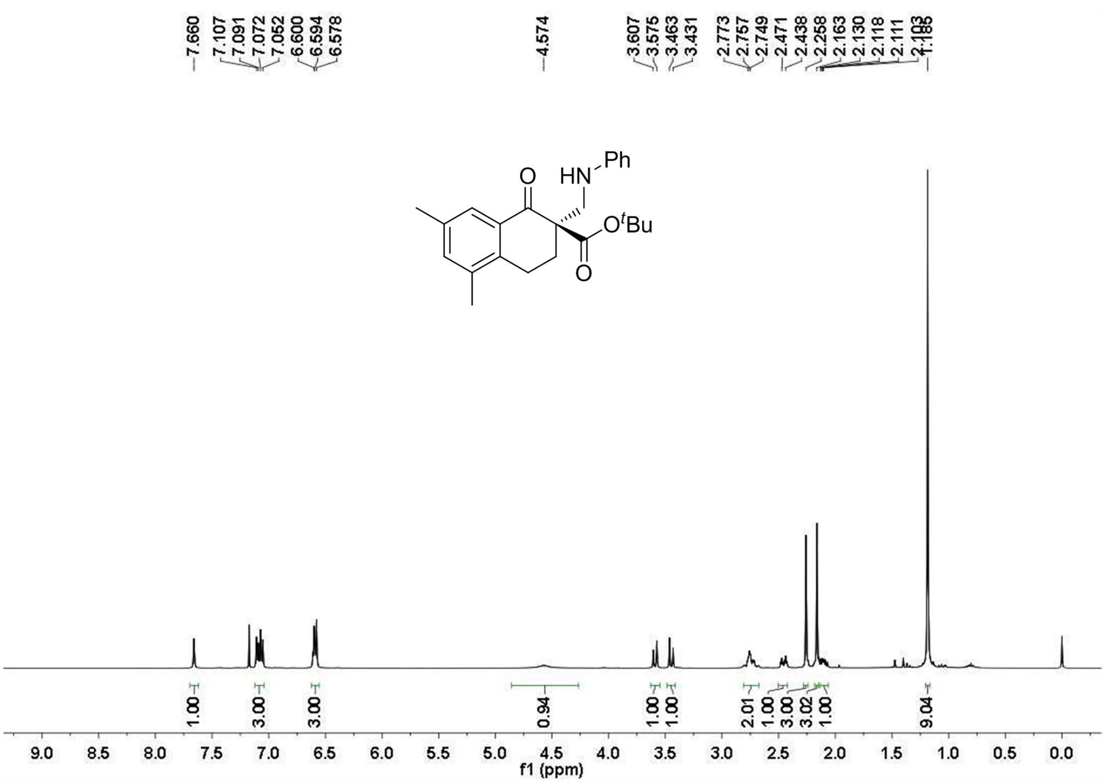
7.660
7.107
7.091
7.072
7.052
6.600
6.594
6.578
4.574
3.607
3.575
3.463
3.431
2.773
2.757
2.749
2.471
2.438
2.258
2.163
2.130
2.118
2.111
HN
Ph
O
O
O^{t}Bu
1.00
3.00
3.00
0.94
1.00
1.00
2.01
1.00
3.00
3.02
1.00
9.04
f1 (ppm)

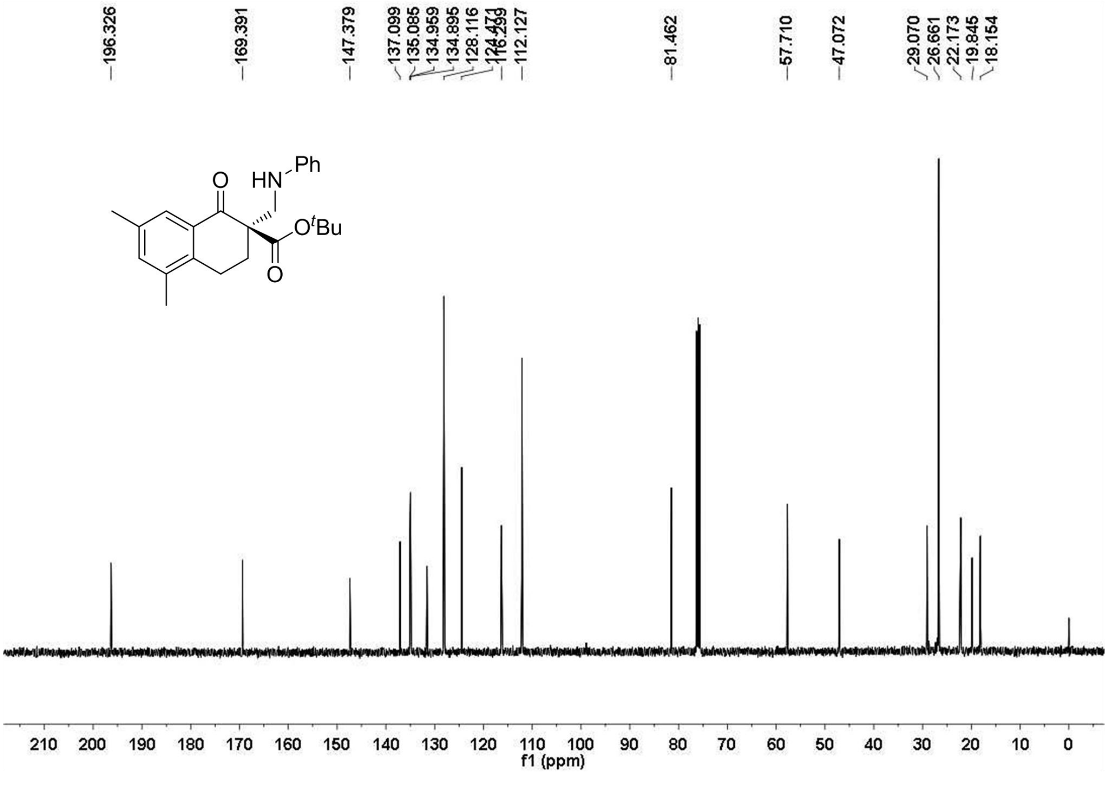
196.326
169.391
147.379
137.099
135.085
134.959
134.895
128.116
112.127
81.462
57.710
47.072
29.070
26.661
22.173
19.845
18.154
HN
Ph
O
O
O^{t}Bu
f1 (ppm)

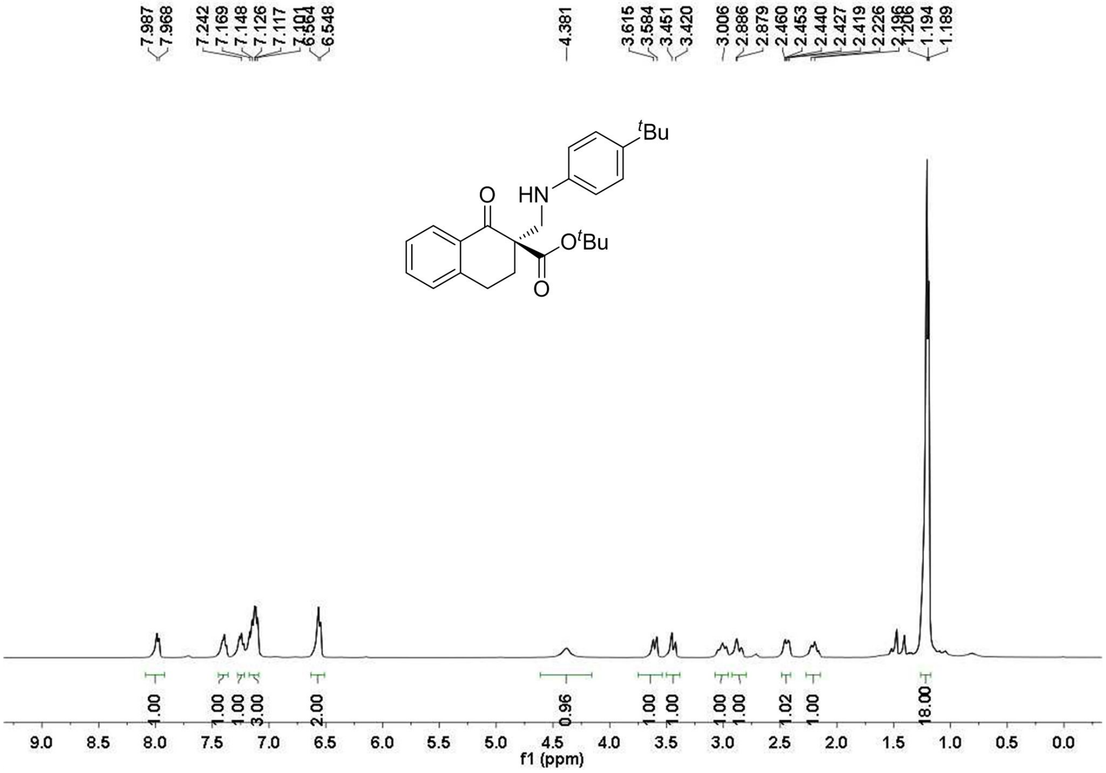

7.987
7.968
7.242
7.169
7.148
7.126
7.117
7.101
6.561
6.548
4.381
3.615
3.584
3.451
3.420
3.006
2.886
2.879
2.460
2.453
2.440
2.427
2.419
2.226
2.196
1.206
1.194
1.189
tBu
O
HN
OtBu
O
1.00
1.00
1.00
3.00
2.00
0.96
1.00
1.00
1.00
1.00
1.02
1.00
18.00
9.0 8.5 8.0 7.5 7.0 6.5 6.0 5.5 5.0 4.5 4.0 3.5 3.0 2.5 2.0 1.5 1.0 0.5 0.0
f1 (ppm)

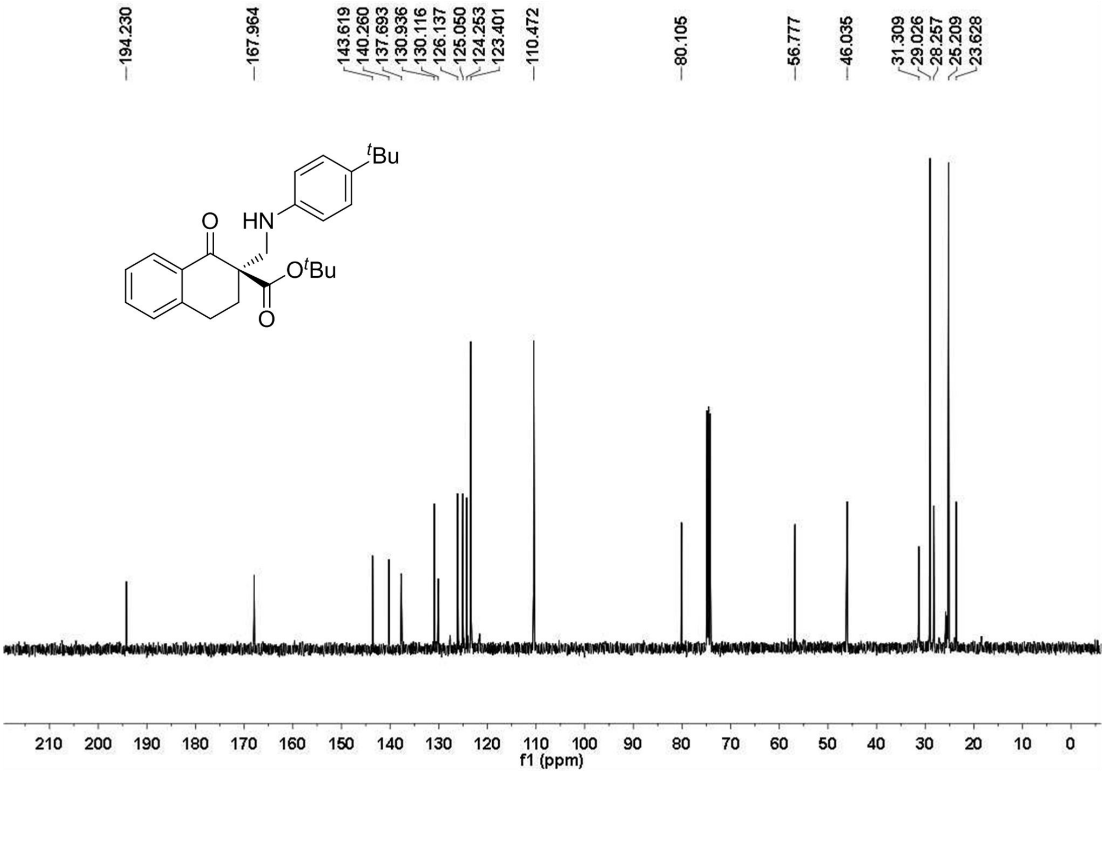

194.230
167.964
143.619
140.260
137.693
130.936
130.116
126.137
125.050
124.253
123.401
110.472
80.105
56.777
46.035
31.309
29.026
28.257
25.209
23.628
tBu
O
HN
OtBu
O
210 200 190 180 170 160 150 140 130 120 110 100 90 80 70 60 50 40 30 20 10 0
f1 (ppm)

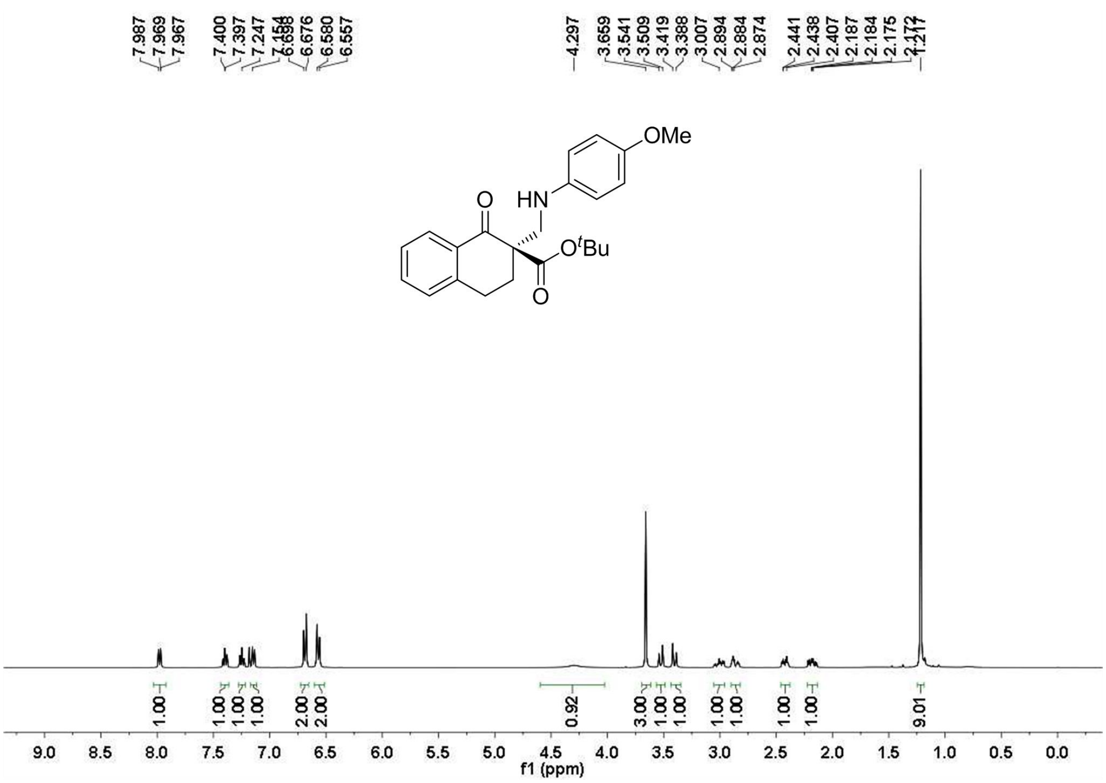

7.987
7.969
7.967
7.400
7.397
7.247
6.676
6.580
6.557
4.297
3.659
3.541
3.509
3.419
3.388
3.007
2.894
2.884
2.874
2.441
2.438
2.407
2.187
2.184
2.175
OMe
HN
O
O^{t}Bu
O
1.00
1.00
1.00
1.00
2.00
2.00
0.92
3.00
1.00
1.00
1.00
1.00
1.00
1.00
9.01
f1 (ppm)

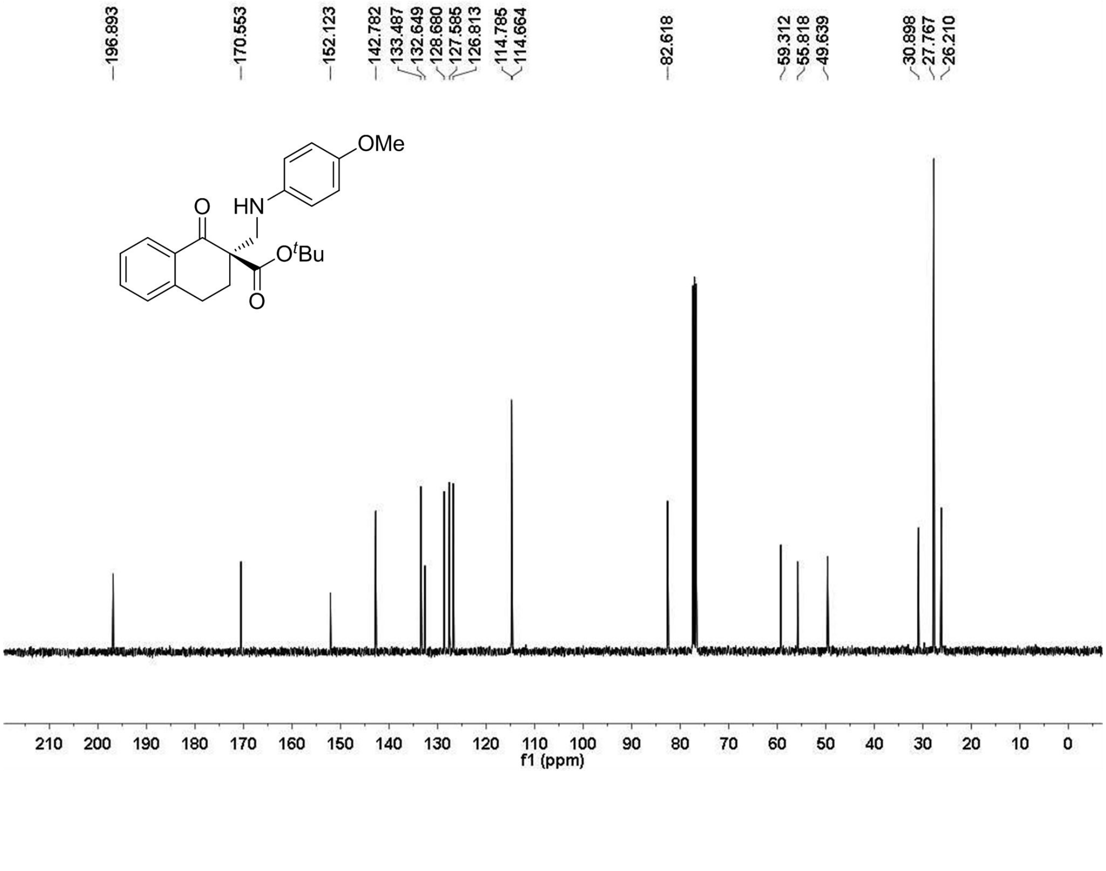

196.893
170.553
152.123
142.782
133.487
132.649
128.680
127.585
126.813
114.785
114.664
82.618
59.312
55.818
49.639
30.898
27.767
26.210
OMe
HN
O
O^{t}Bu
O
f1 (ppm)

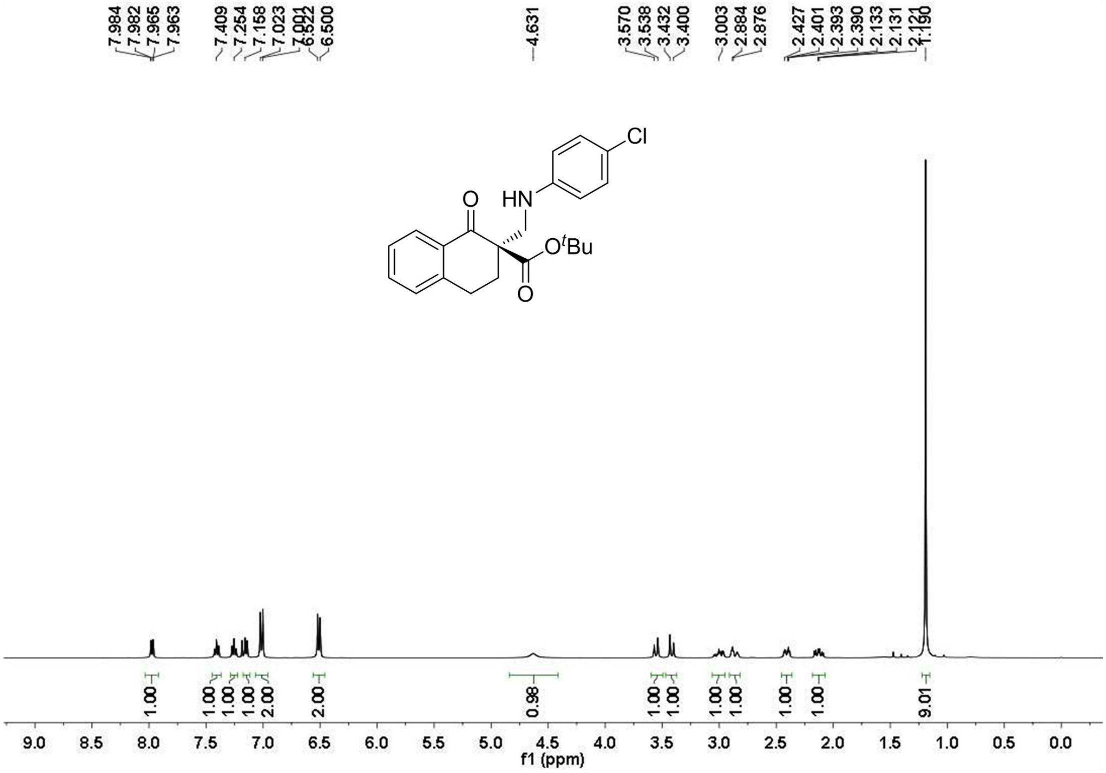
7.984
7.982
7.965
7.963
7.409
7.254
7.158
7.023
7.001
6.522
6.500
4.631
3.570
3.538
3.432
3.400
3.003
2.884
2.876
2.427
2.401
2.393
2.390
2.133
2.131
2.121
1.190
Cl
HN
O
OtBu
O
1.00
1.00
1.00
1.00
2.00
2.00
0.98
1.00
1.00
1.00
1.00
1.00
1.00
9.01
9.0 8.5 8.0 7.5 7.0 6.5 6.0 5.5 5.0 4.5 4.0 3.5 3.0 2.5 2.0 1.5 1.0 0.5 0.0
f1 (ppm)

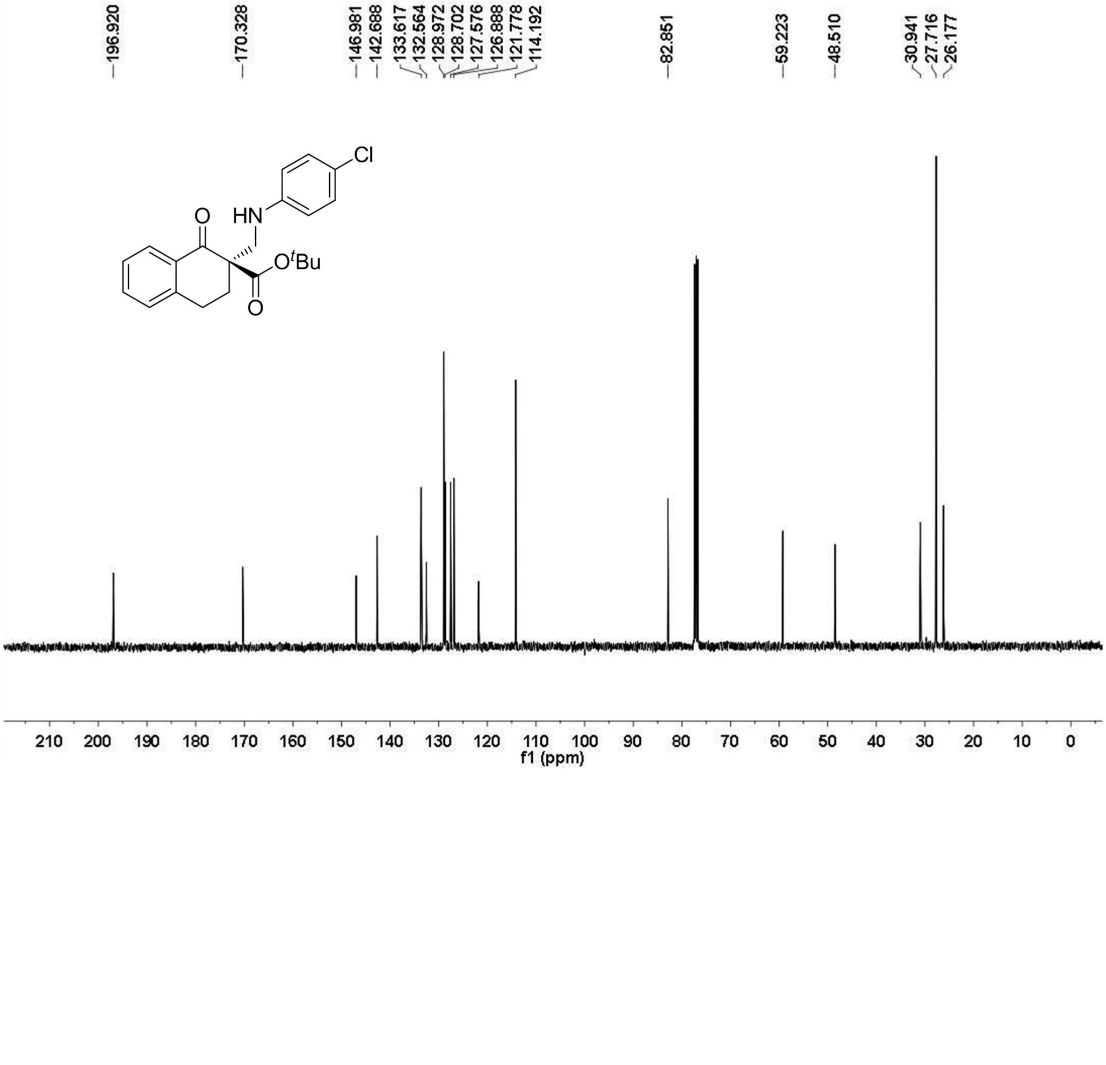
196.920
170.328
146.981
142.688
133.617
132.564
128.972
128.702
127.576
126.888
121.778
114.192
82.851
59.223
48.510
30.941
27.716
26.177
Cl
HN
O
OtBu
O
210 200 190 180 170 160 150 140 130 120 110 100 90 80 70 60 50 40 30 20 10 0
f1 (ppm)

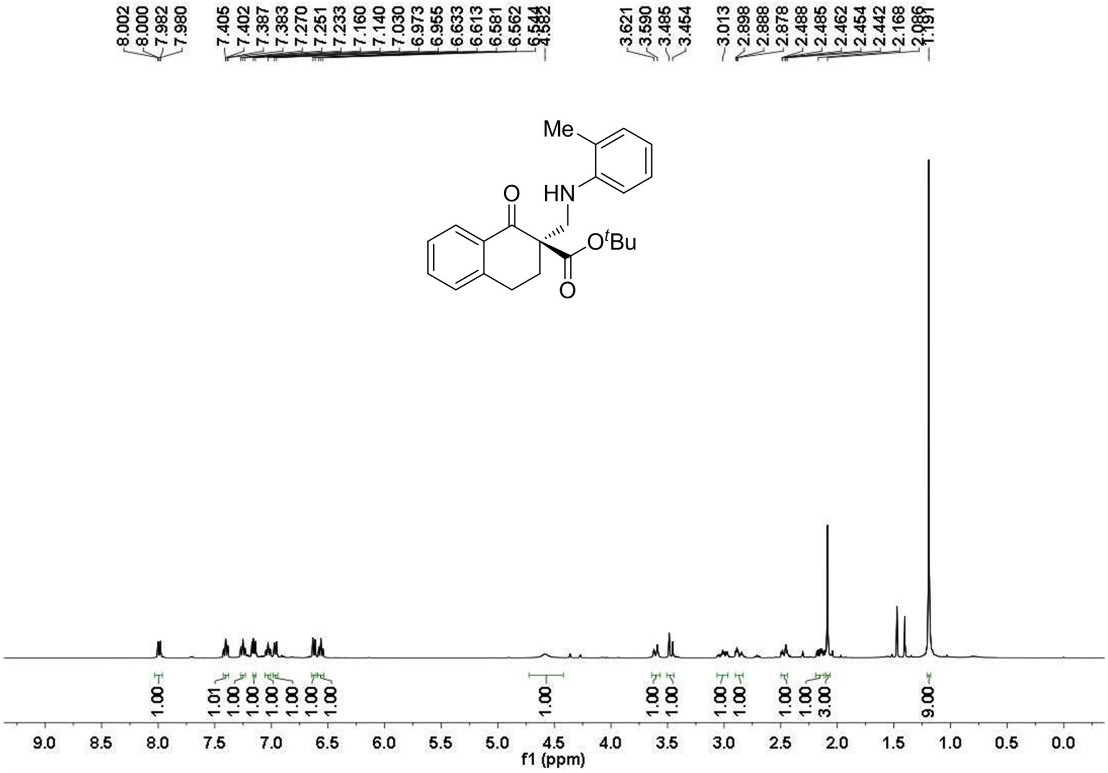
8.002
8.000
7.982
7.980
7.405
7.402
7.387
7.383
7.270
7.251
7.233
7.160
7.140
7.030
6.973
6.955
6.633
6.613
6.581
6.562
3.621
3.590
3.485
3.454
3.013
2.898
2.888
2.878
2.488
2.485
2.462
2.454
2.442
2.168
1.191
Me
HN
O
O^{t}Bu
O
1.00
1.01
1.00
1.00
1.00
1.00
1.00
1.00
1.00
1.00
1.00
1.00
1.00
1.00
1.00
3.00
9.00
9.0
8.5
8.0
7.5
7.0
6.5
6.0
5.5
5.0
4.5
4.0
3.5
3.0
2.5
2.0
1.5
1.0
0.5
0.0
f1 (ppm)

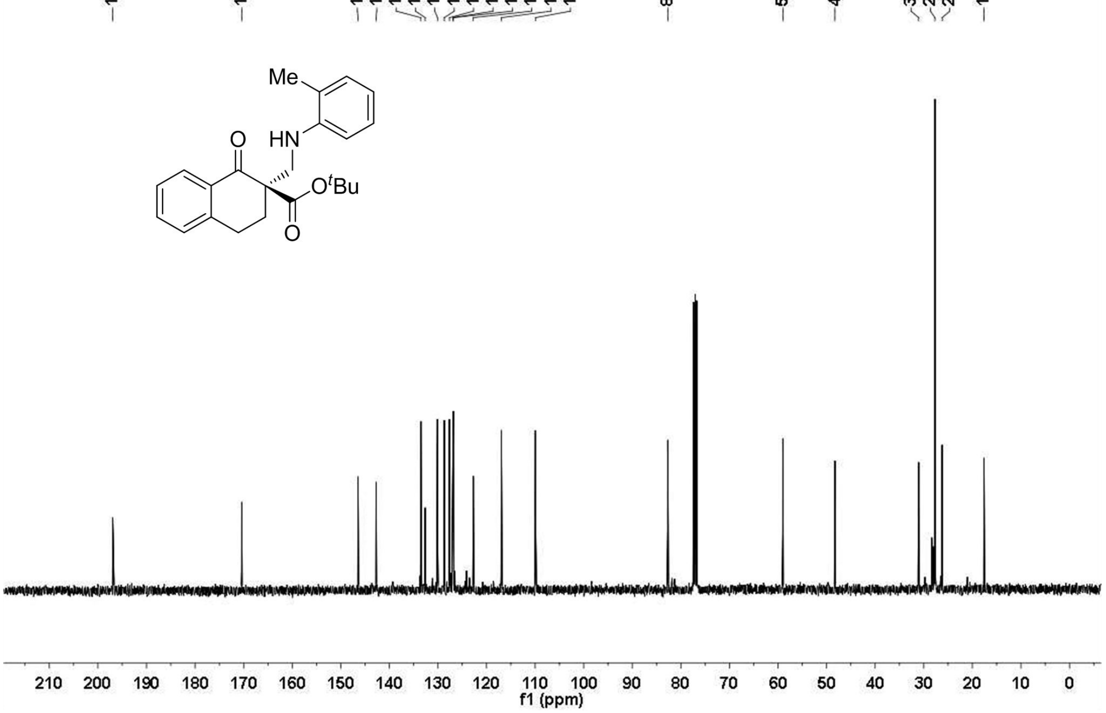
196.952
170.391
146.461
142.731
133.526
132.636
130.093
128.693
127.641
126.959
126.833
122.724
116.958
109.931
82.691
58.994
48.289
31.054
27.699
26.208
17.596
Me
HN
O
O^{t}Bu
O
210
200
190
180
170
160
150
140
130
120
110
100
90
80
70
60
50
40
30
20
10
0
f1 (ppm)

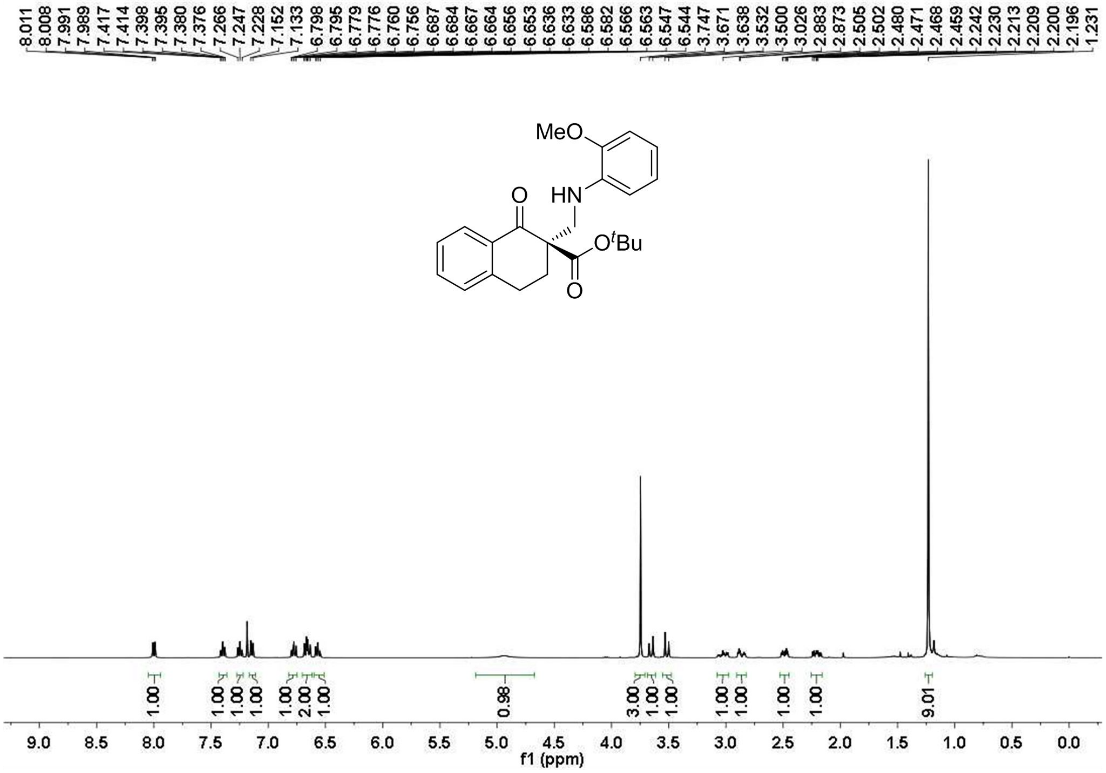

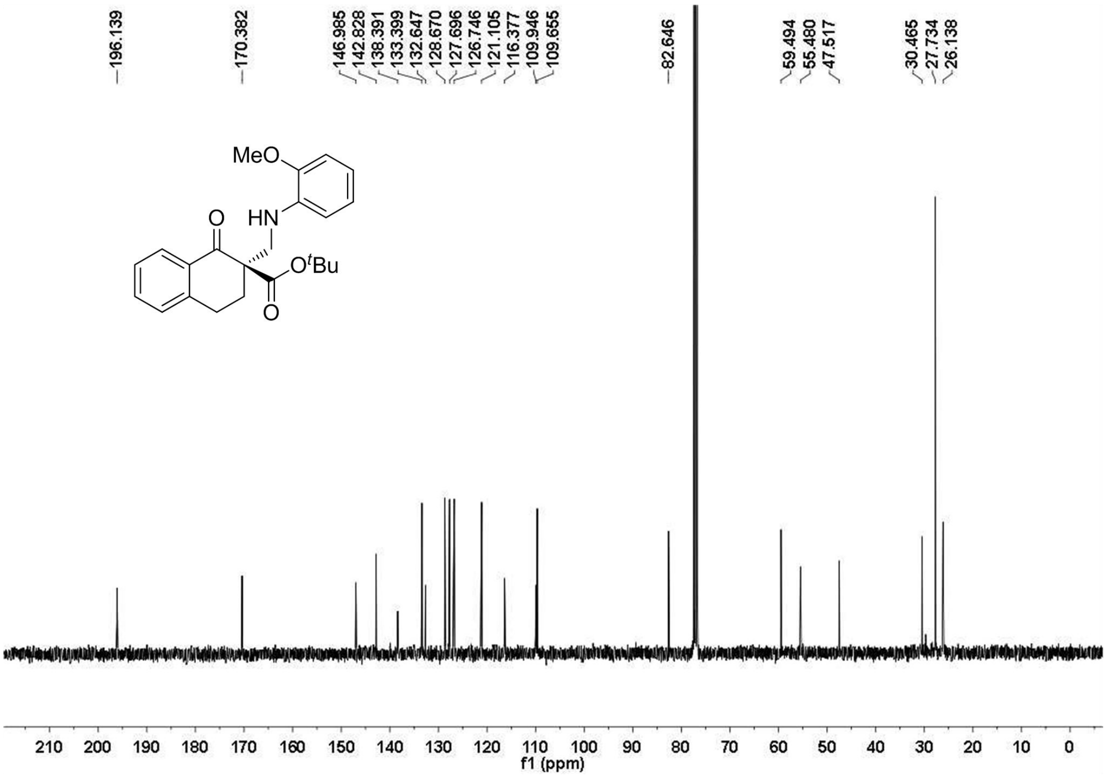

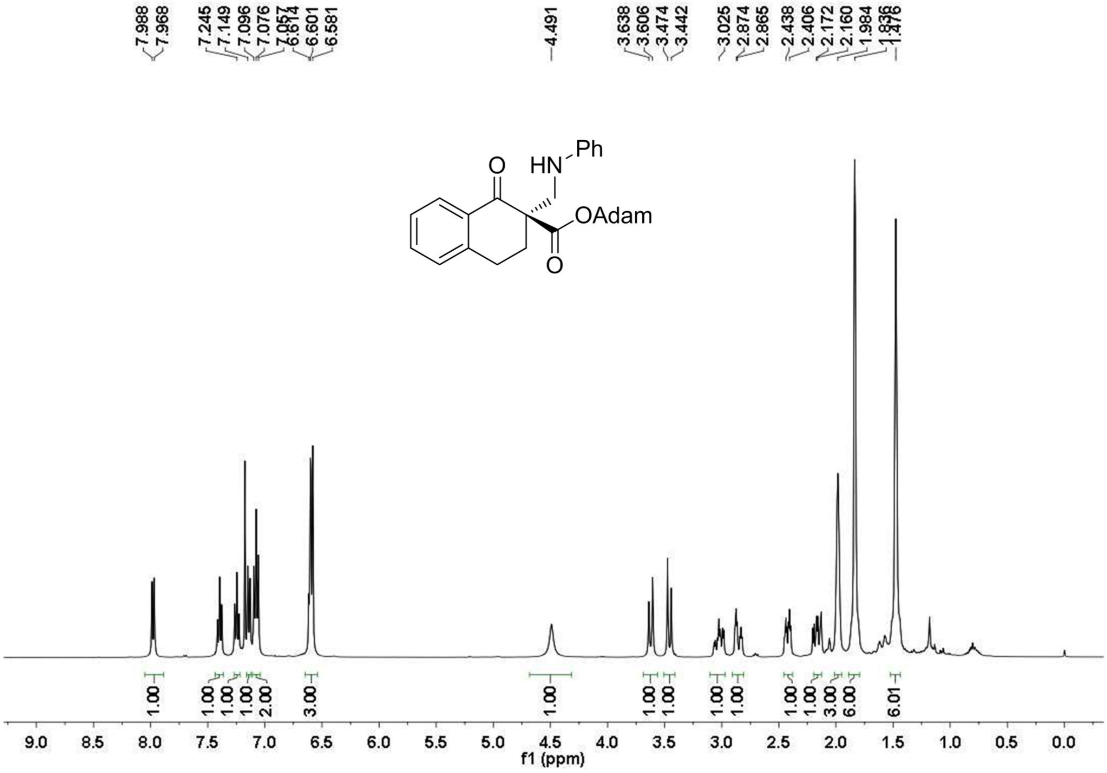

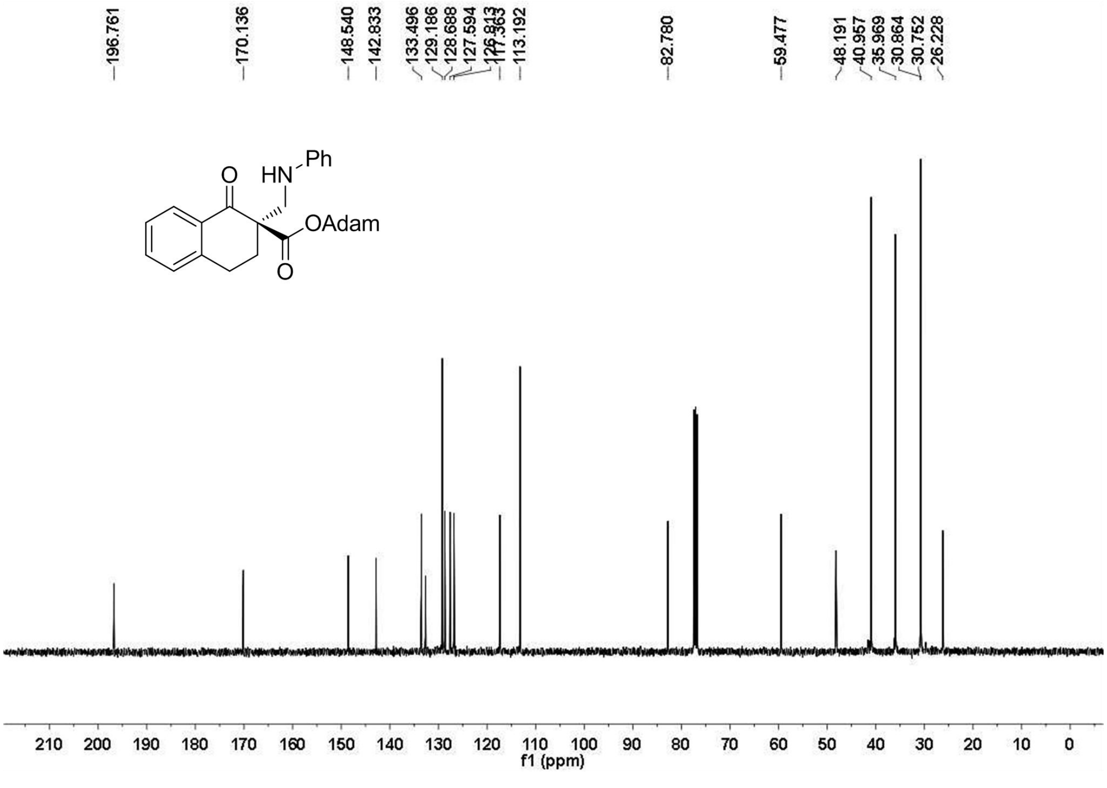

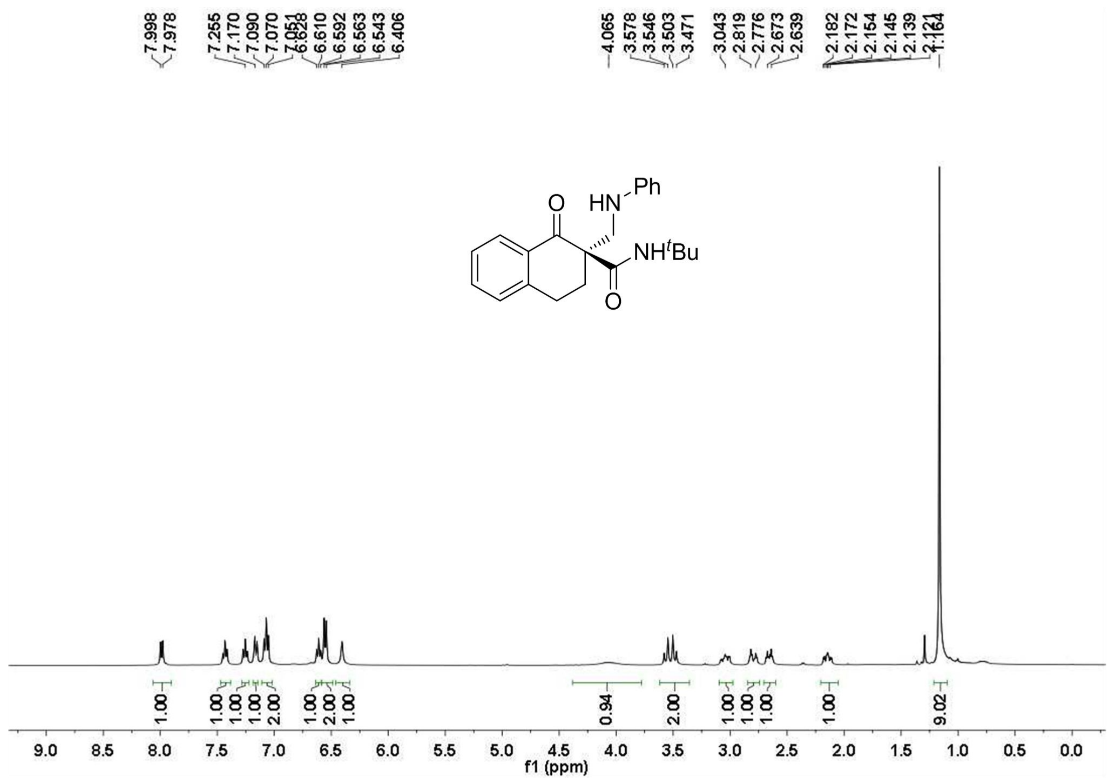
7.998
7.978
7.255
7.170
7.090
7.070
7.051
6.628
6.610
6.592
6.563
6.543
6.406
4.065
3.578
3.546
3.503
3.471
3.043
2.819
2.776
2.673
2.639
2.182
2.172
2.154
2.145
2.139
2.121
1.164
HN
Ph
O
NH^{t}Bu
O
1.00
1.00
1.00
1.00
2.00
1.00
2.00
1.00
0.94
2.00
1.00
1.00
1.00
1.00
9.02
9.0 8.5 8.0 7.5 7.0 6.5 6.0 5.5 5.0 4.5 4.0 3.5 3.0 2.5 2.0 1.5 1.0 0.5 0.0
f1 (ppm)

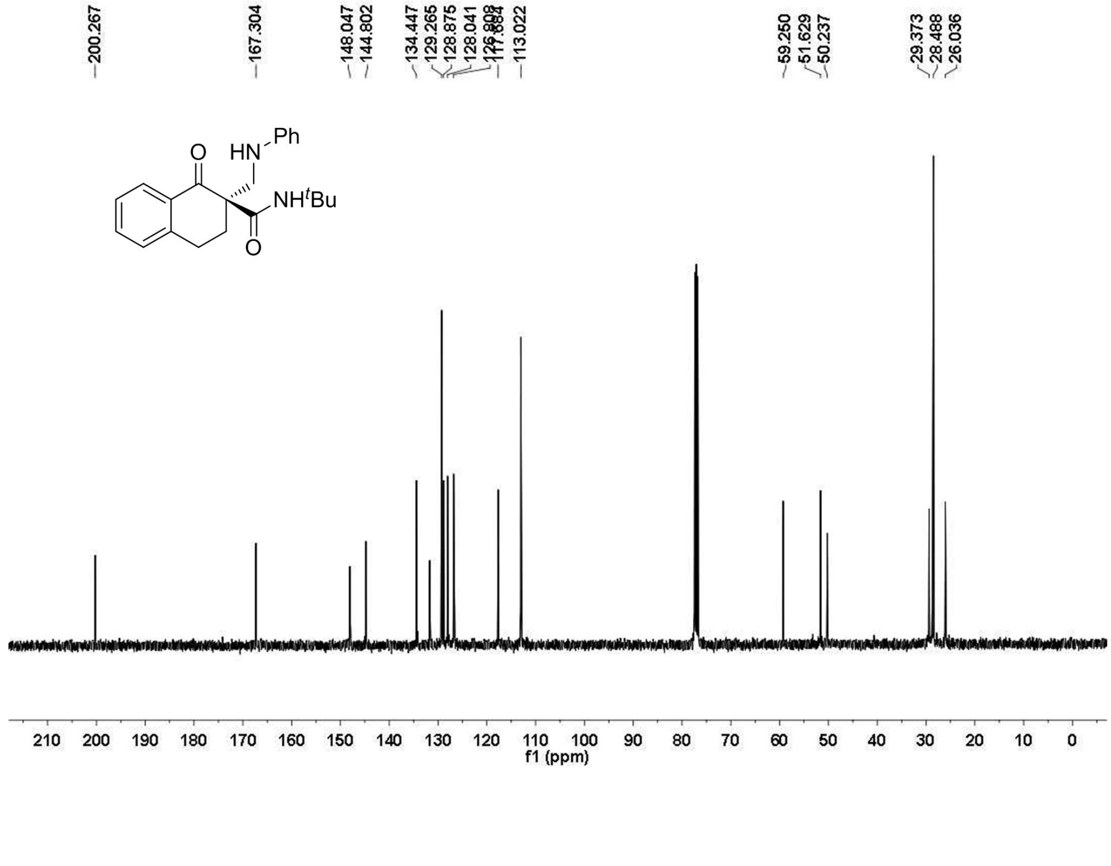
200.267
167.304
148.047
144.802
134.447
129.265
128.875
128.041
126.809
117.884
113.022
59.250
51.629
50.237
29.373
28.488
26.036
HN
Ph
O
NH^{t}Bu
O
210 200 190 180 170 160 150 140 130 120 110 100 90 80 70 60 50 40 30 20 10 0
f1 (ppm)

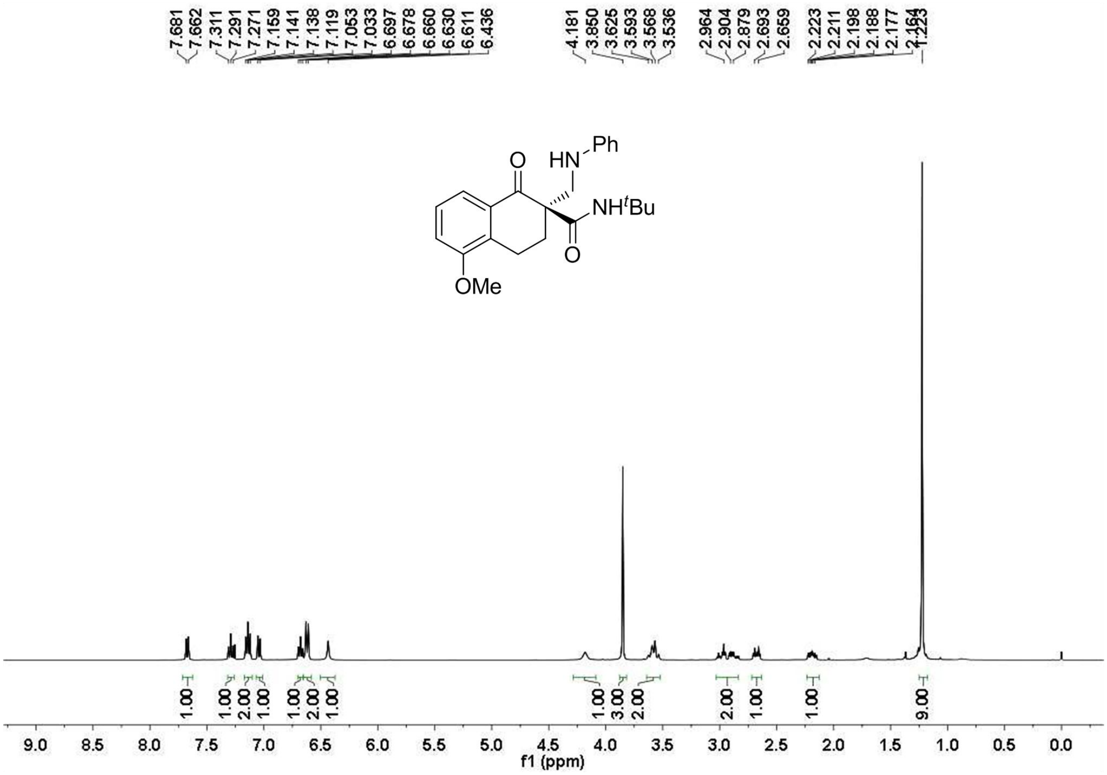

7.681
7.662
7.311
7.291
7.271
7.159
7.141
7.138
7.119
7.053
7.033
6.697
6.678
6.660
6.630
6.611
6.436
4.181
3.850
3.625
3.593
3.568
3.536
2.964
2.904
2.879
2.693
2.659
2.223
2.211
2.198
2.188
2.177
2.164
1.223
HN
Ph
O
NH^tBu
O
OMe
1.00
1.00
2.00
1.00
1.00
2.00
1.00
1.00
3.00
2.00
2.00
1.00
1.00
9.00
f1 (ppm)

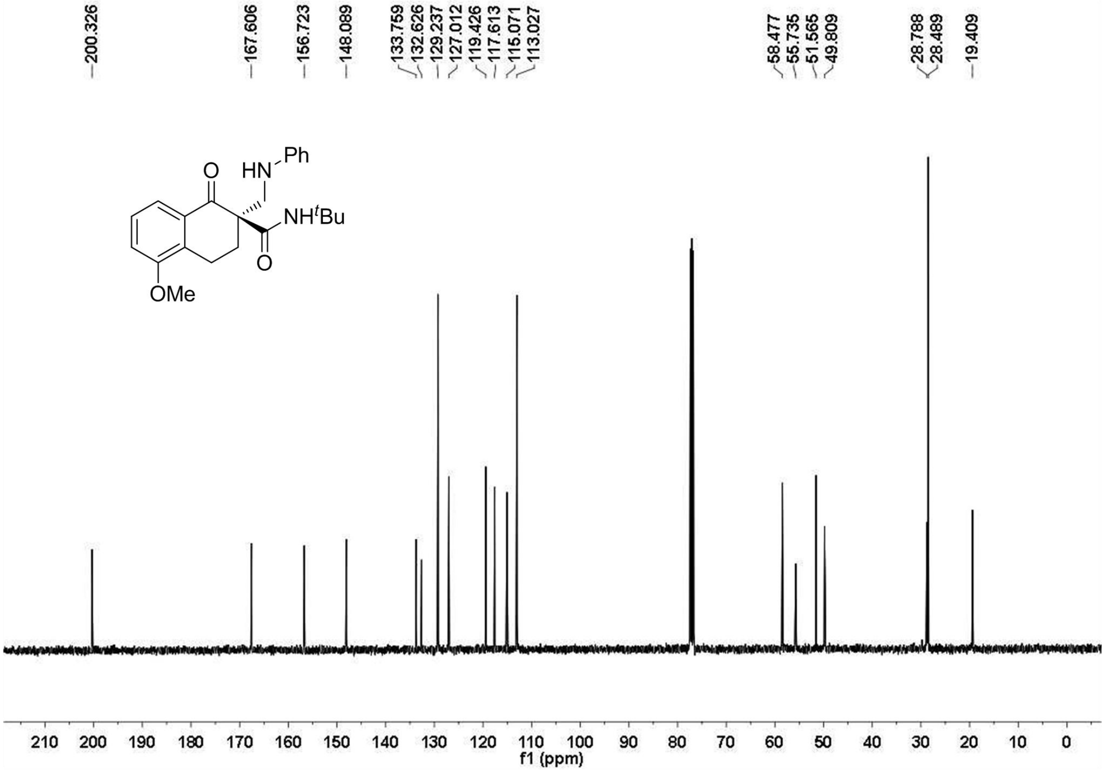

200.326
167.606
156.723
148.089
133.759
132.626
129.237
127.012
119.426
117.613
115.071
113.027
58.477
55.735
51.565
49.809
28.788
28.489
19.409
HN
Ph
O
NH^tBu
O
OMe
f1 (ppm)

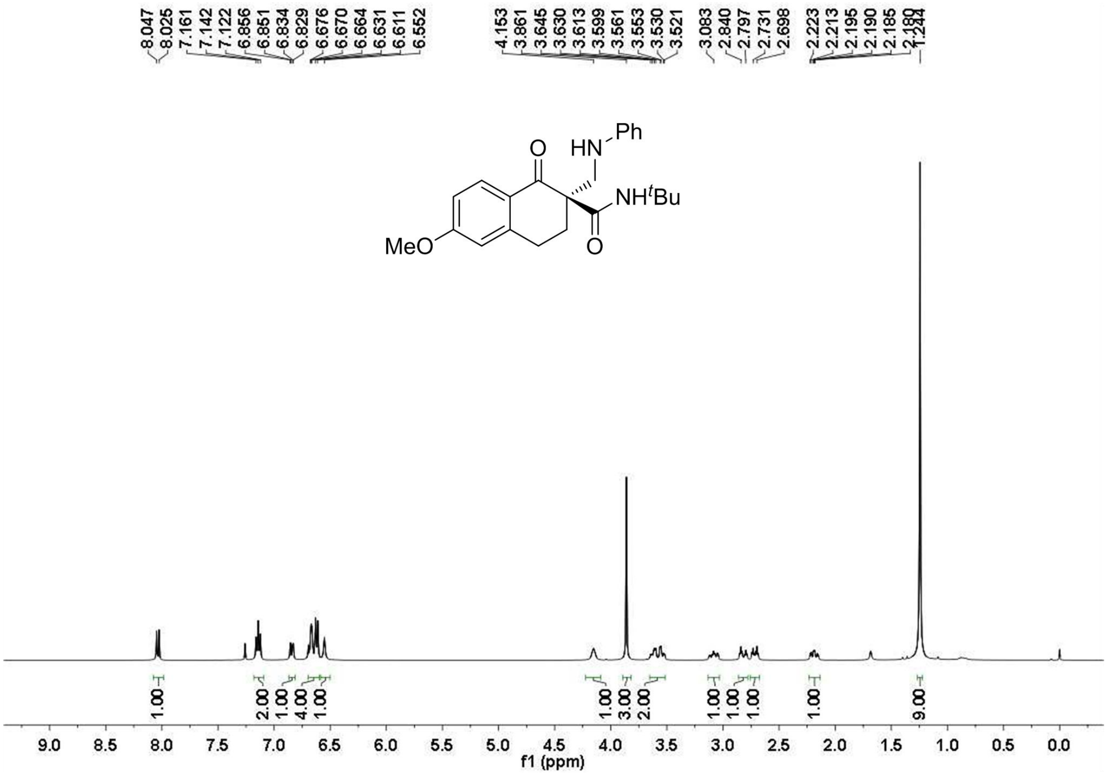

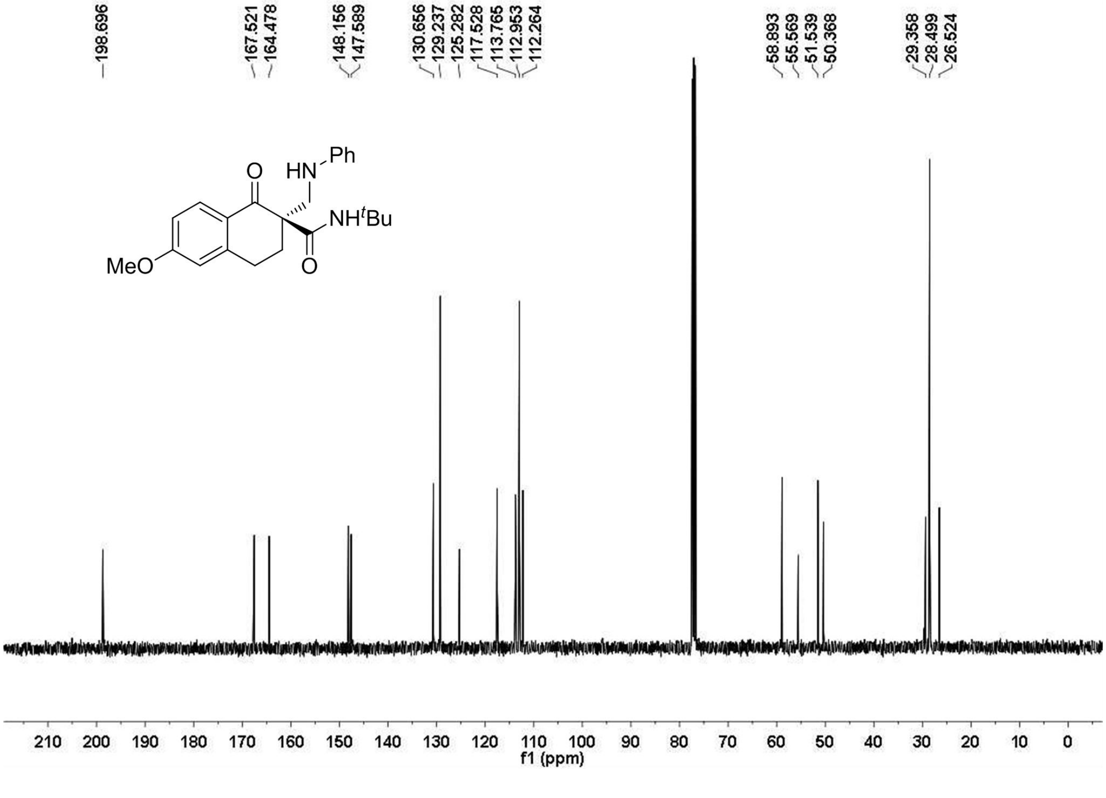

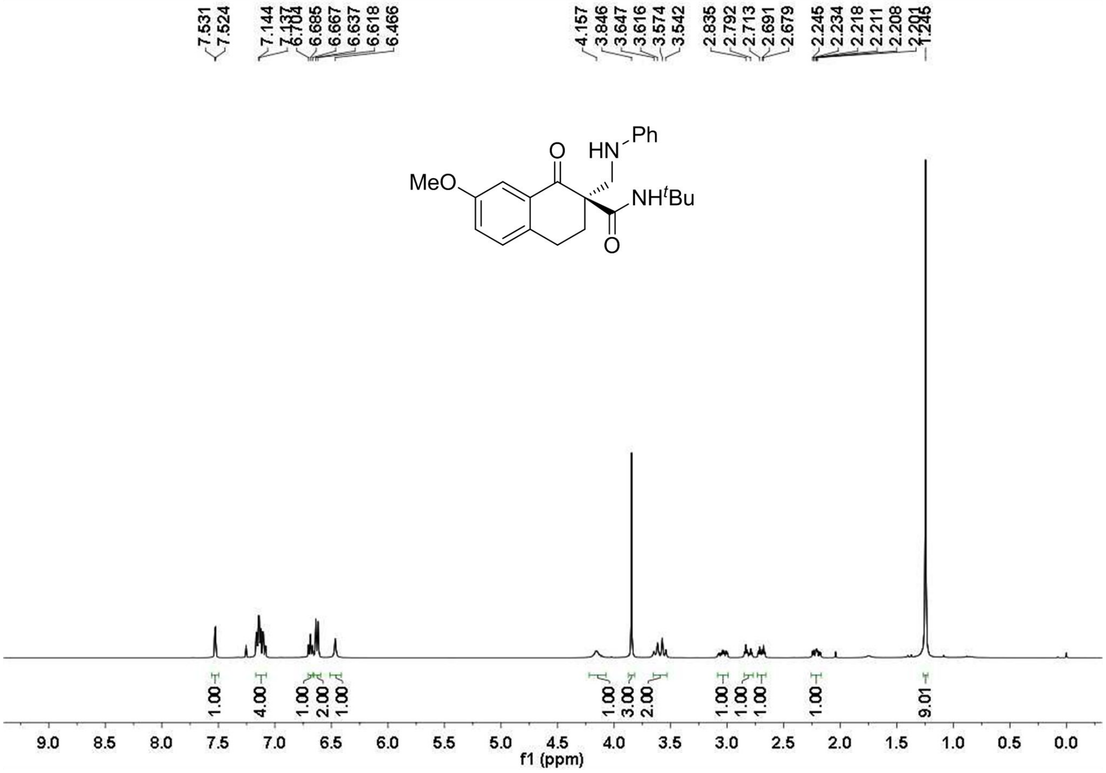

7.531
7.524
7.144
7.137
7.104
6.685
6.667
6.637
6.618
6.466
4.157
3.846
3.647
3.616
3.574
3.542
2.835
2.792
2.713
2.691
2.679
2.245
2.234
2.218
2.211
2.208
2.201
1.245
MeO
O
HN
Ph
NH^{t}Bu
O
1.00
4.00
1.00
2.00
1.00
1.00
3.00
2.00
1.00
1.00
1.00
1.00
9.01
f1 (ppm)

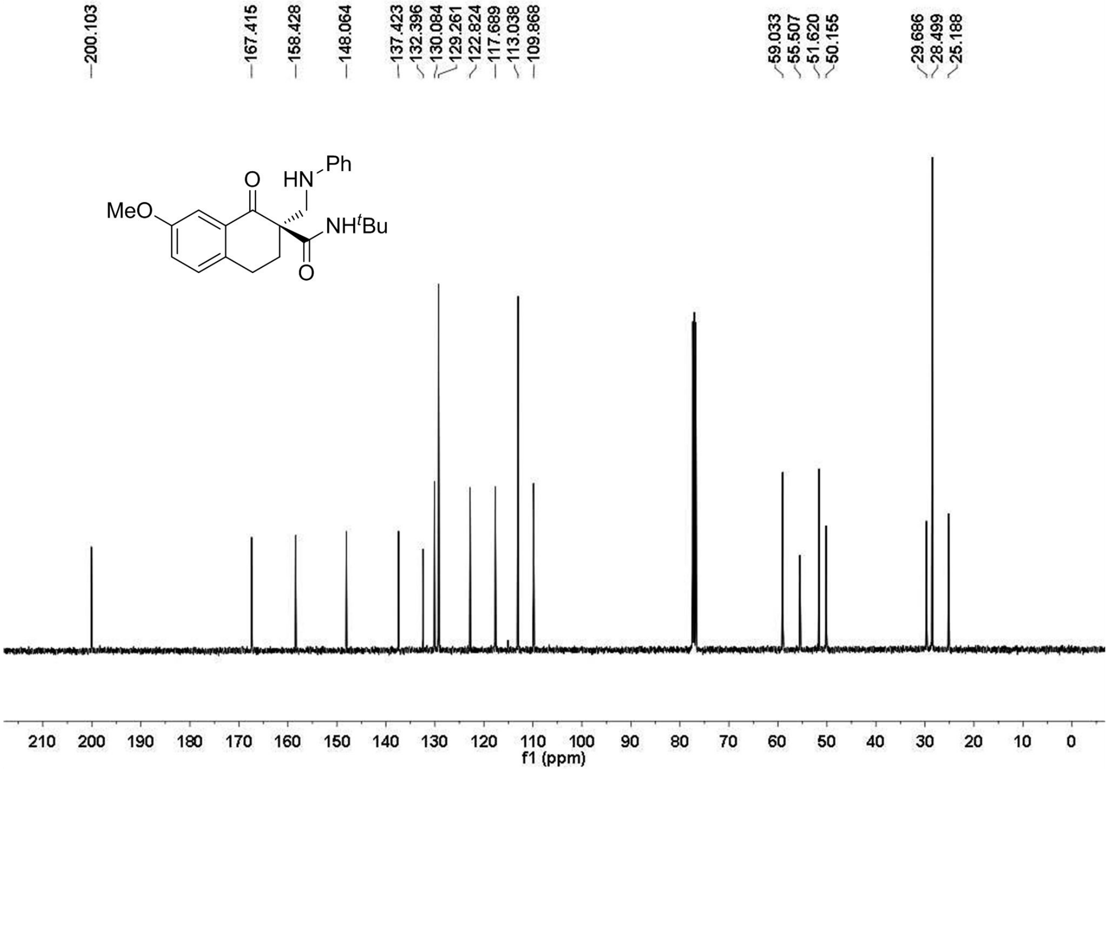

200.103
167.415
158.428
148.064
137.423
132.396
130.084
129.261
122.824
117.689
113.038
109.868
59.033
55.507
51.620
50.155
29.686
28.499
25.188
MeO
O
HN
Ph
NH^{t}Bu
O
f1 (ppm)

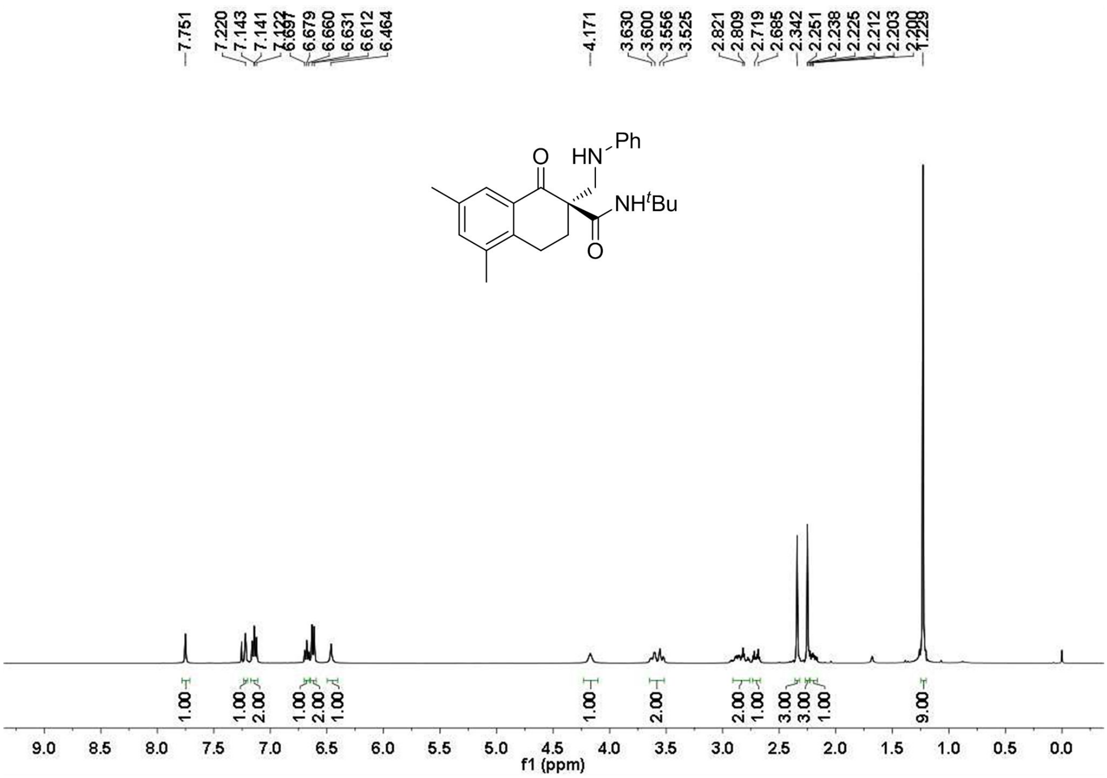
7.751
7.220
7.143
7.141
6.679
6.660
6.631
6.612
6.464
4.171
3.630
3.600
3.556
3.525
2.821
2.809
2.719
2.685
2.342
2.251
2.238
2.225
2.212
2.203
O
HN
Ph
NH^{t}Bu
O
1.00
1.00
2.00
1.00
2.00
1.00
1.00
2.00
2.00
1.00
3.00
3.00
1.00
9.00
9.0 8.5 8.0 7.5 7.0 6.5 6.0 5.5 5.0 4.5 4.0 3.5 3.0 2.5 2.0 1.5 1.0 0.5 0.0
f1 (ppm)

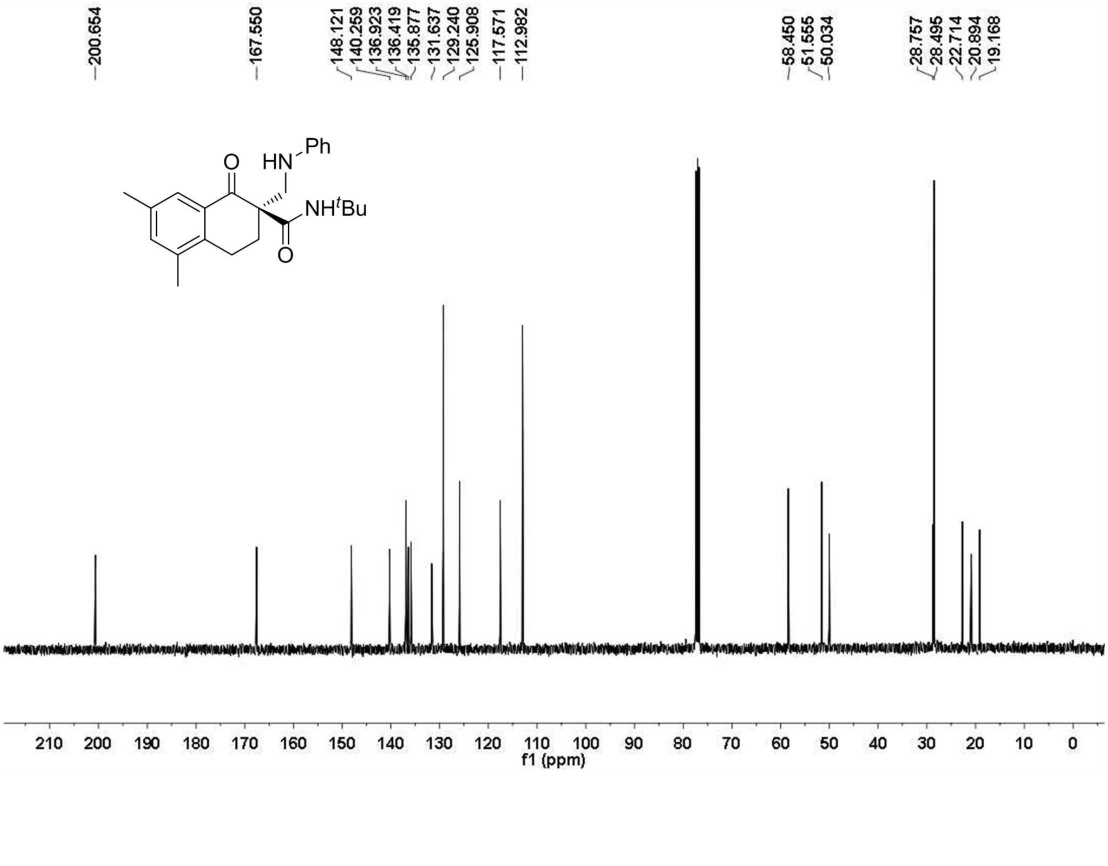
200.654
167.550
148.121
140.259
136.923
136.419
135.877
131.637
129.240
125.908
117.571
112.982
58.450
51.555
50.034
28.757
28.495
22.714
20.894
19.168
O
HN
Ph
NH^{t}Bu
O
210 200 190 180 170 160 150 140 130 120 110 100 90 80 70 60 50 40 30 20 10 0
f1 (ppm)

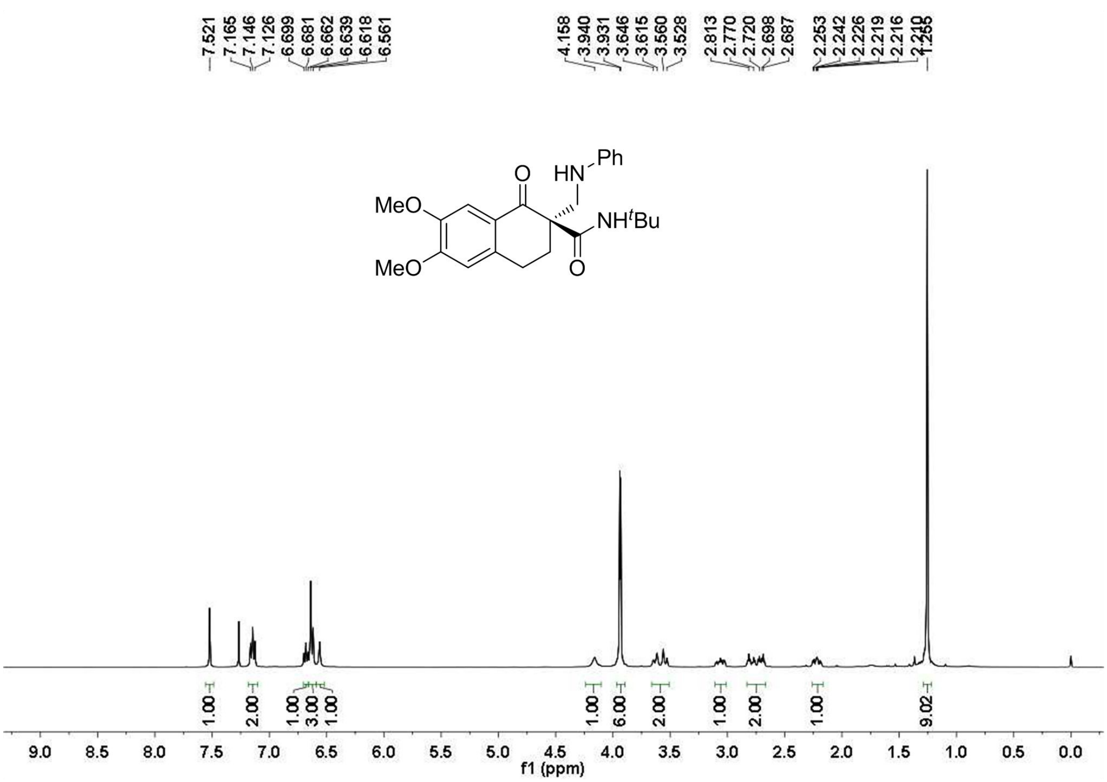

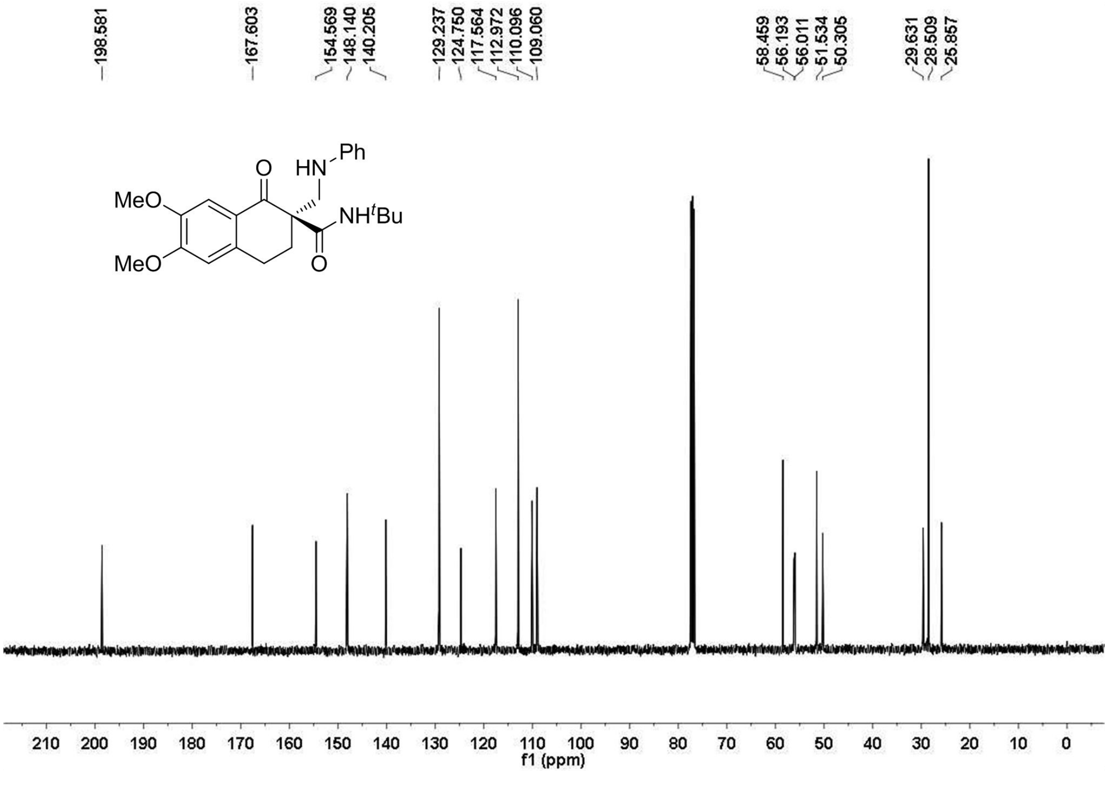

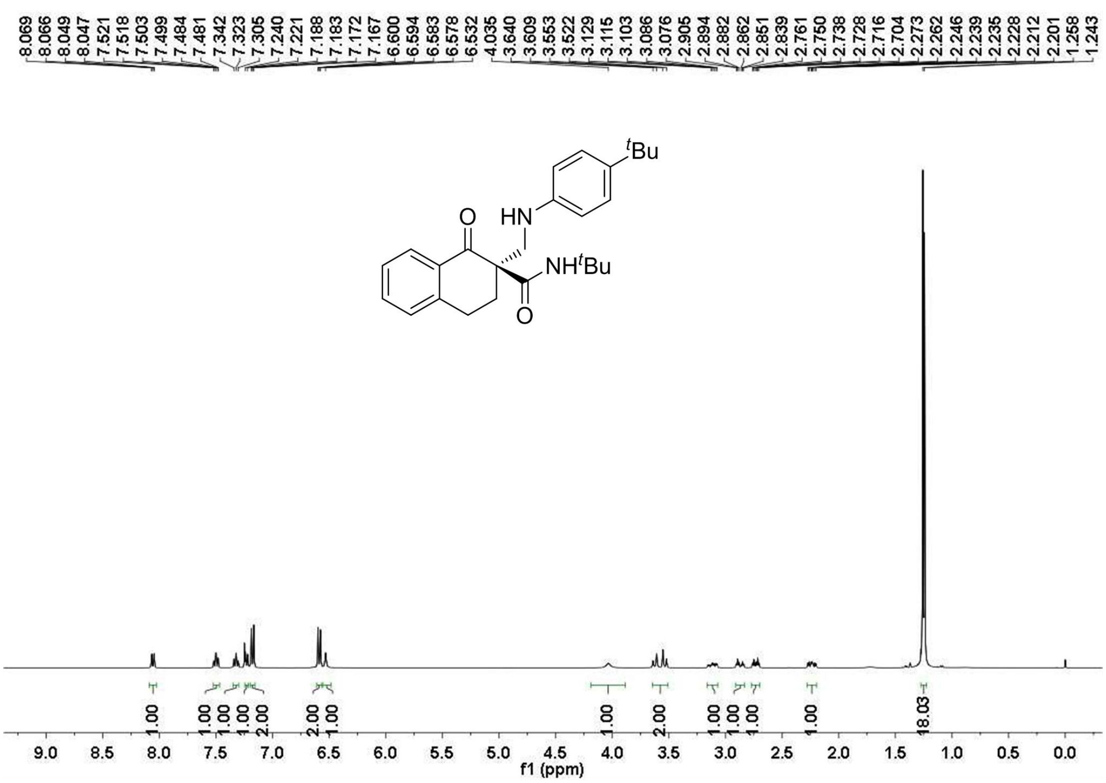
8.069
8.066
8.049
8.047
7.521
7.518
7.503
7.499
7.484
7.481
7.342
7.323
7.305
7.240
7.221
7.188
7.183
7.172
7.167
6.600
6.594
6.583
6.578
6.532
4.035
3.640
3.609
3.553
3.522
3.129
3.115
3.103
3.086
3.076
2.905
2.894
2.882
2.862
2.851
2.839
2.761
2.750
2.738
2.728
2.716
2.704
2.273
2.262
2.246
2.239
2.235
2.228
2.212
2.201
1.258
1.243
HN
^{t}Bu
O
NH^{t}Bu
O
1.00
1.00
1.00
1.00
2.00
2.00
1.00
1.00
2.00
1.00
1.00
1.00
1.00
18.03
9.0 8.5 8.0 7.5 7.0 6.5 6.0 5.5 5.0 4.5 4.0 3.5 3.0 2.5 2.0 1.5 1.0 0.5 0.0
f1 (ppm)

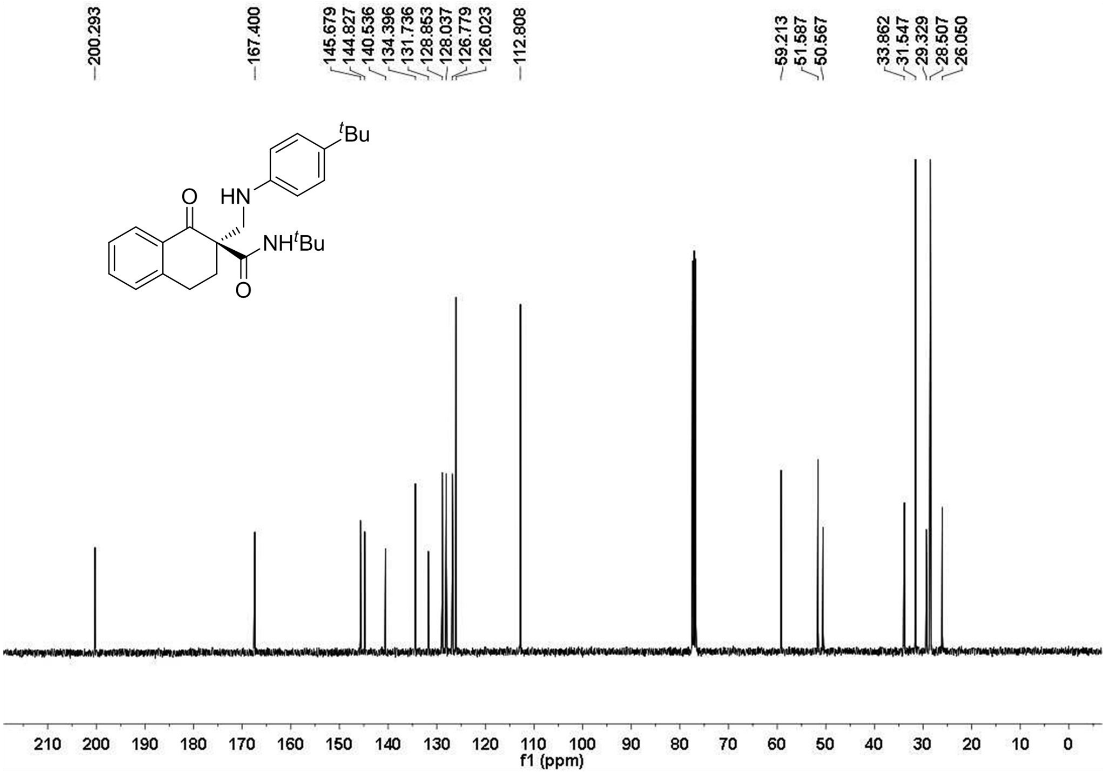
200.293
167.400
145.679
144.827
140.536
134.396
131.736
128.853
128.037
126.779
126.023
112.808
59.213
51.587
50.567
33.862
31.547
29.329
28.507
26.050
HN
^{t}Bu
O
NH^{t}Bu
O
210 200 190 180 170 160 150 140 130 120 110 100 90 80 70 60 50 40 30 20 10 0
f1 (ppm)

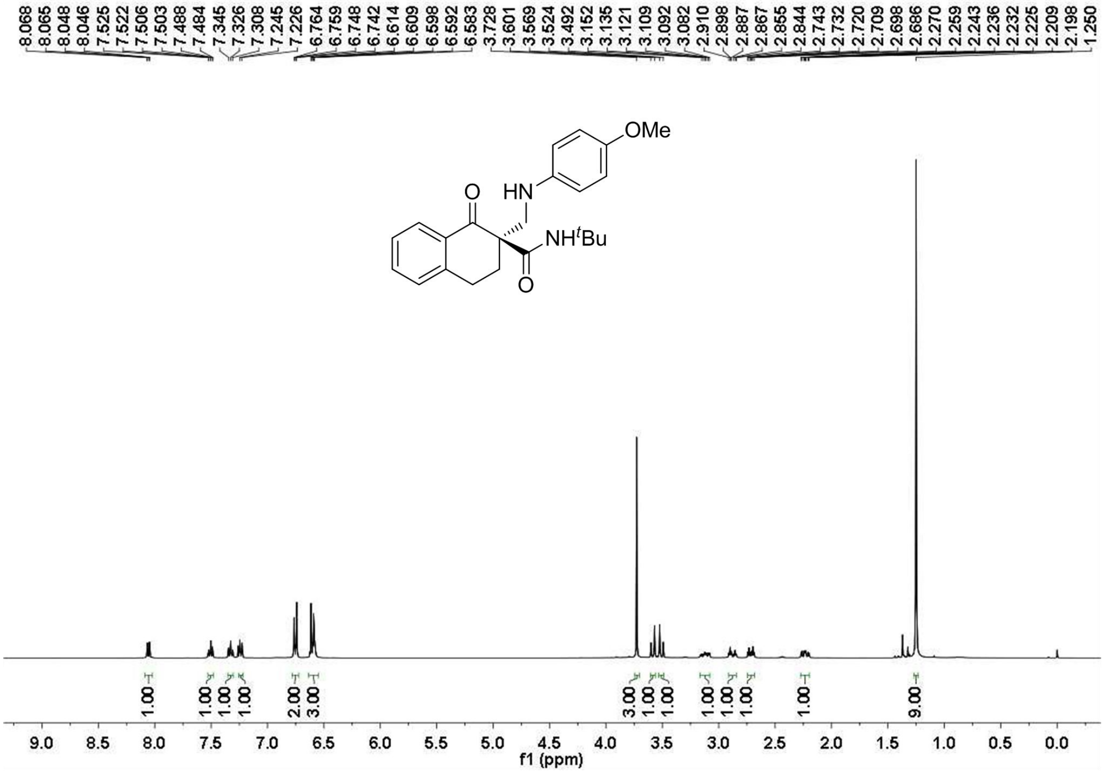

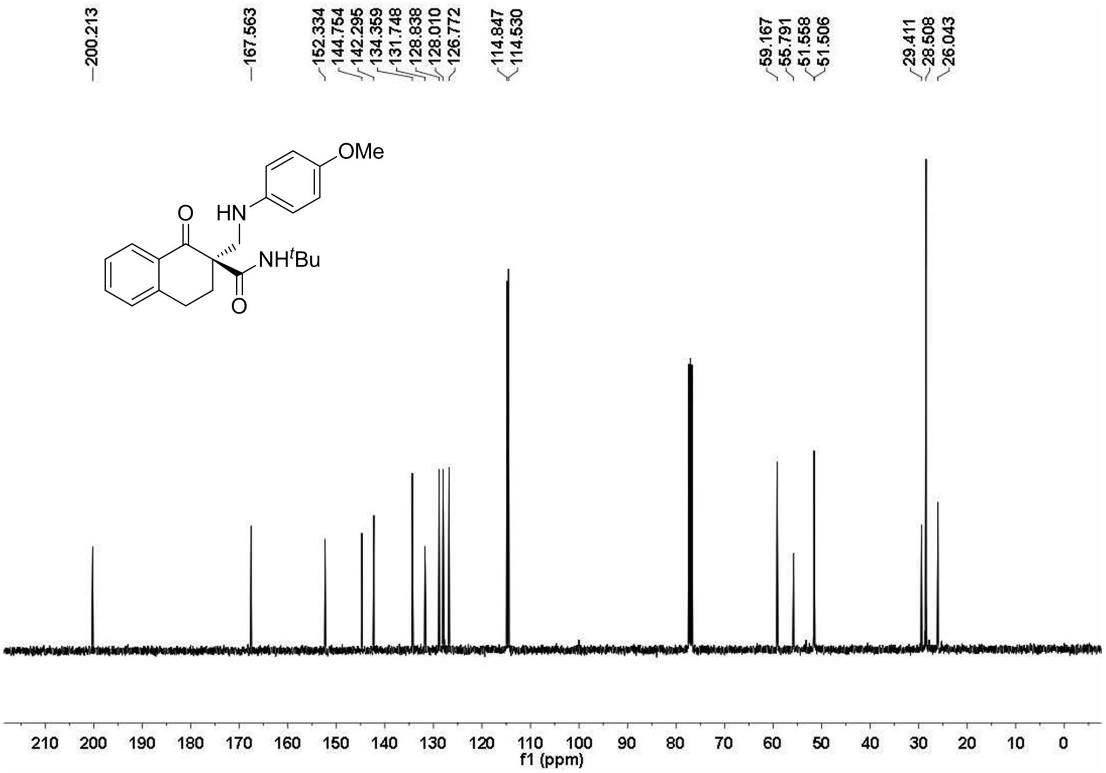

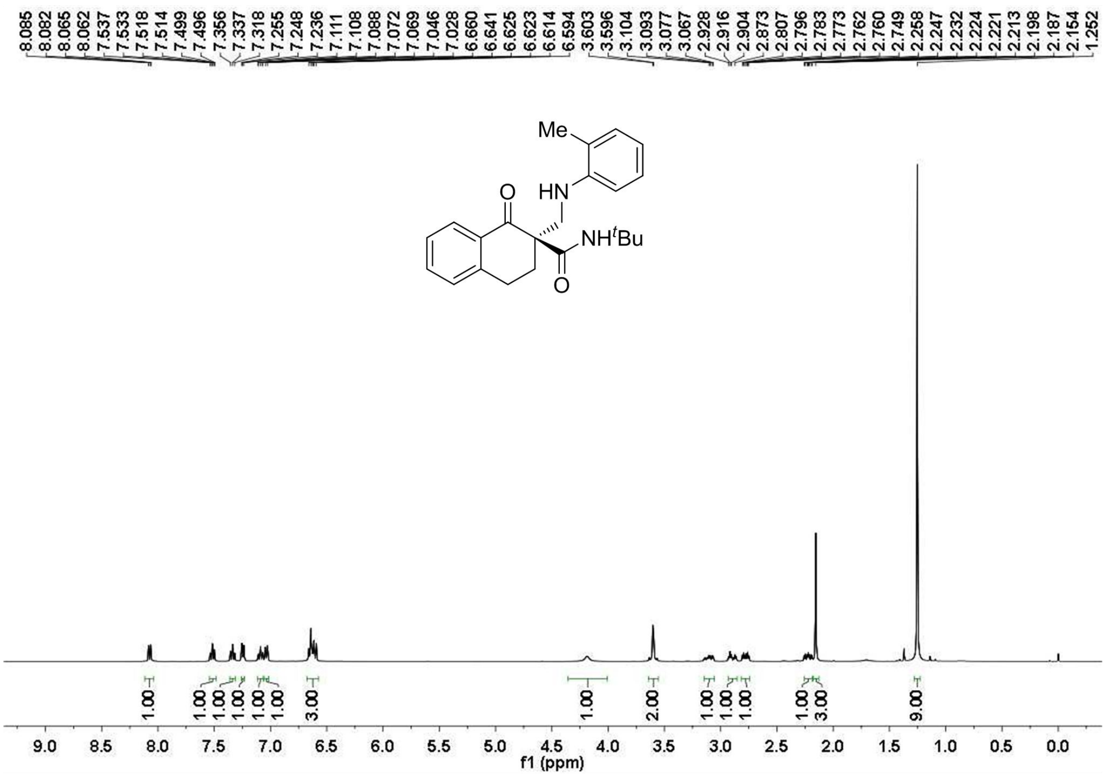

8.085 8.082 8.065 8.062 7.537 7.533 7.518 7.514 7.499 7.496 7.356 7.337 7.318 7.255 7.248 7.236 7.111 7.108 7.088 7.072 7.069 7.046 7.028 6.660 6.641 6.625 6.623 6.614 6.594 3.603 3.596 3.104 3.093 3.077 3.067 2.928 2.916 2.904 2.873 2.807 2.796 2.783 2.773 2.762 2.760 2.749 2.258 2.247 2.232 2.224 2.221 2.213 2.198 2.187 2.154 1.252
Me
HN
O
NH^{t}Bu
O
1.00 1.00 1.00 1.00 1.00 1.00 3.00 1.00 2.00 1.00 1.00 1.00 1.00 3.00 9.00
9.0 8.5 8.0 7.5 7.0 6.5 6.0 5.5 5.0 4.5 4.0 3.5 3.0 2.5 2.0 1.5 1.0 0.5 0.0
f1 (ppm)

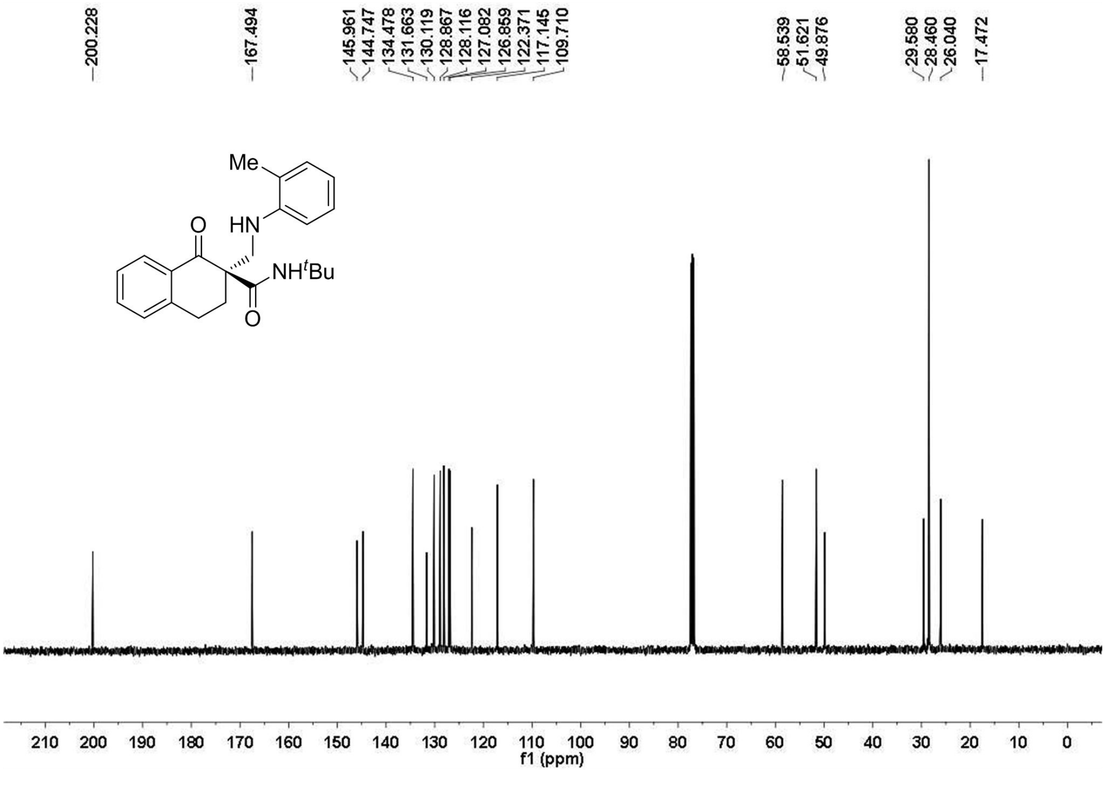

200.228 167.494 145.961 144.747 134.478 131.663 130.119 128.867 128.116 127.082 126.859 122.371 117.145 109.710 58.539 51.621 49.876 29.580 28.460 26.040 17.472
Me
HN
O
NH^{t}Bu
O
210 200 190 180 170 160 150 140 130 120 110 100 90 80 70 60 50 40 30 20 10 0
f1 (ppm)

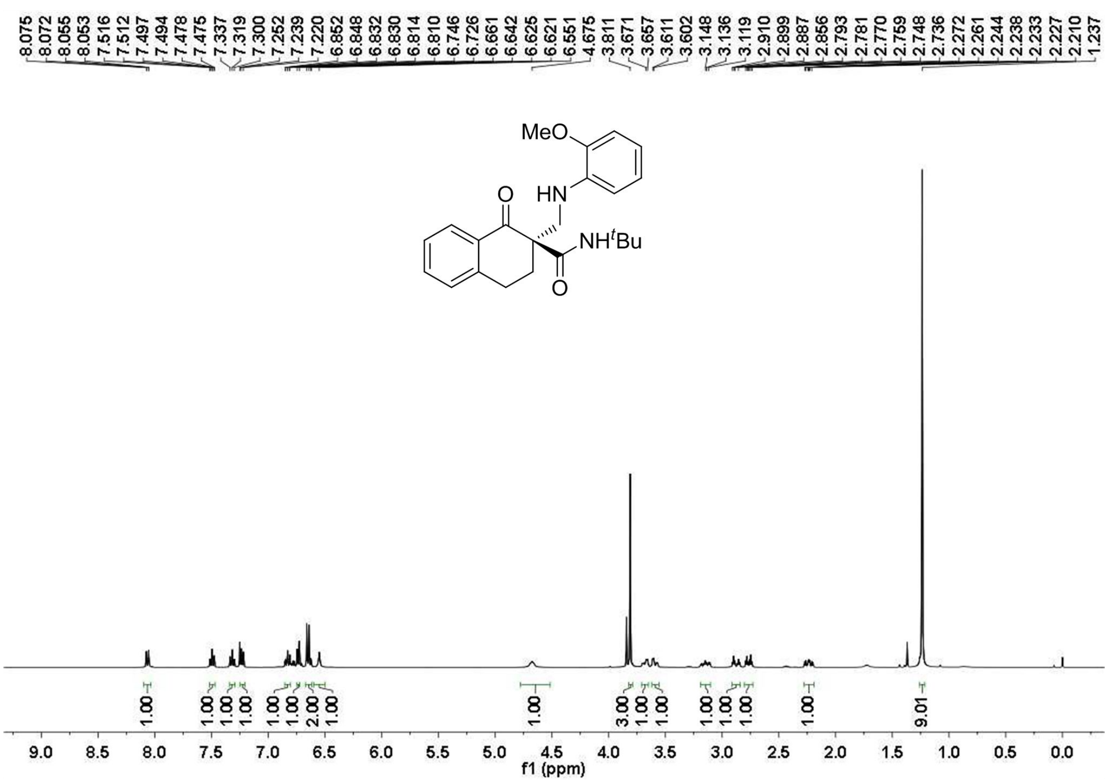
8.075 8.072 8.055 8.053 7.516 7.512 7.497 7.494 7.478 7.475 7.337 7.319 7.300 7.252 7.239 7.220 6.852 6.848 6.832 6.830 6.814 6.810 6.746 6.726 6.661 6.642 6.625 6.621 6.551 4.675 3.811 3.671 3.657 3.611 3.602 3.148 3.136 3.119 2.910 2.899 2.887 2.856 2.793 2.781 2.770 2.759 2.748 2.736 2.272 2.261 2.244 2.238 2.233 2.227 2.210 1.237
MeO
HN
O
NH^{t}Bu
O
1.00 1.00 1.00 1.00 1.00 1.00 2.00 1.00 1.00 3.00 1.00 1.00 1.00 1.00 1.00 1.00 9.01
f1 (ppm)

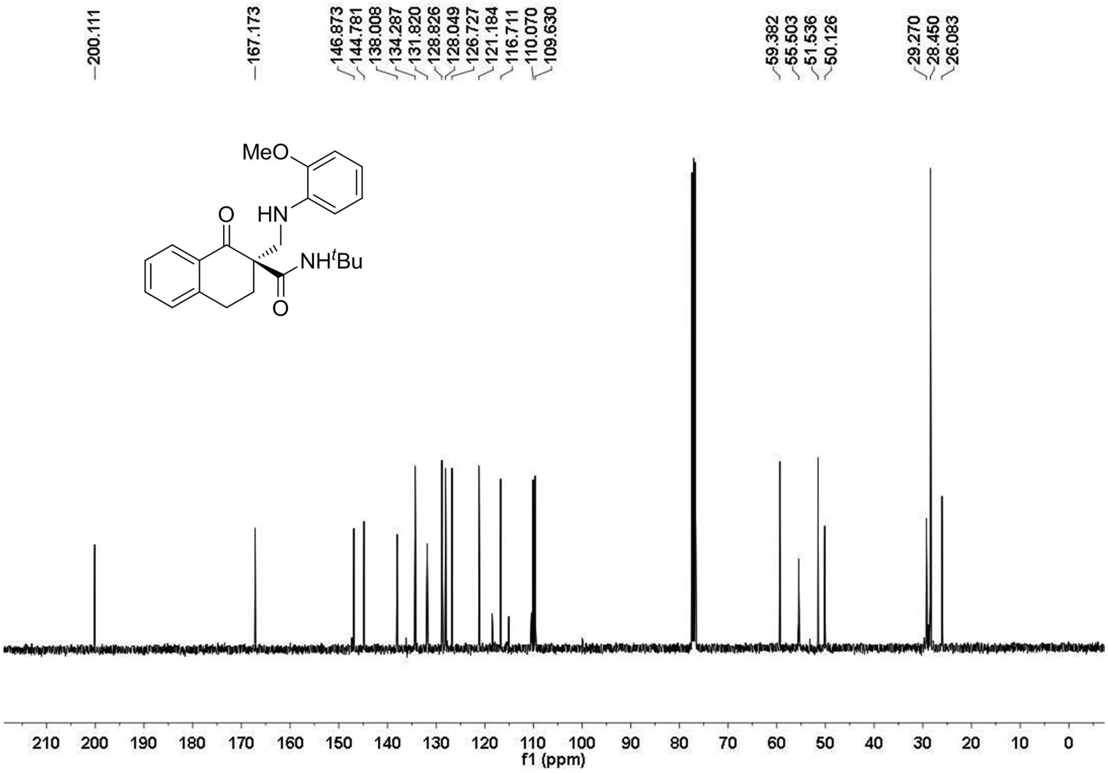
200.111 167.173 146.873 144.781 138.008 134.287 131.820 128.826 128.049 126.727 121.184 116.711 110.070 109.630 59.382 55.503 51.536 50.126 29.270 28.450 26.083
MeO
HN
O
NH^{t}Bu
O
f1 (ppm)

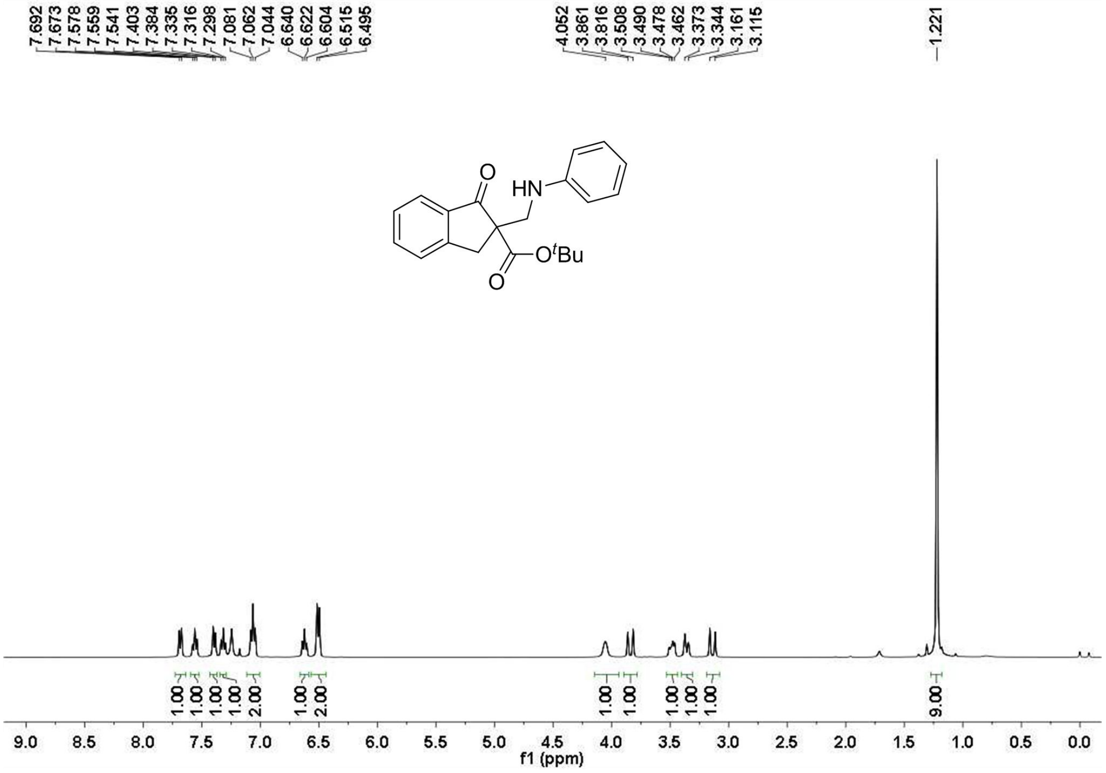
7.692
7.673
7.578
7.559
7.541
7.403
7.384
7.335
7.316
7.298
7.081
7.062
7.044
6.640
6.622
6.604
6.515
6.495
4.052
3.861
3.816
3.508
3.490
3.478
3.462
3.373
3.344
3.161
3.115
1.221
O
HN
O^{t}Bu
O
1.00
1.00
1.00
1.00
2.00
1.00
2.00
1.00
1.00
1.00
1.00
1.00
9.00
9.0 8.5 8.0 7.5 7.0 6.5 6.0 5.5 5.0 4.5 4.0 3.5 3.0 2.5 2.0 1.5 1.0 0.5 0.0
f1 (ppm)

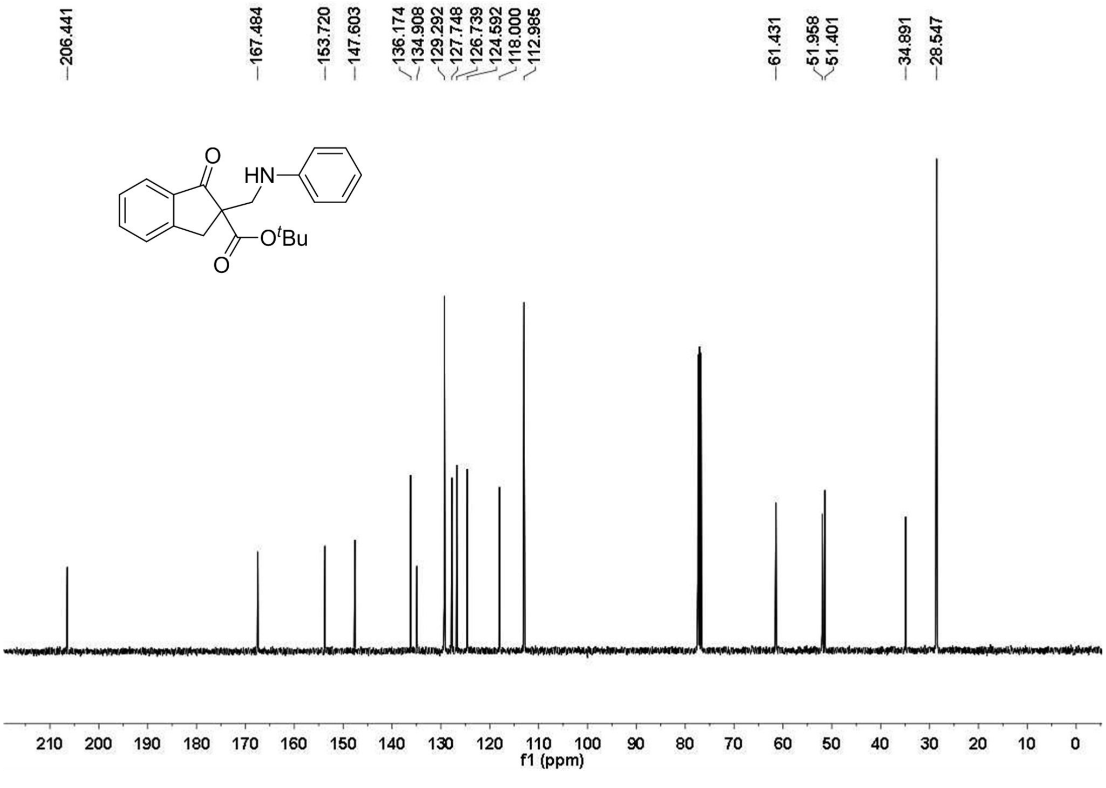
206.441
167.484
153.720
147.603
136.174
134.908
129.292
127.748
126.739
124.592
118.000
112.985
61.431
51.958
51.401
34.891
28.547
O
HN
O^{t}Bu
O
210 200 190 180 170 160 150 140 130 120 110 100 90 80 70 60 50 40 30 20 10 0
f1 (ppm)

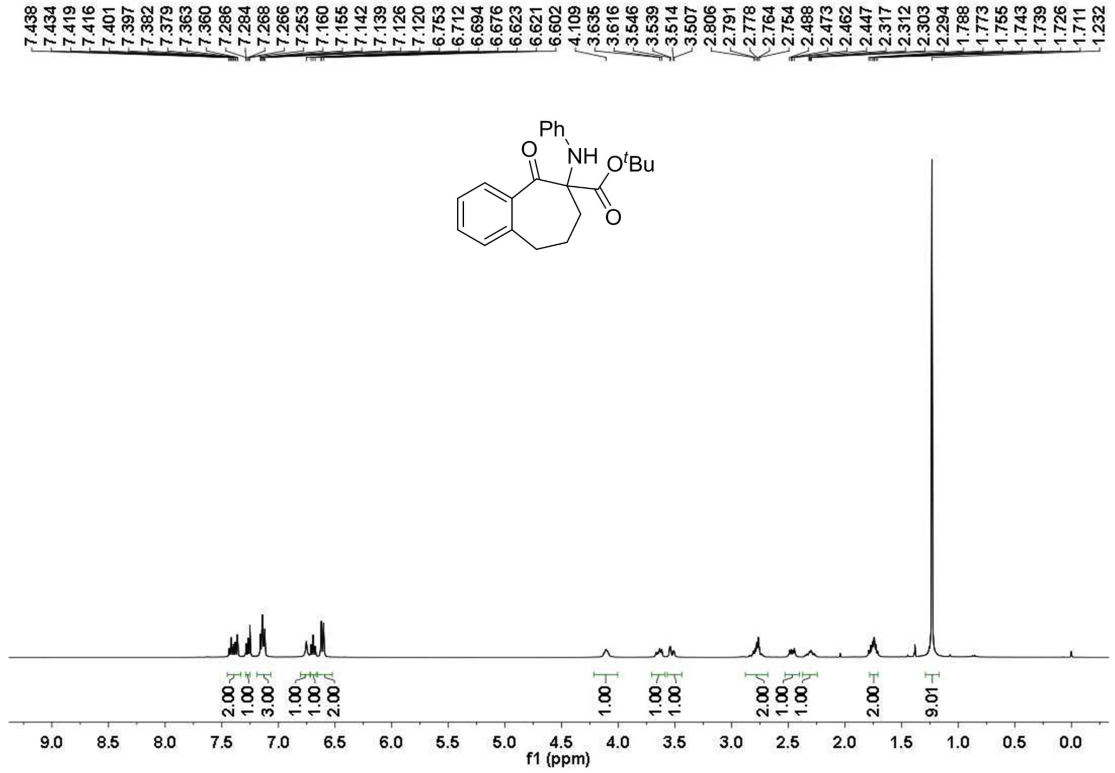
7.438
7.434
7.419
7.416
7.401
7.397
7.382
7.379
7.363
7.360
7.286
7.284
7.268
7.266
7.253
7.160
7.155
7.142
7.139
7.126
7.120
6.753
6.712
6.694
6.676
6.623
6.621
6.602
4.109
3.635
3.616
3.546
3.539
3.514
3.507
2.806
2.791
2.778
2.764
2.754
2.488
2.473
2.462
2.447
2.317
2.312
2.303
2.294
1.788
1.773
1.755
1.743
1.739
1.726
1.711
1.232
Ph
NH
O
O^{t}Bu
O
2.00
1.00
3.00
1.00
1.00
2.00
1.00
1.00
1.00
2.00
1.00
1.00
2.00
9.01
f1 (ppm)

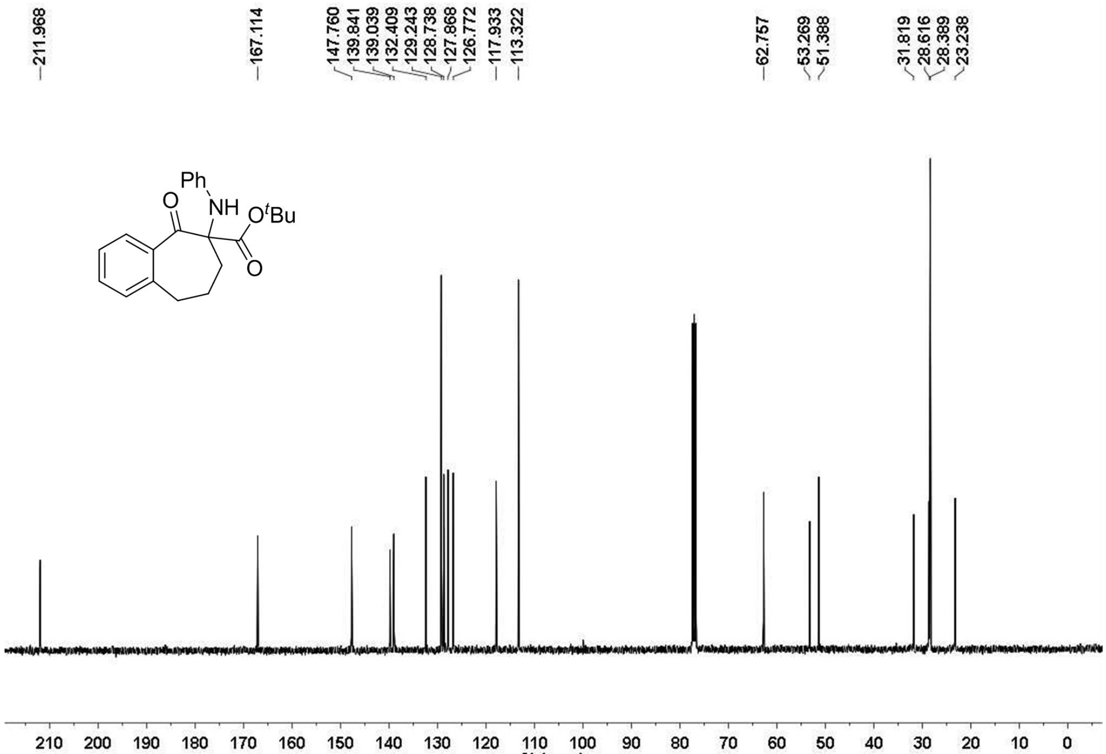
211.968
167.114
147.760
139.841
139.039
132.409
129.243
128.738
127.868
126.772
117.933
113.322
62.757
53.269
51.388
31.819
28.616
28.389
23.238
Ph
NH
O
O^{t}Bu
O
f1 (ppm)

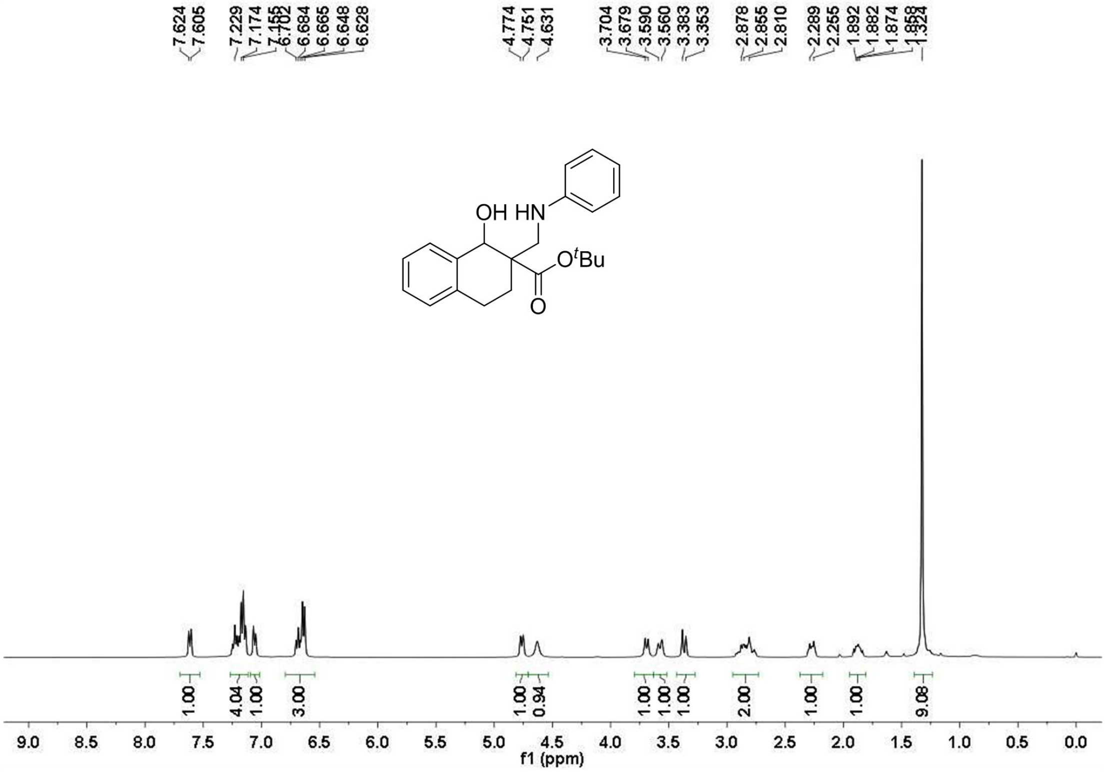

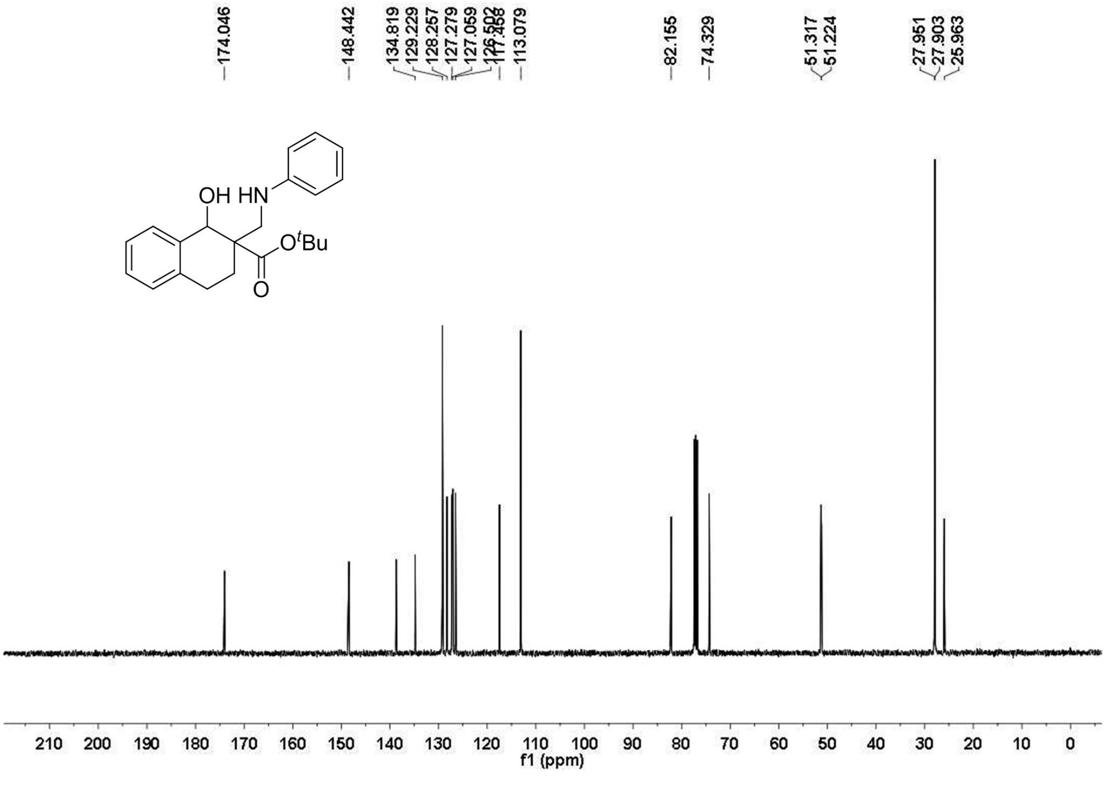

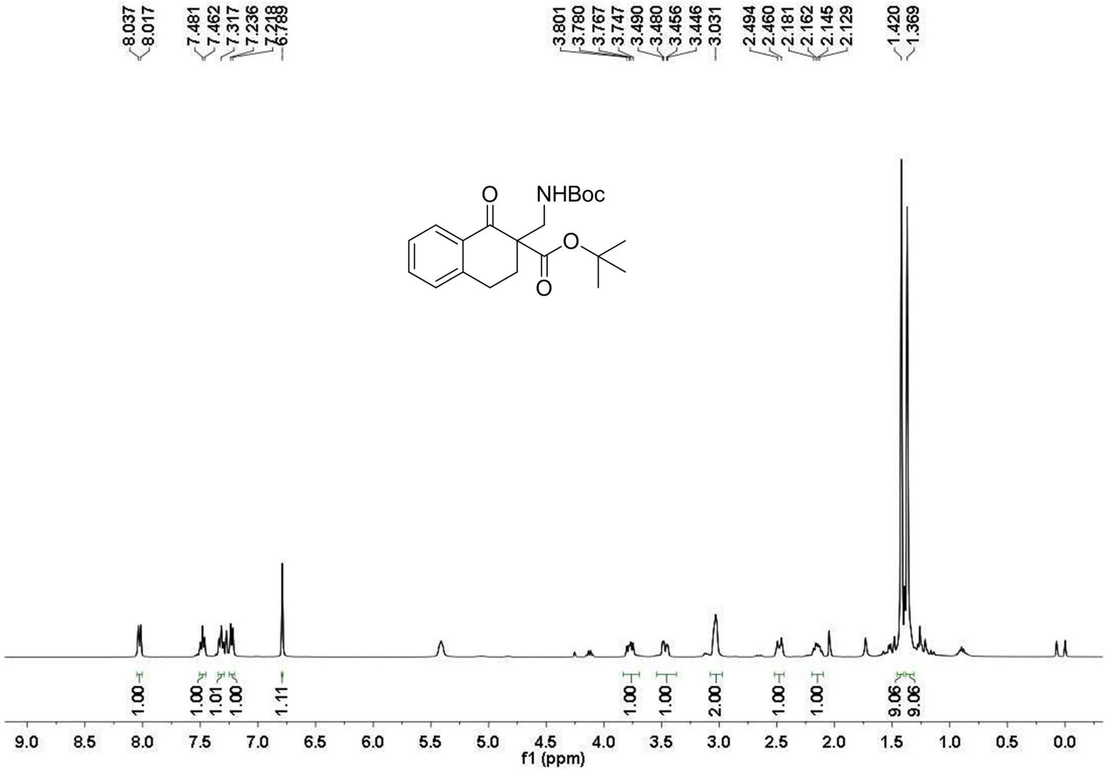

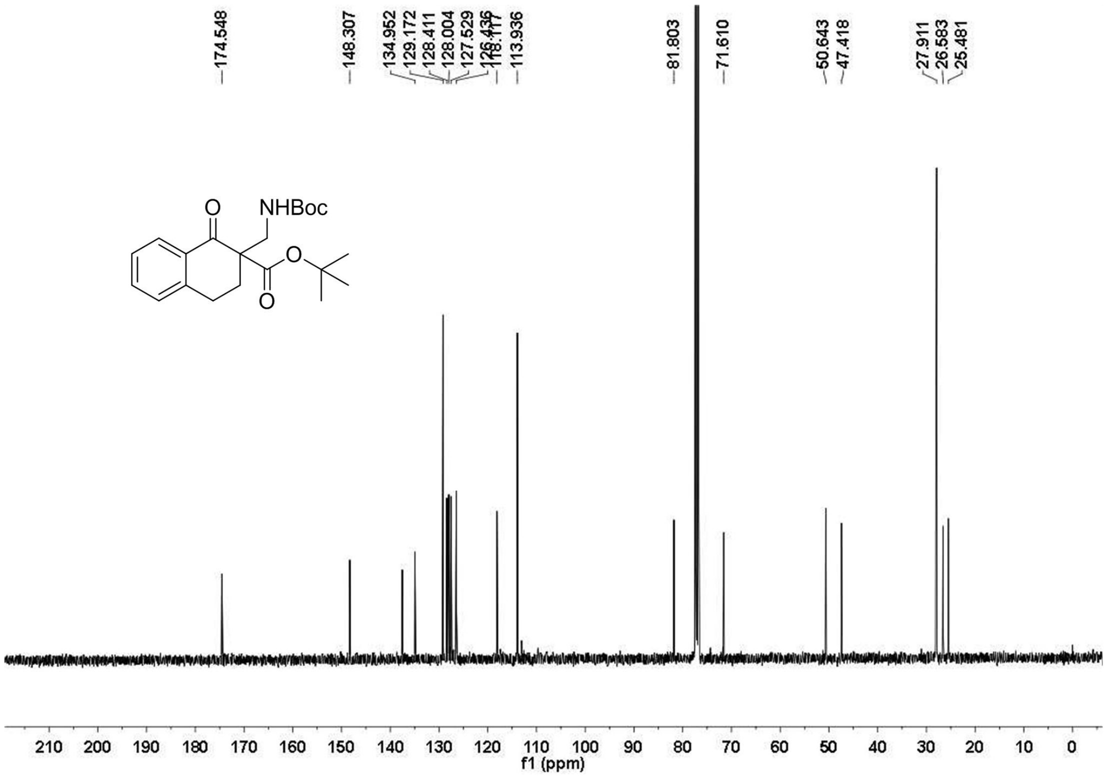

9. The NOESY spectra of 6

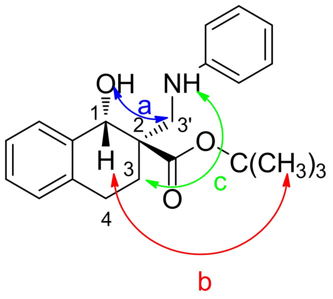

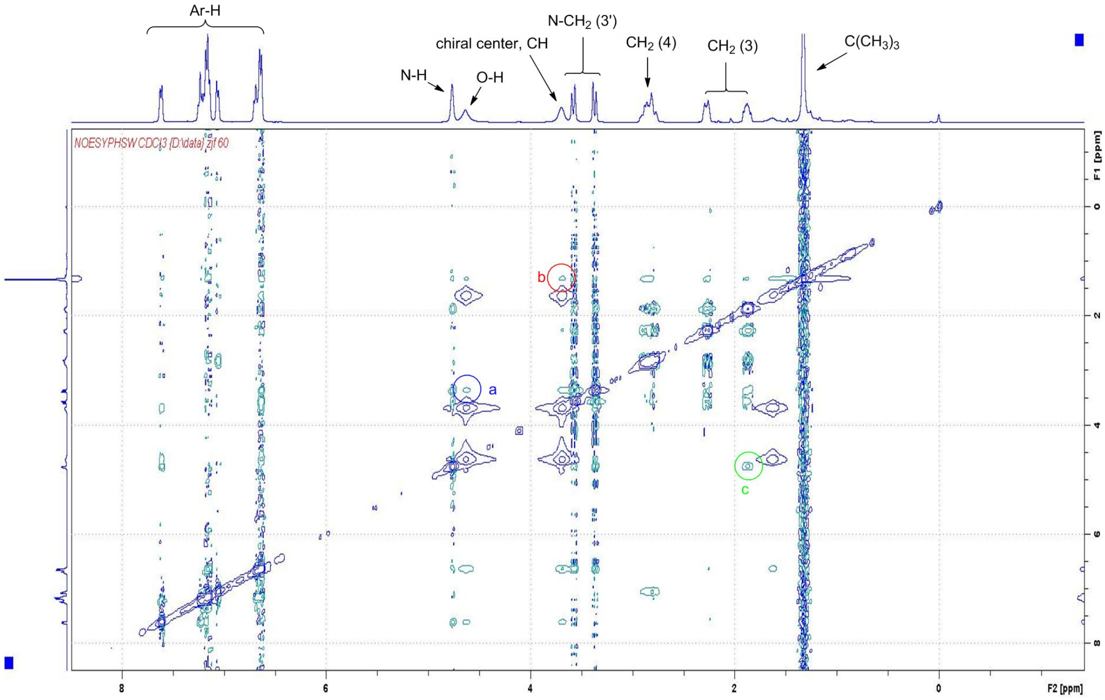

10. CD information of the products

tert-butyl 1-oxo-2-((phenylamino)methyl)-1,2,3,4-tetrahydronaphthalene-2-carboxylate (**4a**):

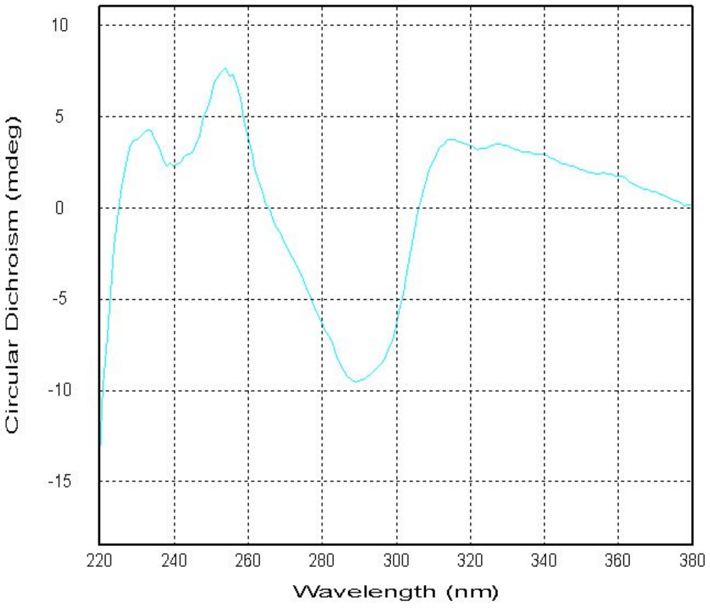

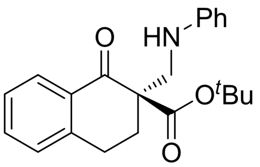

tert-butyl 5-methoxy-1-oxo-2-((phenylamino)methyl)-1,2,3,4-tetrahydronaphthal-ene-2-carboxylate (**4b**):

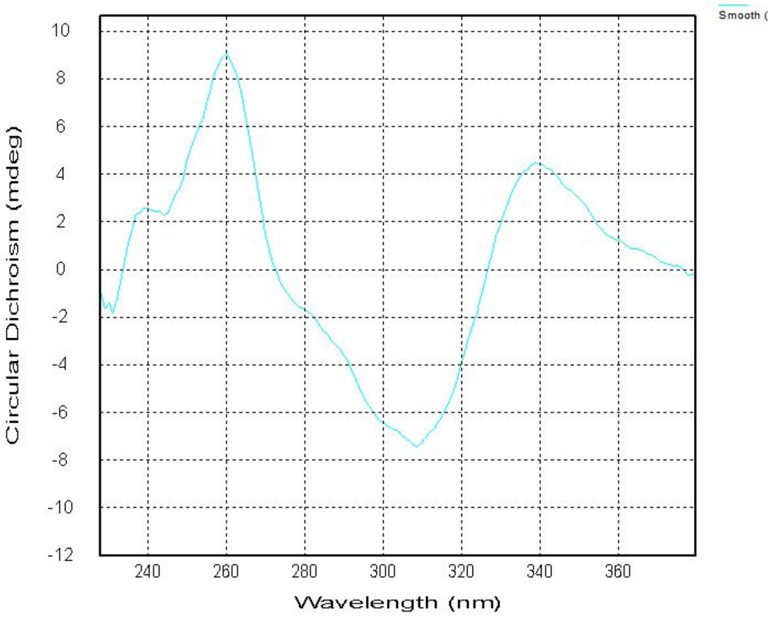

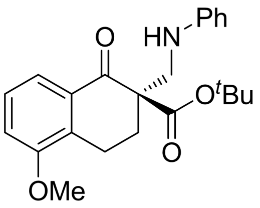

tert-butyl 6-methoxy-1-oxo-2-((phenylamino)methyl)-1,2,3,4-tetrahydronaphthalene -2-carboxylate (**4c**):

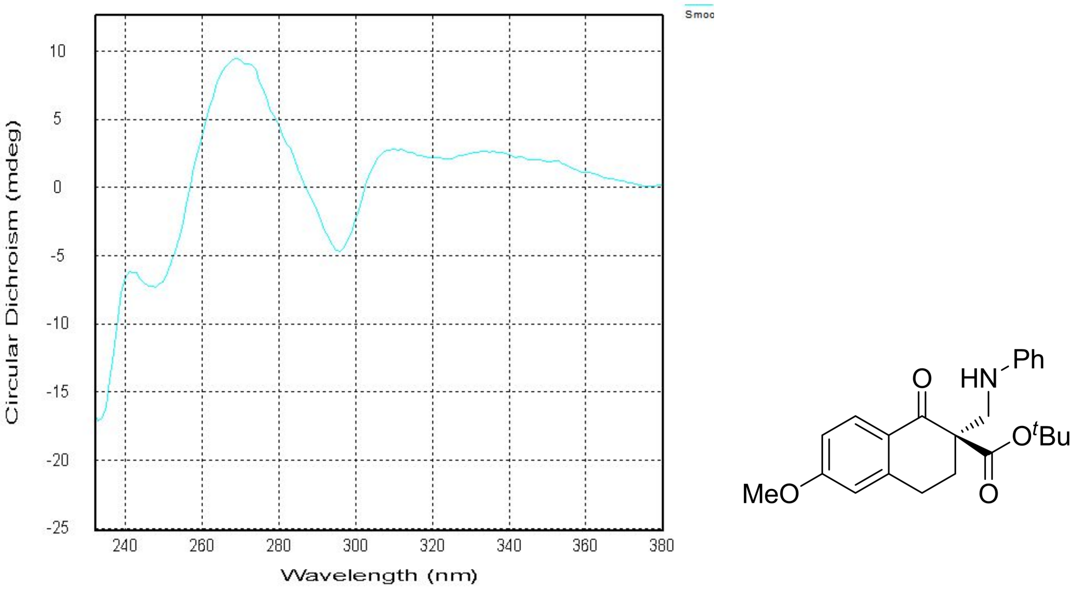

tert-butyl 7-methoxy-1-oxo-2-((phenylamino)methyl)-1,2,3,4-tetrahydronaphthalene -2-carboxylate (**4d**):

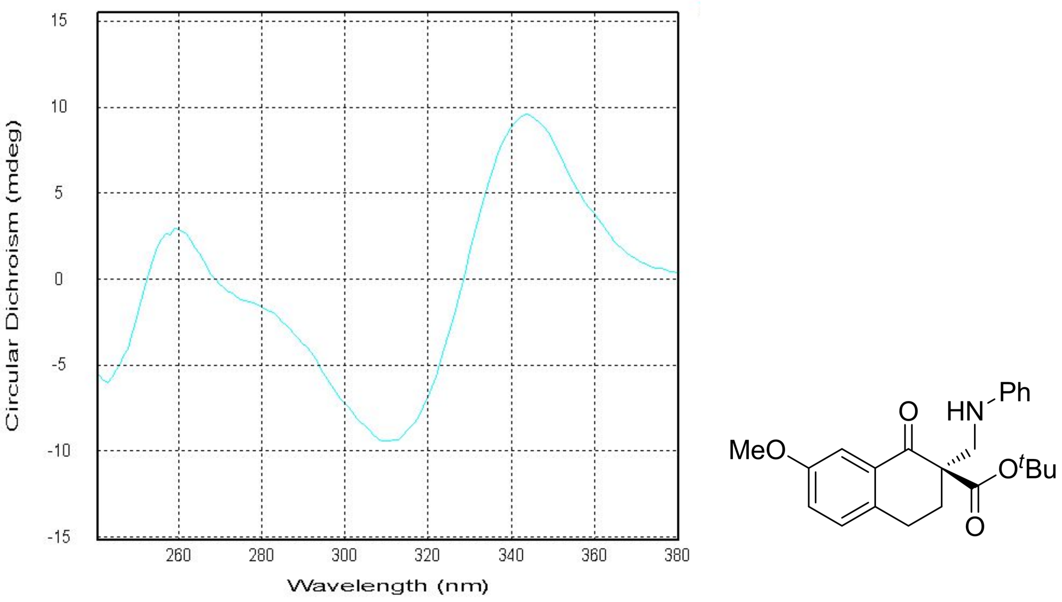

tert-butyl 7-bromo-1-oxo-2-((phenylamino)methyl)-1,2,3,4-tetrahydronaphthalene -2-carboxylate (**4e**):

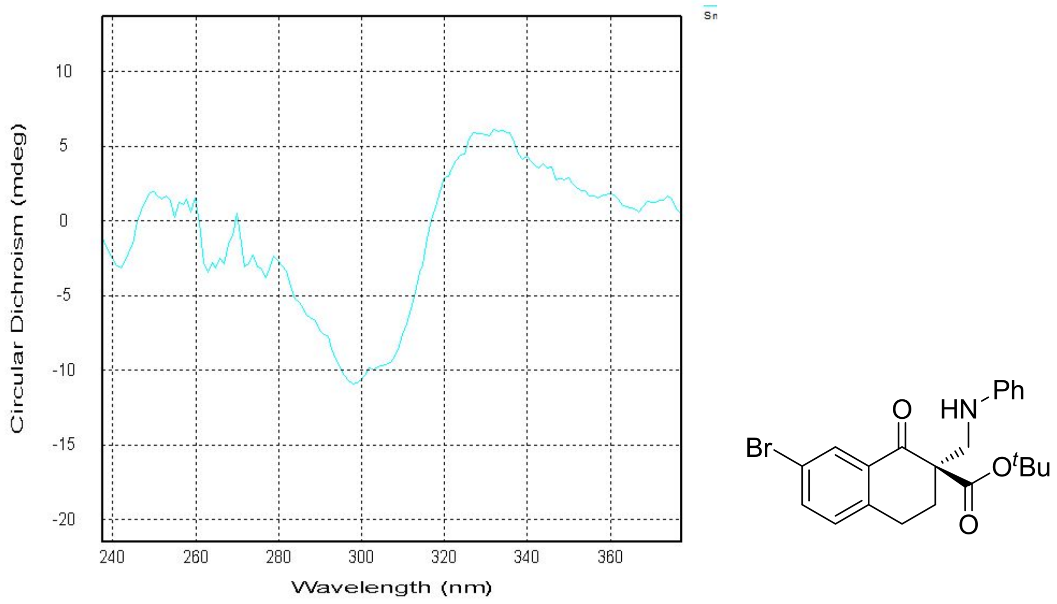

tert-butyl 5,7-dimethyl-1-oxo-2-((phenylamino)methyl)-1,2,3,4-tetrahydronaphthal -ene-2-carboxylate (**4f**):

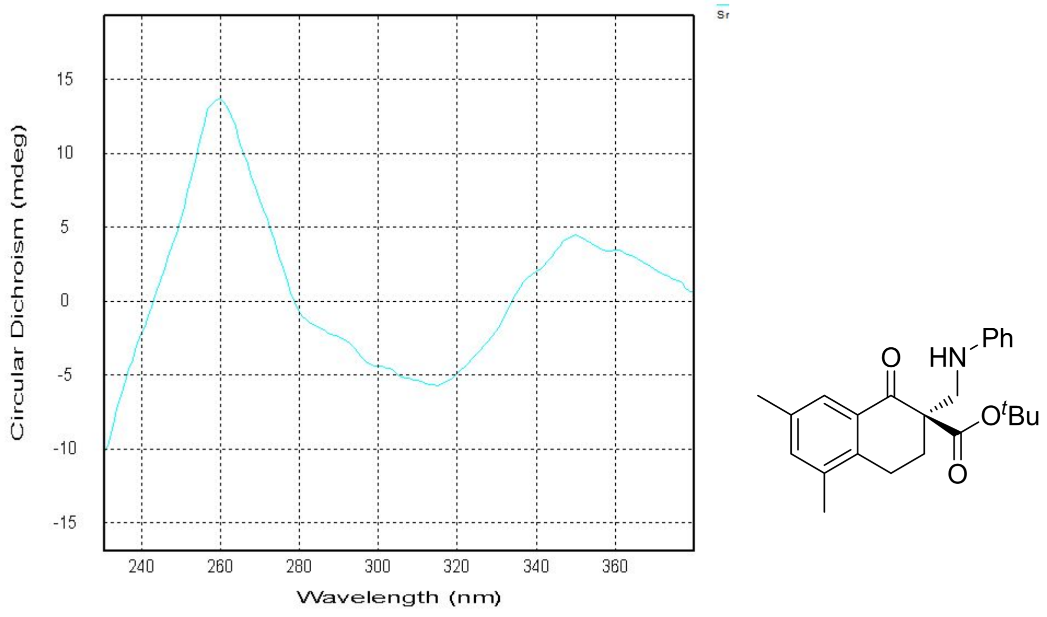

tert-butyl 2-(((4-(tert-butyl)phenyl)amino)methyl)-1-oxo-1,2,3,4-tetrahydronaphthal-ene-2-carboxylate (**4g**):

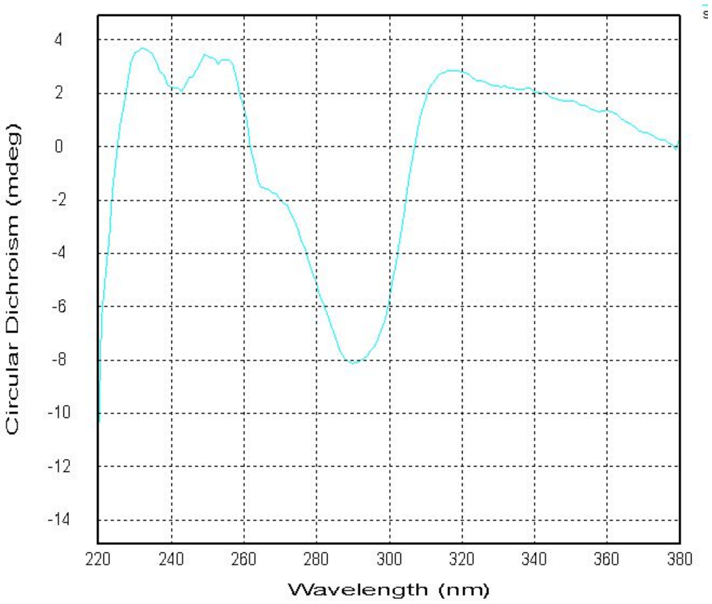

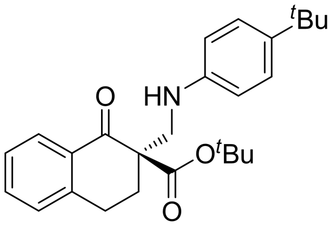

tert-butyl 2-(((4-methoxyphenyl)amino)methyl)-1-oxo-1,2,3,4-tetrahydronaphthal-ene-2-carboxylate (**4h**):

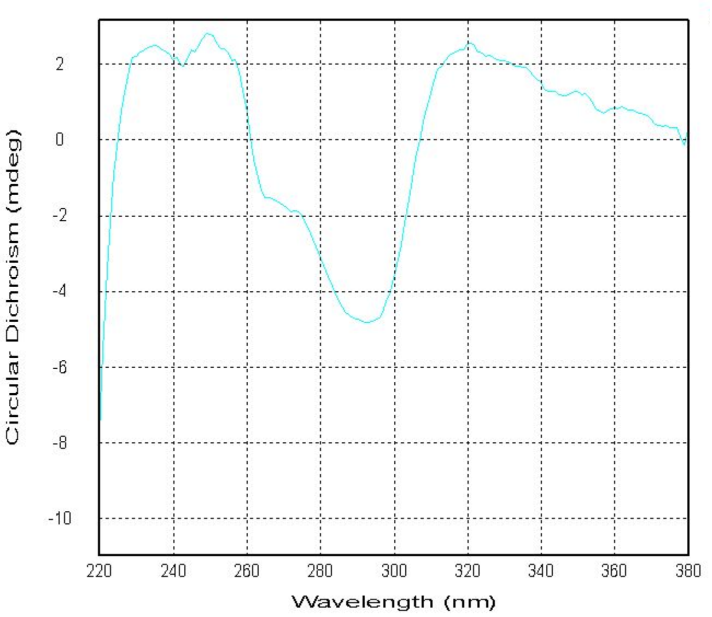

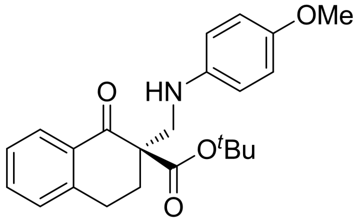

tert-butyl 2-(((4-chlorophenyl)amino)methyl)-1-oxo-1,2,3,4-tetrahydronaphthalene-2-carboxylate (**4i**):

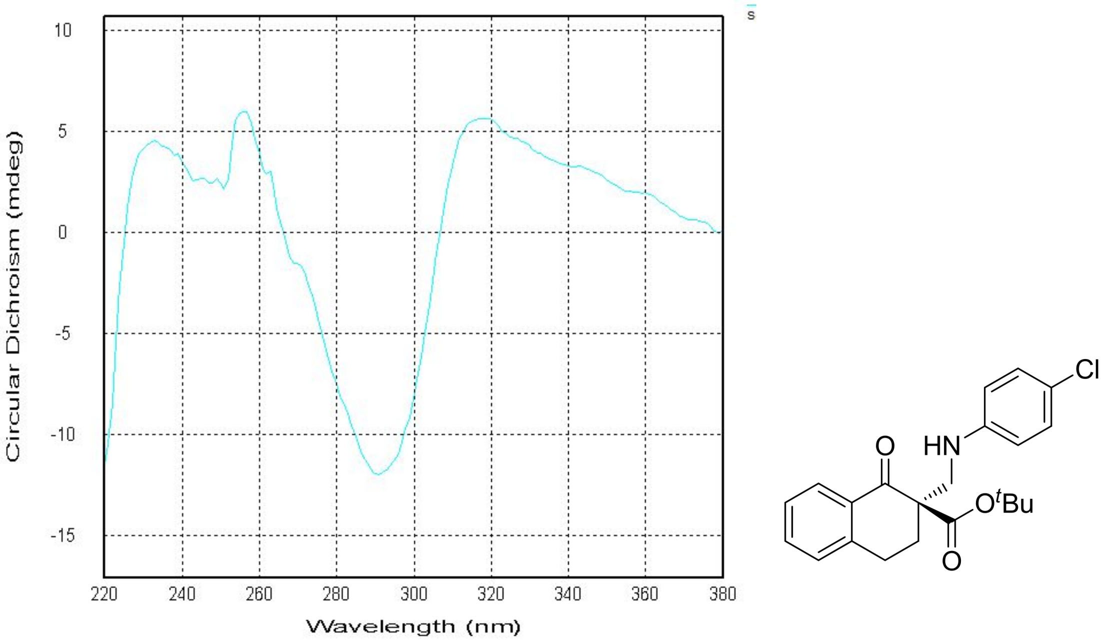

tert-butyl 1-oxo-2-((o-tolylamino)methyl)-1,2,3,4-tetrahydronaphthalene-2-carboxylate (**4j**):

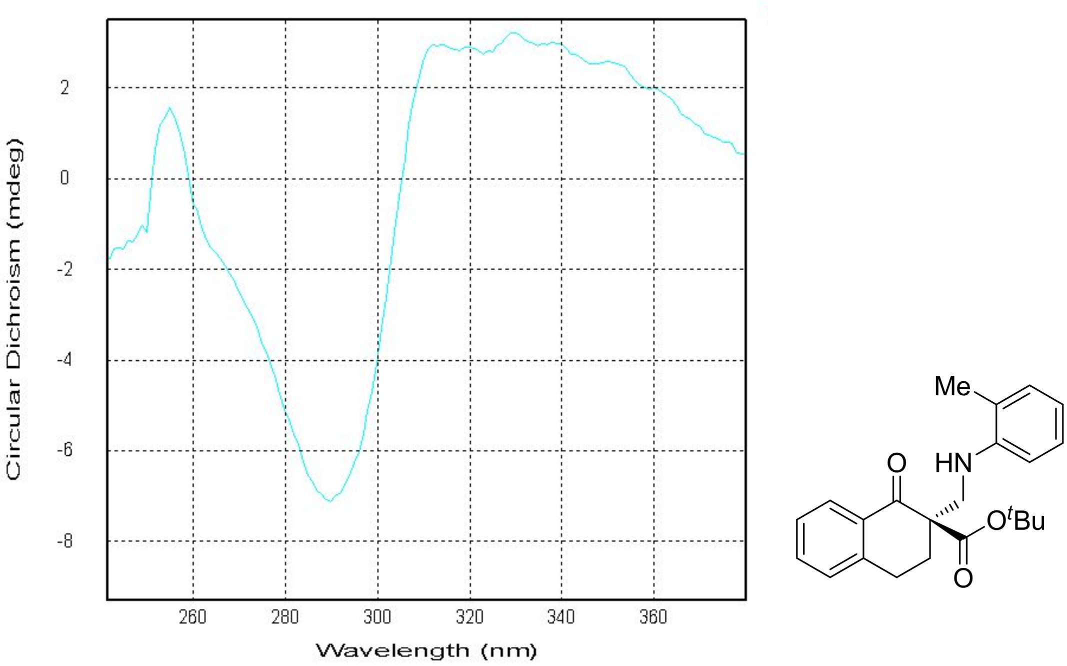

tert-butyl 2-(((2-methoxyphenyl)amino)methyl)-1-oxo-1,2,3,4-tetrahydronaphthalene -2-carboxylate (**4k**):

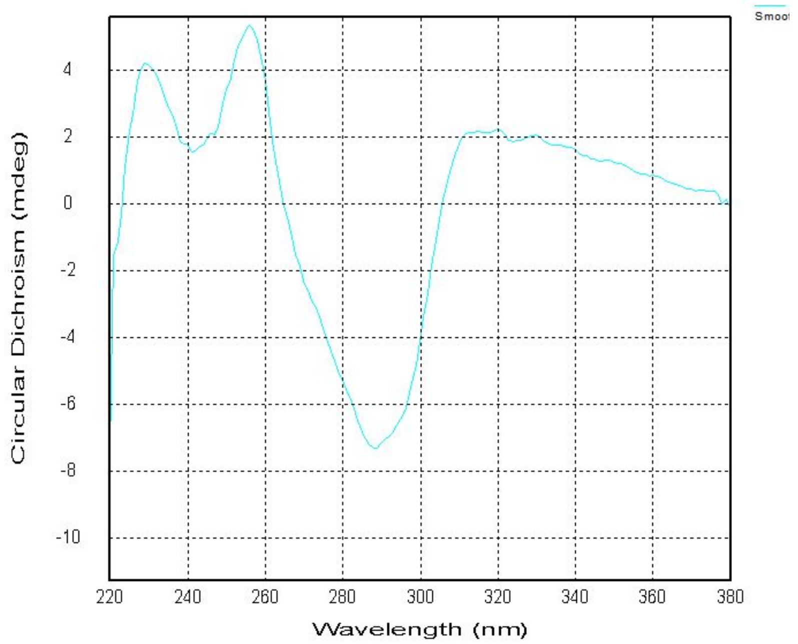

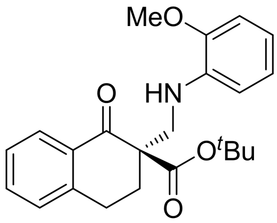

Adamantan-1-yl 1-oxo-2-((phenylamino)methyl)-1,2,3,4-tetrahydronaphthalene-2-carboxylate (**4l**):

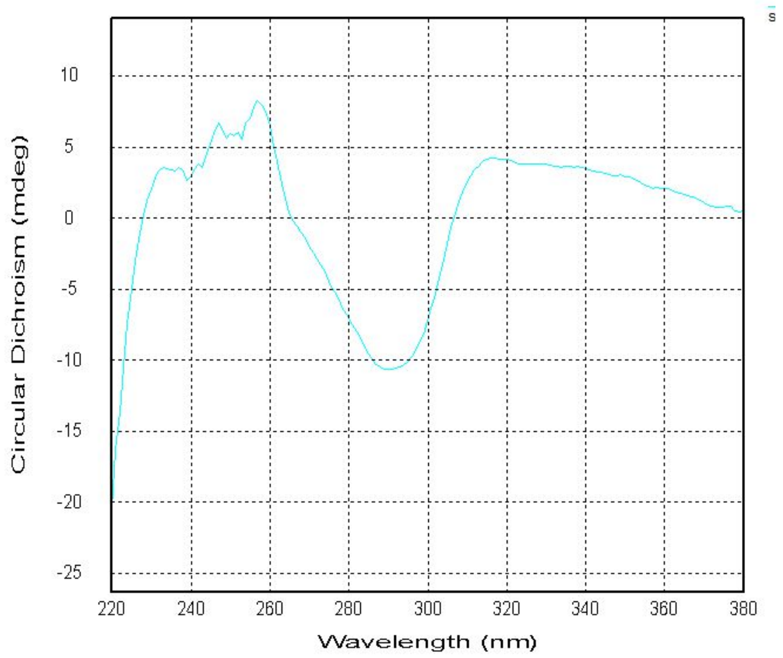

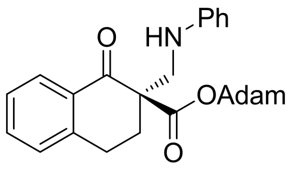

N-(*tert*-butyl)-1-oxo-2-((phenylamino)methyl)-1,2,3,4-tetrahydronaphthalene-2-carboxamide (**5a**):

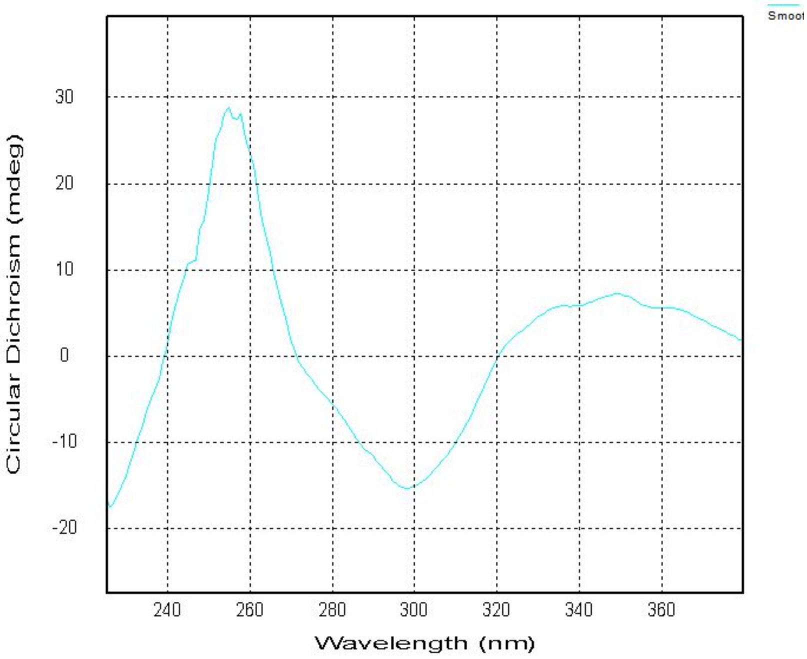

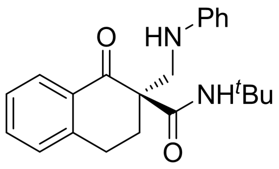

N-(*tert*-butyl)-5-methoxy-1-oxo-2-((phenylamino)methyl)-1,2,3,4-tetrahydronaphthalene-2-carboxamide (**5b**):

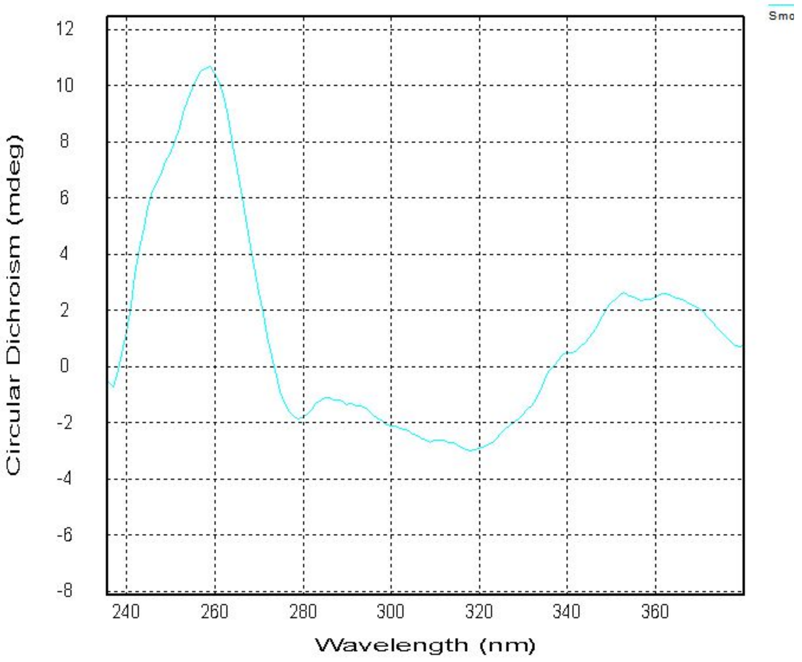

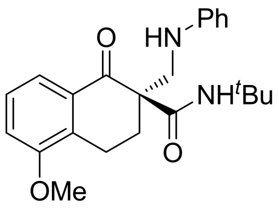

N-(*tert*-butyl)-6-methoxy-1-oxo-2-((phenylamino)methyl)-1,2,3,4-tetrahydronaphthalene-2-carboxamide (**5c**):

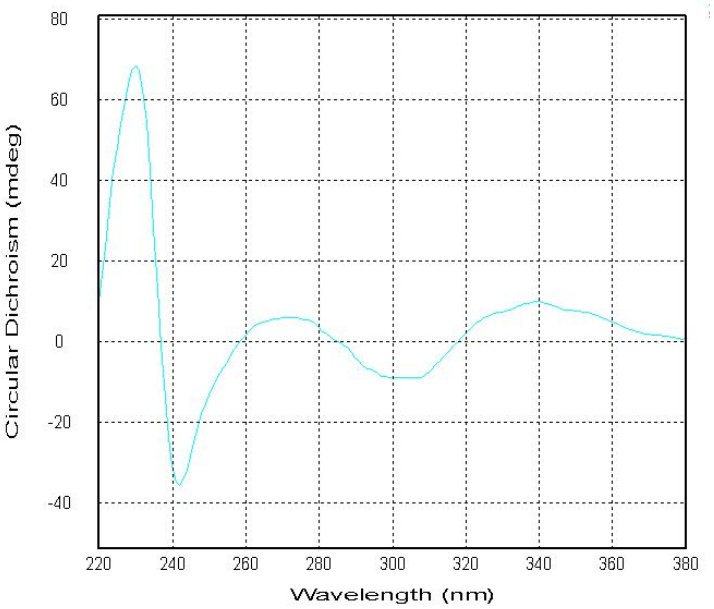

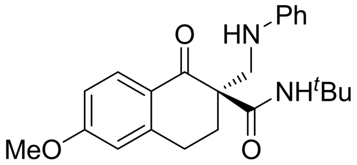

N-(*tert*-butyl)-7-methoxy-1-oxo-2-((phenylamino)methyl)-1,2,3,4-tetrahydronaphthalene-2-carboxamide (**5d**):

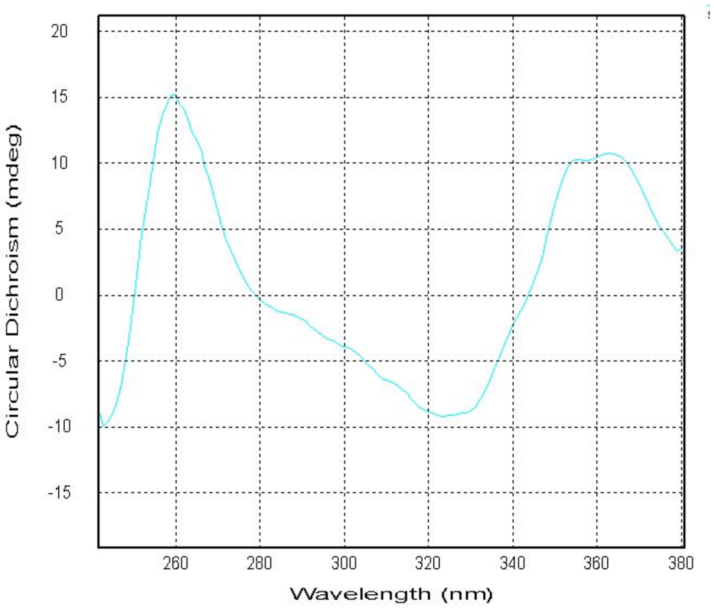

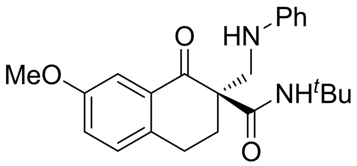

N-(*tert*-butyl)-5,7-dimethyl-1-oxo-2-((phenylamino)methyl)-1,2,3,4-tetrahydronaphthalene-2-carboxamide (**5e**):

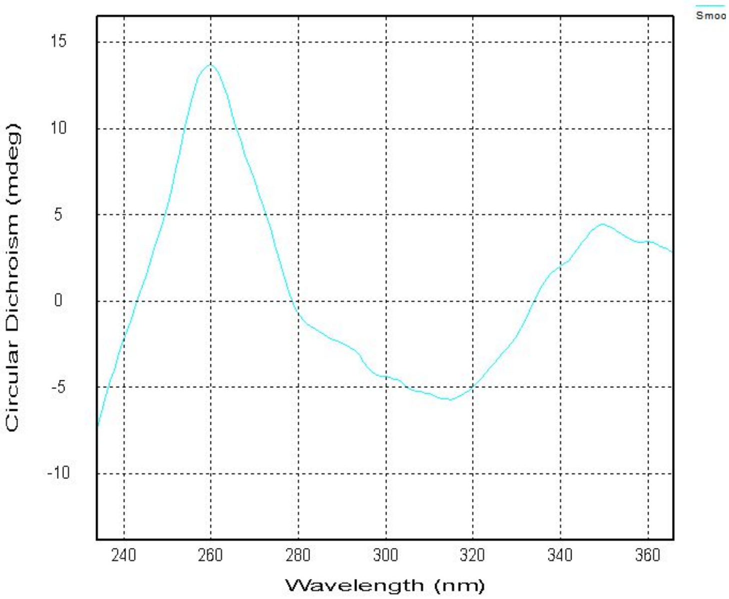

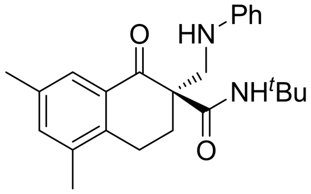

N-(*tert*-butyl)-6,7-dimethoxy-1-oxo-2-((phenylamino)methyl)-1,2,3,4-tetrahydronaphthalene-2-carboxamide (**5f**):

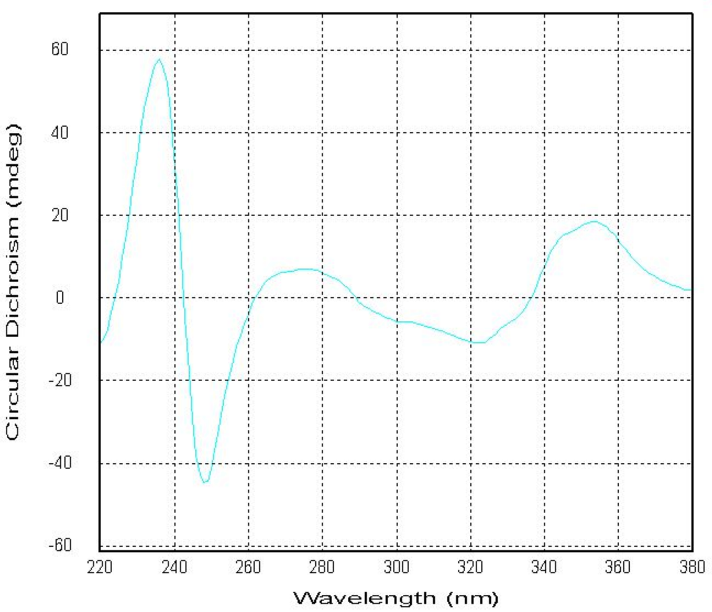

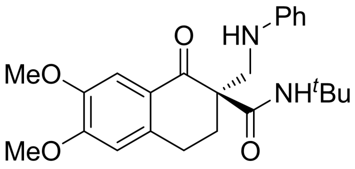

N-(*tert*-butyl)-2-(((4-(tert-butyl)phenyl)amino)methyl)-1-oxo-1,2,3,4-tetrahydronaphthalene-2-carboxamide (**5g**):

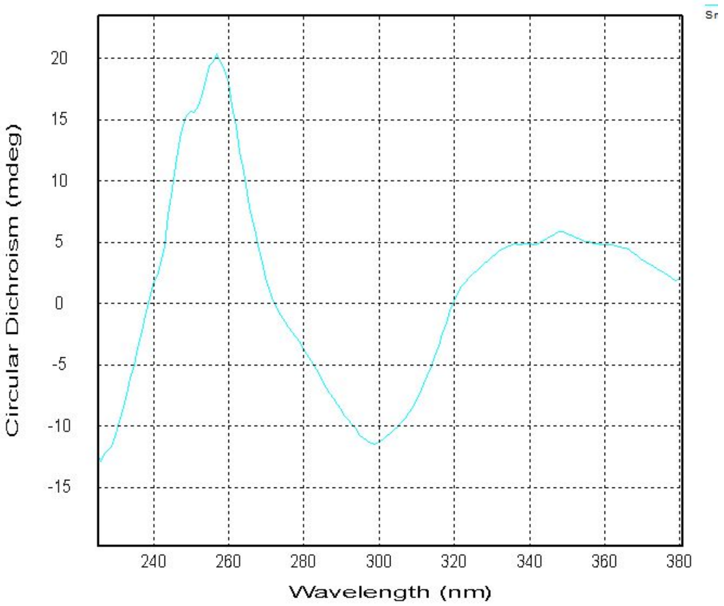

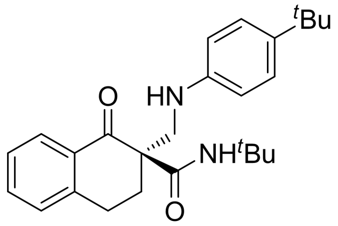

N-(*tert*-butyl)-2-(((4-methoxyphenyl)amino)methyl)-1-oxo-1,2,3,4-tetrahydronaphthalene-2-carboxamide (**5h**):

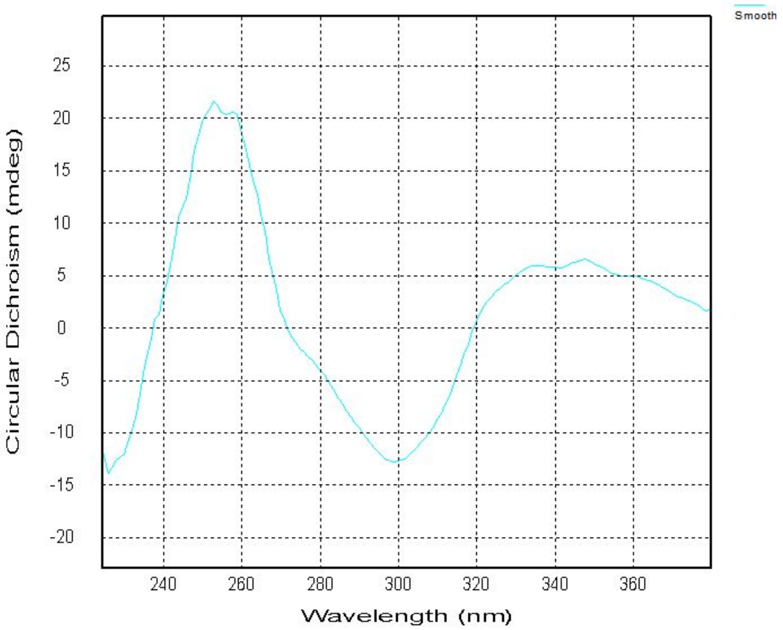

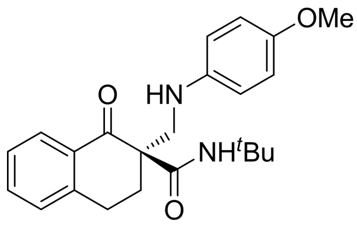

N-(*tert*-butyl)-1-oxo-2-((o-tolylamino)methyl)-1,2,3,4-tetrahydronaphthalene-2-carboxamide (**5i**):

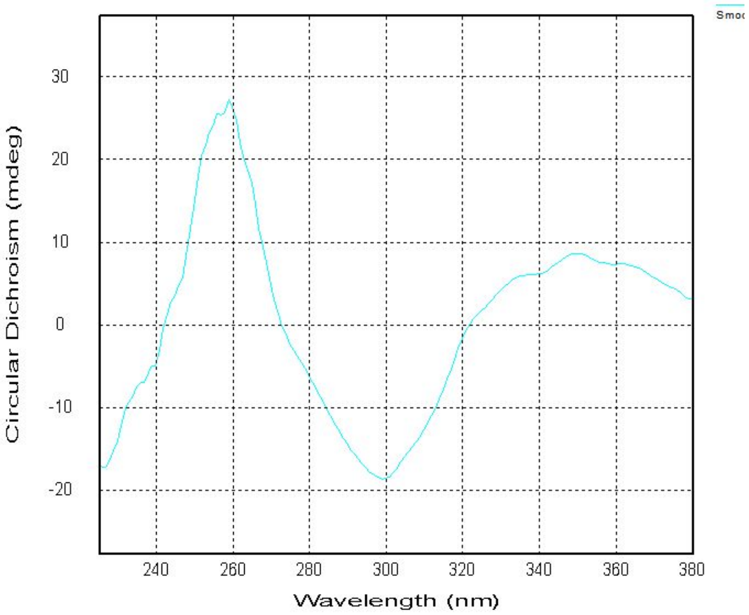

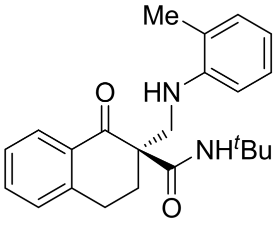

N-(*tert*-butyl)-2-(((2-methoxyphenyl)amino)methyl)-1-oxo-1,2,3,4-tetrahydronaphthalene-2-carboxamide (**5j**):

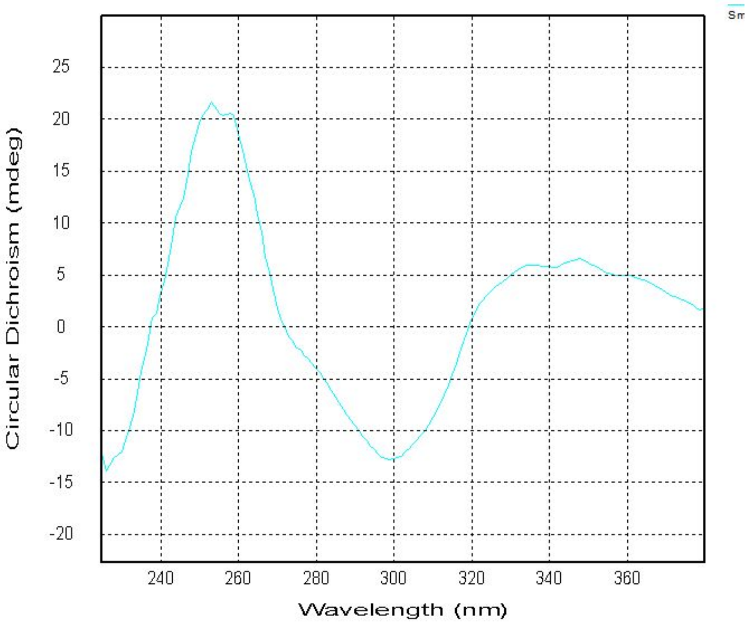

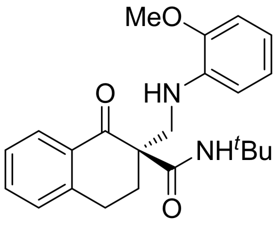

N-(*tert*-butyl)-1-oxo-2-((phenylamino)methyl)-2,3-dihydro-1H-indene-2-carboxamide (**5k**):

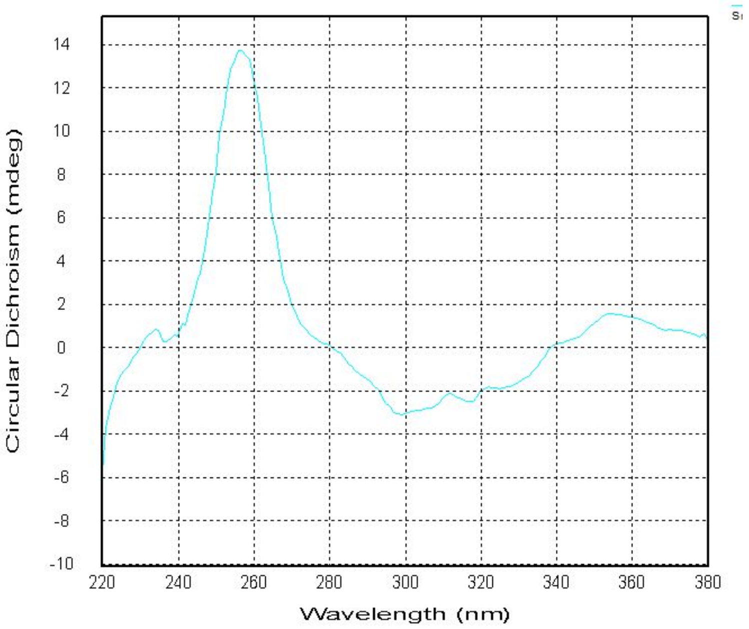

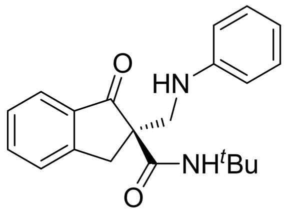

Supplement: Supplementary file 1 [file SC-008-C6SC03902B-s001.pdf]
